# A Network Model to Explore the Effect of the Micro-environment on Endothelial Cell Behavior during Angiogenesis

**DOI:** 10.3389/fphys.2017.00960

**Published:** 2017-11-27

**Authors:** Nathan Weinstein, Luis Mendoza, Isidoro Gitler, Jaime Klapp

**Affiliations:** ^1^ABACUS-Laboratorio de Matemáticas Aplicadas y Cómputo de Alto Rendimiento, Departamento de Matemáticas, Centro de Investigación y de Estudios Avanzados CINVESTAV-IPN, Mexico City, Mexico; ^2^CompBioLab, Departamento de Biología Molecular y Biotecnología, Instituto de Investigaciones Biomédicas, Universidad Nacional Autónoma de México, Mexico City, Mexico; ^3^Departamento de Física, Instituto Nacional de Investigaciones Nucleares, Mexico City, Mexico

**Keywords:** sprouting angiogenesis, network model, mechanical stress, cell differentiation, cell polarization, lateral inhibition

## Abstract

Angiogenesis is an important adaptation mechanism of the blood vessels to the changing requirements of the body during development, aging, and wound healing. Angiogenesis allows existing blood vessels to form new connections or to reabsorb existing ones. Blood vessels are composed of a layer of endothelial cells (ECs) covered by one or more layers of mural cells (smooth muscle cells or pericytes). We constructed a computational Boolean model of the molecular regulatory network involved in the control of angiogenesis. Our model includes the ANG/TIE, HIF, AMPK/mTOR, VEGF, IGF, FGF, PLCγ/Calcium, PI3K/AKT, NO, NOTCH, and WNT signaling pathways, as well as the mechanosensory components of the cytoskeleton. The dynamical behavior of our model recovers the patterns of molecular activation observed in Phalanx, Tip, and Stalk ECs. Furthermore, our model is able to describe the modulation of EC behavior due to extracellular micro-environments, as well as the effect due to loss- and gain-of-function mutations. These properties make our model a suitable platform for the understanding of the molecular mechanisms underlying some pathologies. For example, it is possible to follow the changes in the activation patterns caused by mutations that promote Tip EC behavior and inhibit Phalanx EC behavior, that lead to the conditions associated with retinal vascular disorders and tumor vascularization. Moreover, the model describes how mutations that promote Phalanx EC behavior are associated with the development of arteriovenous and venous malformations. These results suggest that the network model that we propose has the potential to be used in the study of how the modulation of the EC extracellular micro-environment may improve the outcome of vascular disease treatments.

## 1. Introduction

The circulatory system allows for the existence of large multicellular organisms, ensuring adequate oxygen and nutrient supply. Blood vessels are composed of three main layers. The outermost layer—the tunica adventitia—contains elastic fibers, collagen, and connective tissue. The middle layer—the tunica media—is comprised of smooth muscle cells, collagen, and elastin, and the innermost layer—the tunica intima—, which is exposed to the vessel lumen, is a single-cell layer of endothelium. The circulatory system is not a static structure, it adapts to the changing requirements of the body by means of vasculogenesis, arteriogenesis, and angiogenesis (Betz et al., 2016).

Vasculogenesis is a process that allows for the *de novo* formation of blood vessels. The formation of the first blood vessels in the embryo involves the differentiation of cells from the mesodermal blood islands into angioblasts, also called endothelial precursor cells (EPCs). During later development, angioblasts may differentiate from hematopoietic stem cells, multipotent bone marrow progenitor cells, myeloid cells (specifically monocytes and macrophages), side population cells, and pluripotent stem cells (Kässmeyer et al., 2009). After the differentiation of EPCs, the cells must migrate and aggregate to form a primitive vascular blood plexus. Then, for the vascular network assembly, three mechanisms have been proposed: (a) Extracellular matrix contact guidance, where the ECs are guided by collagen fibers present in the extracellular matrix and each cell may change the tension and orientation of the collagen fiber network to guide other cells, (b) Autocrine chemotaxis, where the ECs follow a morphogen (such as VEGFA) gradient and then secrete the morphogen altering the gradient to guide other cells, and (c) Cell-to-cell contact, where sprout expansion is guided by contact with multicellular elongated structures or projections of other cells (Czirok, 2013).

Arteriogenesis increases the diameter of existing blood vessels and remodels large blood vessels creating natural bypasses when necessary. Whenever, blood flow is redirected into preexisting arterioles, it creates mechanical forces. Elevated shear stress and circumferential wall stress during a long time period are strong inducers of arteriogenesis (Heil et al., 2006). The endothelium of the arteriolar connections is activated by the mechanical forces, causing monocytes to promote arteriogenesis by secreting growth factors and cytokines that increase the mitosis rate of endothelial and smooth muscle cells (Deindl and Schaper, 2005). Perivascular mast cells mediate shear stress-induced arteriogenesis by coordinating the action of T cells, neutrophils, monocytes, macrophages, and other innate immune cells by means of the secretion of cytokines and MMPs. The activation of perivascular mast cells is achieved by the increase of Nox2-derived reactive oxygen radicals, caused by neutrophil extravasation (Chillo et al., 2016).

Angiogenesis extends, maintains, and remodels existing networks of thin blood vessels, mostly capillaries. There exist two main mechanisms for angiogenesis, namely, sprouting angiogenesis (SA), and splitting angiogenesis, also known as intussusceptive angiogenesis (IA) (Gianni-Barrera et al., 2011). Alterations in blood flow and local changes in the concentration of angiogenic factors such as VEGF may trigger angiogenesis. Laminar shear stress inhibits tubule formation and migration of endothelial cells and favors intussusceptive angiogenesis, while turbulent shear stress causes an increase in cell migration and proliferation, and favors sprouting angiogenesis (Makanya et al., 2009). In skeletal muscle, VEGFA_164_ over-expression induces vascular growth by intussusception rather than sprouting (Gianni-Barrera et al., 2013).

IA occurs during physiological adaptation i.e., exercised muscles, embryonic development, and pathological situations such as tumor growth. During IA, endothelial cells extend processes into the vascular lumen from opposing walls. Once these processes contact each other, the endothelial cell junctions at the contact site are reorganized. Then, the bilayer is perforated by invading interstitial tissue, pericytes, and myofibroblasts, forming a transluminal pillar. Subsequently, pericytes, fibroblasts, and other supporting cells deposit additional collagen and other stabilizing fibers into the extracellular matrix of the pillar (Makanya et al., 2009), that may increase in girth, until it splits the blood vessel into two independent vascular segments (Patan et al., 1996, 1997). Additionally, several transluminal pillars may fuse to split a vessel or improve local hemodynamic behavior (Kurz et al., 2003). IA has three main advantages over SA: first, IA is achieved with minimal tissue degradation and reduced vascular permeability caused by mural cell detachment, second, a relatively short period of time is sufficient to achieve it, and third, only a relatively low rate of endothelial proliferation is needed (Kurz et al., 2003; Makanya et al., 2009; Gianni-Barrera et al., 2011). IA is necessary for the formation of organ-specific angioarchitecture (intussusceptive microvascular growth), the formation of vascular trees (intussusceptive arborization), angioadaptation and vascular pruning (intussusceptive branching remodeling) (Makanya et al., 2009).

**Figure 1:**
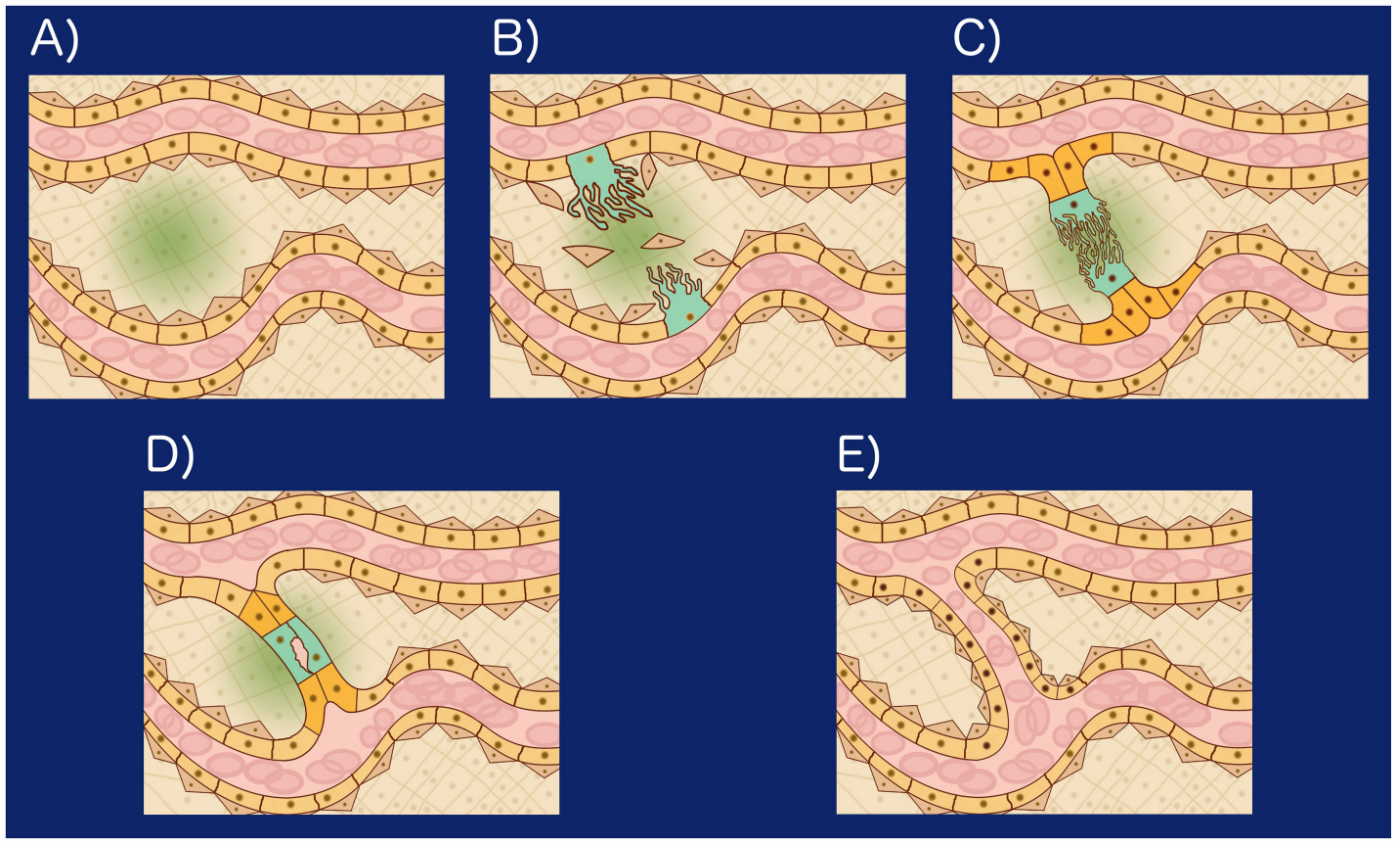
**(A)** Hypoxia induced angiogenesis: When tissue cells are exposed to a microenvironment with an insufficient concentration of Oxygen, they secrete VEGFA in a process mediated by the Hypoxia-inducible factor 1 (HIF-1). Forming a VEGFA gradient (green), **(B)** Certain epithelial cells (peach) respond to VEGFA and shear stress to become tip cells (TCs): VEGFA, ANG2, shear stress, and NO lead to endothelial cell matrix degradation, loss of pericytes (brown triangular cells). Certain EC become TCs (turquoise) and grow filopodia toward the VEGFA gradient. TCs inhibit neighboring cells from becoming TCs by Notch mediated lateral signaling and Wnt, **(C)** Stalk growth and anastomosis: The cells neighboring the TCs are induced by Notch to become Stalk cells (SCs). SCs (orange) secrete VEGFR1, reducing the concentration of VEGFA in their microenvironment, undergo Wnt mediated proliferation and elongate toward the VEGFA source resulting in stalk growth. Once a TC reaches another TC or vessel wall, it undergoes VE-cadherin and Macrophage mediated binding, initiating anastomosis, **(D)** Lumen formation: Lumen formation may occur through cord hollowing (Intracellular vacuoles fuse intracellularly to hollow out stalk cells and generate an interconnected luminal space), cell hollowing, transcellular lumen formation, and lumen ensheatment. Hemodynamic forces shape the apical membrane of SCs to form and expand new lumenized vascular tubes, **(E)** Vessel stabilization: Once a lumenized new blood vessel has formed, ECs release platelet-derived growth factor B (PDGFB). PDGFB attracts pericytes which incorporate into the vessel wall. S1P, S1PR1, ANG1, TIE2, Ephirin-B2, EPH, and TGF regulate blood vessel stabilization and maturation and are regulated by shear stress.

SA is a developmental process that results in a new connection between two existing thin blood vessels (Figure 1) and involves eight related cellular processes: (1) *Secretion of angiogenic factors*. Shear stress, or an insufficient local supply of oxygen or nutrients, may cause the cells within a tissue to secrete angiogenic factors (Forsythe et al., 1996; Song and Munn, 2011; Kumar et al., 2014). Relevant angiogenic factors include growth factors, chemokines, angiopoietins, endostatin, interferons, and NO among other molecules (Logsdon et al., 2014). (2) *Vessel destabilization*. Before a new sprout may form, pericytes, myofibroblasts, and other supporting cells must be cleared from the area of the blood vessel where the sprout will form. Also, the ECM surrounding the area must be remodeled. Blood vessel destabilization is mediated by VEGFA, ANG2, NO, and the absence of blood flow (Scharpfenecker et al., 2005; Qin et al., 2013; Korn and Augustin, 2015). (3) *Tip and stalk cell differentiation*. When certain ECs are exposed to a VEGF gradient some respond to VEGFA and shear stress to become tip cells (TCs), growing filopodia toward the VEGFA gradient. TCs induce neighbor cells to become stalk cells (SCs) by Notch-mediated lateral signaling (Blanco and Gerhardt, 2013). TCs become less sensitive to Notch signaling and SCs become less sensitive to VEGF signaling (Weinstein et al., 2015; Glass et al., 2016). (4) *Sprout elongation*. The sprout is initially formed by the TC and one or two adjacent SCs. The subsequent proliferation of both the TC and SCs together with SC elongation and rearrangement support stalk elongation toward the VEGFA source resulting in stalk growth (Betz et al., 2016). (5) *Lumen formation and expansion*. Lumen formation may occur through cord hollowing, cell hollowing, trans-cellular lumen formation, and lumen ensheathment. Hemodynamic forces shape the apical membrane of SCs to form and expand new lumenized vascular tubes (Betz et al., 2016). (6) *Anastomosis*. Vascular anastomosis is the process that allows angiogenic sprouts and blood vessels to connect. Anastomosis can occur between two sprouts, or between a sprout and a functional blood vessel. The first step in an anastomosis is the formation of a stable contact between two ECs forming a new adherens junction with two layers of apical membrane and a small luminal volume in between. The mechanism that allows the formation of a new multicellular, perfused tubes depends on the presence or absence of blood pressure (Betz et al., 2016). (7) *Vessel stabilization*. Once a lumenized new blood vessel has formed, ECs release platelet-derived growth factor B (PDGFB). PDGFB attracts pericytes, which incorporate into the vessel wall. S1P, S1PR1, ANG1, TIE2, Ephrin-B2, EPH, and TGFβ regulate blood vessel stabilization and maturation and are regulated by shear stress (Scharpfenecker et al., 2005; Qin et al., 2013; Korn and Augustin, 2015). And (8) *Pruning*. Vessel pruning is basically the process of sprout formation in reverse. The absence of blood flow, or a higher anti-angiogenic (ANG2) to angiogenic (VEGFA) factor ratio, induces small blood vessel pruning by reabsorption of ECs into the remaining vasculature. Regression of larger blood vessels involves apoptosis (Korn and Augustin, 2015; Betz et al., 2016).

Due to the enormous biological and medical importance of angiogenesis, many computational and mathematical models have been proposed to explore the molecular mechanism involved in angiogenesis control (Peirce, 2008; Qutub et al., 2009; Scianna et al., 2013; Logsdon et al., 2014; Heck et al., 2015; Qutub and Popel, 2015). Some of the relevant previous models are the following: (a) A computational model exploring the relationship between hemodynamics and angiogenesis in 2D (Gödde and Kurz, 2001). (b) A computational model of oxygen transport in skeletal muscle for sprouting and splitting modes of angiogenesis (Ji et al., 2006). (c) A model that describes and explores the progression of angiogenesis during the healing process (Vermolen and Javierre, 2012). (d) A multicellular model of the early stages of angiogenesis using finite element integration that includes chemotaxis and the interaction between tip cells and stalk cells (Bookholt et al., 2016). (e) Two Boolean models that explore the relationship between the Wnt and VEGF signaling pathways, mechanoreceptors, apoptosis, cell proliferation, and lumen formation during angiogenesis (Bauer et al., 2010; Bazmara et al., 2016). And (f) a multilevel model based on the previously mentioned Boolean models (Bazmara et al., 2015). Notably, the authors of Bauer et al. (2010) and Bazmara et al. (2015) included apoptosis in their model. We did not include apoptosis in our model because thin blood vessel pruning usually occurs by the reabsorption of ECs into the remaining vasculature and seldom involves apoptosis.

Previous models of angiogenesis focused on the role played by a few of the canonical signaling pathways. However, recent discoveries have emphasized the role of TGF signaling and its interaction with the WNT, NOTCH, VEGF/NRP1, HIF, AKT, ERK, mTOR, and TIE signaling pathways, as well as the role of HIFs, Ca^2+^, NO/eNOS, and cytoskeletal mechanoreceptors during angiogenesis. As a result, none of the previous models explore the interaction among all the aforementioned canonical pathways. It was not possible to know, then, if the biological system was sufficiently well characterized from the point of view of the molecular regulation. Hereby we present a model that integrates the largest set of canonical signaling pathways, thus allowing for a comprehensive characterization of the effect of the extracellular micro-environment on EC behavior during differentiation of ECs angiogenesis. The model presents a qualitative agreement with a large set of experimental published results, showing that the regulatory network is a faithful reconstruction of the central molecular mechanism controlling the cell behavior of endothelial cells during angiogenesis. This characteristic permits the use of the model not only to describe the wild-type development and adaptation process but also to propose targets for intervention in certain diseases. Specifically, our model suggests that favoring a micro-environment that induces Phalanx EC behavior may suffice to improve the treatment of vascular retinal disorders and vascular malformations. Thus, our model can be considered as a platform to study several molecular scenarios affecting angiogenesis.

## 2. Materials and methods

### 2.1. Molecular basis of the regulatory network

To assemble our model of the molecular network involved in angiogenesis control, we first explored how each one of the main stages angiogenesis is regulated and then explored how the molecules involved in the control of each stage interact with those that regulate the other stages. We started by exploring how the ANG/TIE signaling pathway acts as a gatekeeper of EC quiescence. Next, we inquired into the mechanisms that allow lack of sufficient oxygen or nutrients to destabilize blood vessels and trigger the angiogenic process. Then, we probed the mechanism that allows certain EC to be more sensitive to angiogenic signals by regulating VEGFR activity. Later, we analyzed how VEGF signaling activates the signaling pathways ERK1/2, PI3K-AKT, SRC, and p38 MAPK, and additionally phosphorylates STATs. After that, we inquired into the mechanisms that allow mechanoreceptors to respond to shear stress and radial stress to regulate VEGF signaling. Our ensuing action was to scrutinize the mechanism that allows the VEGF, NOTCH, WNT, and TGF signaling pathways to interact and regulate tip and stalk EC behavior. Last, we explored the mechanism that allows NOTCH and WNT to regulate EC proliferation. All those molecular mechanisms, their interactions and some of the most relevant references that describe the experimental evidence are discussed in detail in the first section of the Supplementary Material.

### 2.2. The regulatory network as a discrete dynamical system

Boolean networks are discrete dynamical systems, whose simplicity allows the attainment of biologically meaningful results, after a systematic exploration of its dynamical behavior (Dubrova and Teslenko, 2011; Azpeitia et al., 2017). In our model, most variables represent genes or proteins, some represent small molecules, and one represents a mechanical force. Each variable has an activation state, that may be active, represented by a 1, or inactive, represented by a 0. Furthermore, we use a synchronous update approach where the states of all the variables are updated simultaneously. We decided to use a synchronous update scheme in our boolean model because the computational analysis of the asynchronous update is extremely time-consuming, and it is mostly required to explore race conditions and cyclic patterns of molecular activation (Garg et al., 2008; Saadatpour et al., 2010). However, neither race conditions nor cyclic behaviors are explored with our current model.

We use definitions and notation for Boolean networks based on Azpeitia et al. (2017). Let 𝔹 = {0, 1} and $N_{\leq n}^{+}=\left\{1,2,\ldots ,n\right\}$, a set of labels. A *synchronous Boolean network with n components* is a function *f* : 𝔹^*n*^→𝔹^*n*^, where the *i-th component of f* is a function *f*_*i*_ : 𝔹^*n*^→𝔹^*n*^ such that *f*_*i*_(*x*) = *f*(*x*)_*i*_. A *state* of the network is an n-tuple *x* = (*x*_1_, *x*_2_, …, *x*_*n*_) such that *x* ∈ 𝔹^*n*^. To relate a synchronous Boolean network with a molecular network, we interpret that each component of a state *x* represents the activation state of a variable denoting a molecule included in the network. The dependency of the state on the discrete time parameter *t* is denoted as *x*(*t*) and obeys the update rule given by *f*. That is for all *t* ∈ ℤ

$$\begin{array}{l} x\left(t+1\right)=f\left(x\left(t\right)\right)=\left(f_{1}\left(x\left(t\right)\right),f_{2}\left(x\left(t\right)\right),\ldots ,f_{n}\left(x\left(t\right)\right)\right), \end{array}$$

where

$$\begin{array}{l} x_{i}\left(t+1\right)=f_{i}\left(x\left(t\right)\right). \end{array}$$

Our Boolean network model is deterministic, and any given initial state of the network reaches an attractor. A *fixed point attractor* is a state *s* ∈ 𝔹^*n*^ such that *f*(*s*) = *s*. We define *f*^*ol*^ as the *l*-th iterate of the function *f* such that *f*^*ol*^ = *f*(*f*^*o*(*l*−1)^). An *attractor* is a set of states *A* ⊆ 𝔹^*n*^, such that *f*^*ol*^(*x*) = *x* for any state *x* ∈ *A*, in other terms, there exist a positive natural number *l* ∈ ℕ^+^ = {1, 2, …} such that *f*(*x*(*t* + *l*)) = *f*(*x*(*t*)) for all *x*(*t*) ∈ *A*. Furthermore, *l* is the size of the attractor and for any $j\in {\mathbb{N}}_{<l}^{+}$, *f*(*x*(*t* + *j*)) ∈ *A*. Fixed point attractors represent stationary patterns of molecular activation, while larger attractors represent cyclic patterns of molecular activation. Additionally, we assume that each attractor represents an EC behavior.

For simplicity, we refer to the variable *x*_*i*_ by its position *i* in the n-tuple *x*. For a state *x* ∈ 𝔹^*n*^ and one of its components, say the one with label *i*, we denote by *x* ~ *i* the n-tuple resulting from replacing the value of the variable *x*_*i*_ by its complement. Given two variables *i* and *j* and the update function of variable *i*, namely *f*_*i*_, *j activates i* if there exists a pair of network states *x*, *y* that differ only in the state of activation of variable *j*, that is *y* = *x* ~ *j*, *x*_*j*_ = 0 and *y*_*j*_ = 1, such that *f*_*i*_(*y*)−*f*_*i*_(*x*) > 0. Conversely, *j inhibits i* if there exists a pair of network states *x*, *y* that differ only in the state of activation of variable *j*, that is *y* = *x* ~ *j*, *x*_*j*_ = 0 and *y*_*j*_ = 1, such that *f*_*i*_(*y*)−*f*_*i*_(*x*) < 0. An *interaction* denoted as the pair (*i, j*), *i, j* ∈ ℕ_≤*n*_ is *functional* if variable *j* activates or inhibits variable *i*. Note that according to this definition, it is possible for variable *j* to both activate and inhibit variable *i* depending on the functional context. For instance, let *C*(*t*+1) = (*A*(*t*)∧¬*B*(*t*))∨(¬*A*(*t*)∧*B*(*t*)). A activates C because if we focus on the cases where B is not active; if A is active, then C is active. A also inhibits C because if we focus on the cases where B is active; C is active only when A is not active.

### 2.3. Model assembly

Using the information described in the subsection *Molecular basis of the network*, we assembled a model of the molecular network involved in angiogenesis control. Then we encoded the model using the standardized text file format required by BoolNet (Müssel et al., 2010), an R package for the analysis of Boolean networks. The models in BoolNet format, and the R scripts we used to simulate and analyze the dynamic behavior of the model are available at https://github.com/NathanWeinstein/Angiogenesis-Model/. During the development of our model, we ensured the existence of stable or cyclic patterns of molecular activation that correspond to Phalanx (AKT+, JAGa−, NRP1−), Stalk(JAGa+, NRP1−), and Tip (NRP+, DLL4a+, AKT−) EC behavior and their reachability under certain micro-environmental conditions (**Figure 4A**); specifically:

1. (*VEGFC_Dp*−, *VEGFAxxxP*−, *ANG1*+, *Oxygen*+, *ShearStress*+, *JAGp*−, *DLL4p*−, *WNT5a*−, *WNT7a*−, *FGF*−, *IGF*−, *BMP9*−, *BMP10*−, *TGFB1*−, *VEGFC_D*−, and *AMPATP*−) induces Phalanx EC behavior.
2. (*VEGFC_Dp*+, *VEGFAxxxP*−, *ANG1*+, *Oxygen*+, *ShearStress*+, *JAGp*−, *DLL4p*−, *WNT5a*−, *WNT7a*−, *FGF*−, *IGF*−, *BMP9*−, *BMP10*−, *TGFB1*−, *VEGFC_D*−, and *AMPATP*−) induces Tip EC behavior.
3. (*VEGFC_Dp*−, *VEGFAxxxP*+, *ANG1*+, *Oxygen*+, *ShearStress*+, *JAGp*−, *DLL4p*−, *WNT5a*−, *WNT7a*−, *FGF*−, *IGF*−, *BMP9*−, *BMP10*−, *TGFB1*−, *VEGFC_D*−, and *AMPATP*−) induces Tip EC behavior.
4. (*VEGFC_Dp*−, *VEGFAxxxP*−, *ANG1*+, *Oxygen*+, *ShearStress*+, *JAGp*−, *DLL4p*+, *WNT5a*+, *WNT7a*−, *FGF*−, *IGF*−, *BMP9*−, *BMP10*−, *TGFB1*+, *VEGFC_D*−, and *AMPATP*−) induces Stalk EC behavior.

Importantly, the expected patterns of molecular activation and EC behavior transitions are based on the literature (del Toro et al., 2010; Blancas et al., 2012; Glaser et al., 2016).

#### 2.3.1. Simulation of an EC behavior transition

To simulate the transitions in EC behavior, we started with one of the states of an attractor that represents the initial EC behavior. Then, we modified the variables that represent the extracellular micro-environment (*VEGFC_Dp, VEGFAxxxP, ANG1, Oxygen, ShearStress, JAGp, DLL4p, WNT5a, WNT7a, FGF, IGF, BMP9, BMP10, TGFB1, VEGFC_D*, and *AMPATP*) without changing the other variables related to the internal state of the cell, to simulate a change of micro-environment that should lead to another EC behavior. We then calculated all state transitions until reaching another attractor that represents a new EC behavior.

#### 2.3.2. Boolean network simplification

The size of the state space of a boolean molecular network represented as a directed graph with *n* nodes —one node for each variable—, grows exponentially as 2^*n*^. Simulating and analyzing the dynamic behavior of networks containing more than a hundred nodes requires considerable computational resources. Recently, certain algorithms that use boolean satisfiability (SAT) methods capable of finding the attractors of networks with hundreds of nodes have been developed and implemented (Dubrova and Teslenko, 2011). Nonetheless, analyzing the effects of mutations, summarizing trap spaces, and analyzing the robustness of large networks is still a challenging task. However, it has been proved that it is possible to remove inputs and nodes with both an indegree and an outdegree equal to one without affecting the attractors (Saadatpour et al., 2013). Accordingly, we simplified the model by removing input nodes (nodes with an indegree equal to zero) that are either active, or inactive in all ECs, and are not part of the parameters that specify an extracellular micro-environment. Additionally, we removed output nodes (nodes with outdegree equal to zero). Further, we used edge contraction to merge intermediary nodes (nodes that have either an indegree or outdegree equal to one) that are not transcription factors. The edge contraction operation involves the removal of an edge *e* (from *u* to *v*) and the merger of its two incident vertices, *u* and *v*, into a new vertex *w*. We assigned to *w* the name of *u* if *v* was only regulated by *u*, in this case we substituted *v*(*t*) for *u*(*t*) if e was positive or ¬*u*(*t*) if *e* was negative in the components of *f* that correspond to the variables originally regulated by *v*. When *u* only regulated *v*, we assigned to *w* the name of *v* and in *f*_*v*_ we substituted *u*(*t*) with *f*_*u*_, that is, *f*_*v*_(…, *u*(*t*), …) becomes $f_{v}^{\prime }\left(\ldots ,f_{u},\ldots \right)$. These operations allowed us to simplify our model without eliminating feedback circuits. This is relevant because to a large extent, feedback circuits determine the dynamic behavior of a boolean network (Azpeitia et al., 2017). The authors of Veliz-Cuba (2011) and Naldi et al. (2011) studied when the attractors are preserved after similar simplification processes. Additionally, we verified that the EC behaviors and transitions based on the literature were preserved after the simplification process. Further, we also verified that in both the detailed and the simplified model, all single gain and loss of function mutations have a similar effect on the EC behaviors and transitions based on the literature (Supplementary Figures 15, 16). Note that for this verification we only simulated the effect of 4 micro-environments. We only simulated the full effect of the mutations on our simplified model as part of the model validation process.

### 2.4. Analysis of the dynamic behavior of our model

First, we obtained all the attractors using the exhaustive SAT-based search available as part of BoolNet that uses an adaption of the algorithm proposed by Dubrova and Teslenko (2011). The exhaustive SAT-based search formulates the attractor search as a boolean satisfiability (SAT) problem that is solved using the PicoSAT solver (Biere, 2008). Then, we classified the attractors based on extracellular micro–environment. After that, for each micro-environment, we inferred the EC behavior represented by each attractor. If all EC behaviors associated to one micro-environment where of the same kind, we added that micro-environment to the set of micro-environments that induce that EC behavior. If not all EC behaviors associated with one micro-environment where of the same kind, we added the micro-environment to the set of micro-environments that induce atypical EC behavior. Finally, we summarized the four sets of micro-environments by grouping them into disjoint subsets using their shared characteristics. To validate our model, we simulated all single gain and loss of function mutations. We then compared the simulated effect of each mutation with its experimentally observed effect as reported in the literature (when available). Biological organisms need to be resilient to mutations and fluctuations in the concentration or level of molecular activation, we refer to this property as robustness. For clarity, it is necessary to indicate which trait is robust, to which perturbation and a method to quantify the resilience to define a robust feature (Félix and Barkoulas, 2015). We measured three robust features: (1) The robustness of Phalanx, Stalk, and Tip EC behaviors to single gain and loss-of-function mutations. This was measured as the percentage of mutations that prevent the existence of any stable or cyclic patterns of molecular activation that correspond to said EC behavior. (2) The robustness of attractor determination to molecular activation noise. First, we generated a set of 1,000,000 aleatory initial states. For each initial state, we created a perturbed copy with a Hamming distance of one by reversing the activatory state of one random variable. We quantified attractor determination robustness to molecular activation noise, as the fraction of the initial states that reached the same attractor as their perturbed copies. (3) The robustness of the trajectories that lead to Phalanx, Stalk, and Tip EC behaviors to molecular activation noise. First, we generated a set of 1,000,000 aleatory initial states. For each initial state, we created a perturbed copy with a Hamming distance of one by reversing the activatory state of one random variable. We quantified the robustness of the EC behaviors to molecular activation noise, as the fraction of the initial states that reached an attractor that represents the same EC behavior as that of their perturbed copies. Additionally, we calculated the sensitivity of each component of the update rule to molecular activation noise. For each update rule component *f*_*i*_ ∈ *f*, we first generated a set of 500,000 aleatory initial states. For each initial state, we created a perturbed copy with a Hamming distance of one by reversing the activatory state of one random variable. Then we applied the update rule once to each initial state and to its perturbed copy. The fraction of initial states, where after update the activatory state of the variable *x*_*i*_(*t* + 1) is different for the initial state then it is for its perturbed copy is our estimation of the sensitivity for update rule *f*_*i*_.

## 3. Results

### 3.1. The network model

The model includes 143 nodes and 256 edges (Figure 2) the update rules of the network are included in Supplementary Section 2. To enable a more thorough analysis of the dynamic behavior of our model, we simplified our model and obtained a network composed of 64 nodes and 163 interactions, a diagram of our simplified model is shown in Figure 3. The update rules that define the dynamic behavior of our model are included as Equations 1–64. The EC behavior transitions integrated into both our detailed and simplified models are summarized in Figure 4A, and Supplementary Figures 1–14. Single gain- and loss-of-function mutations have a similar effect on the behaviors and transitions integrated into both models (Supplementary Figures 15, 16).

(1) $$\begin{array}{l} AKT\left(t+1\right)=PIP3\left(t\right) \end{array}$$

(2) $$\begin{array}{l} ALK1\left(t+1\right)=BMP9\left(t\right)\vee BMP10\left(t\right)\vee TGFB1\left(t\right) \end{array}$$

(3) $$\begin{array}{l} ALK5\left(t+1\right)=BMP9\left(t\right) \end{array}$$

(4) $$\begin{array}{l} AMPATP\left(t+1\right)=AMPATP\left(t\right) \end{array}$$

(5) $$\begin{array}{l} AMPK\left(t+1\right)=\left(AMPATP\left(t\right)\vee \left(\neg Oxygen\left(t\right)\right)\right)\\ \;\wedge \left(\neg AKT\left(t\right)\right) \end{array}$$

(6) $$\begin{array}{l} ANG1\left(t+1\right)=ANG1\left(t\right) \end{array}$$

(7) $$\begin{array}{l} ANG2(t+1)=(\neg KLF2(t))\wedge (HIF1(t)\vee ETS(t)\\ \;\vee AP1(t)\vee FOXO1(t)) \end{array}$$

(8) $$\begin{array}{l} AP1\left(t+1\right)=WNT5a\left(t\right) \end{array}$$

(9) $$\begin{array}{l} \beta catenin\left(t+1\right)=WNT5a\left(t\right)\vee WNT7a\left(t\right) \end{array}$$

(10) $$\begin{array}{l} BMP10\left(t+1\right)=BMP10\left(t\right) \end{array}$$

(11) $$\begin{array}{l} BMP9\left(t+1\right)=BMP9\left(t\right) \end{array}$$

(12) $$\begin{array}{l} Calcium\left(t+1\right)=PLCg\left(t\right)\vee ShearStress\left(t\right)\vee \left(\neg NO\left(t\right)\right) \end{array}$$

(13) $$\begin{array}{l} DLL4a\left(t+1\right)=ETS\left(t\right)\vee NICD\left(t\right) \end{array}$$

(14) $$\begin{array}{l} DLL4p\left(t+1\right)=DLL4p\left(t\right) \end{array}$$

(15) $$\begin{array}{l} ETS\left(t+1\right)=MEK\left(t\right)\vee VEGFR33\left(t\right) \end{array}$$

(16) $$\begin{array}{l} FAK\left(t+1\right)=SRC\left(t\right)\vee Integrin\left(t\right) \end{array}$$

(17) $$\begin{array}{l} FGF\left(t+1\right)=FGF\left(t\right) \end{array}$$

(18) $$\begin{array}{l} FOXO1\left(t+1\right)=\left(\neg AKT\right)\wedge SIRT1\left(t\right) \end{array}$$

(19) $$\begin{array}{l} HEY1(t+1)=NICD(t)\vee ((SMAD1(t)\vee SMAD2(t))\\ \;\wedge (\neg SIRT1(t))) \end{array}$$

(20) $$\begin{array}{l} HIF1\left(t+1\right)=\left(AMPK\left(t\right)\vee \neg TSC\left(t\right)\right)\wedge \neg Oxygen\left(t\right)\\ \;\wedge SIRT1\left(t\right) \end{array}$$

(21) $$\begin{array}{l} IGF\left(t+1\right)=IGF\left(t\right) \end{array}$$

(22) $$\begin{array}{l} Integrin\left(t+1\right)=ETS\left(t\right)\wedge \left(ShearStress\left(t\right)\vee TIE2\left(t\right)\right) \end{array}$$

(23) $$\begin{array}{l} JAGa\left(t+1\right)=SMAD1\left(t\right)\vee \beta catenin\left(t\right) \end{array}$$

(24) $$\begin{array}{l} JAGp\left(t+1\right)=JAGp\left(t\right) \end{array}$$

(25) $$\begin{array}{l} KLF2\left(t+1\right)=ShearStress\left(t\right) \end{array}$$

(26) $$\begin{array}{l} LEF1\left(t+1\right)=\beta catenin\left(t\right)\wedge \left(LEF1\left(t\right)\vee NRARP\left(t\right)\right) \end{array}$$

(27) $$\begin{array}{l} MEK(t+1)=(((PLCg(t)\wedge Calcium(t))\vee RAS(t))\\ \;\wedge (\neg AKT(t)))\vee FGF(t) \end{array}$$

(28) $$\begin{array}{l} NFAT\left(t+1\right)=Calcium\left(t\right) \end{array}$$

(29) $$\begin{array}{l} NICD\left(t+1\right)=\left(\neg NRARP\left(t\right)\right)\wedge NOTCH\left(t\right) \end{array}$$

(30) $$\begin{array}{l} NO\left(t+1\right)=Calcium\left(t\right)\vee AKT\left(t\right)\vee SIRT1\left(t\right) \end{array}$$

(31) $$\begin{array}{l} NOTCH\left(t+1\right)=\left(\neg JAGp\left(t\right)\right)\wedge ETS\left(t\right)\wedge DLL4p\left(t\right) \end{array}$$

(32) $$\begin{array}{l} NRP1\left(t+1\right)=\left(VEGFAxxx\left(t\right)\vee VEGFC\text{\_}Dp\left(t\right)\right)\\ \;\wedge \left(\neg NICD\left(t\right)\vee ETS\left(t\right)\right) \end{array}$$

(33) $$\begin{array}{l} NRARP\left(t+1\right)=NICD\left(t\right) \end{array}$$

(34) $$\begin{array}{l} Oxygen\left(t+1\right)=Oxygen\left(t\right) \end{array}$$

(35) $$\begin{array}{l} p38MAPK\left(t+1\right)=SRC\left(t\right)\vee PLCg\left(t\right)\vee ALK1\left(t\right) \end{array}$$

(36) $$\begin{array}{l} PECAM1\left(t+1\right)=VEGFR22\left(t\right)\vee VEGFR23\left(t\right)\\ \;\vee ShearStress\left(t\right)\vee VEcadherin\left(t\right) \end{array}$$

(37) $$\begin{array}{l} PIP3(t+1)=(\neg NICD(t))\wedge (\neg WNT5a(t))\\ \;\wedge (\neg WNT7a(t))\wedge (\neg NRP1(t))\\ \;\wedge (SRC(t)\vee KLF2(t)\\ \;\vee VEcadherin(t)\vee TIE2(t)) \end{array}$$

(38) $$\begin{array}{l} PLCg\left(t+1\right)=VEGFR22\left(t\right)\vee VEGFR33\left(t\right)\\ \;\vee WNT5a\left(t\right)\vee WNT7a\left(t\right) \end{array}$$

(39) $$\begin{array}{l} RAS\left(t+1\right)=PECAM1\left(t\right)\vee KLF2\left(t\right)\vee ALK1\left(t\right) \end{array}$$

(40) $$\begin{array}{l} ShearStress\left(t+1\right)=ShearStress\left(t\right) \end{array}$$

(41) $$\begin{array}{l} SIRT1\left(t+1\right)=AMPK\left(t\right)\wedge \left(HIF1\left(t\right)\vee FOXO1\left(t\right)\right) \end{array}$$

(42) $$\begin{array}{l} SMAD1\left(t+1\right)=\left(\neg SMAD6\left(t\right)\right)\wedge \left(\neg NRP1\left(t\right)\right)\wedge ALK1\left(t\right) \end{array}$$

(43) $$\begin{array}{l} SMAD2\left(t+1\right)=\left(\neg SMAD6\left(t\right)\right)\wedge \left(\neg NRP1\left(t\right)\right)\wedge ALK5\left(t\right) \end{array}$$

(44) $$\begin{array}{l} SMAD6\left(t+1\right)=NICD\left(t\right) \end{array}$$

(45) $$\begin{array}{l} SRC\left(t+1\right)=FAK\left(t\right)\vee ShearStress\left(t\right)\vee VEGFR22\left(t\right)\\ \;\vee VEGFR23\left(t\right) \end{array}$$

(46) $$\begin{array}{l} STAT3\left(t+1\right)=VEGFR22\left(t\right) \end{array}$$

(47) $$\begin{array}{l} TGFB1\left(t+1\right)=TGFB1\left(t\right) \end{array}$$

(48) $$\begin{array}{l} TIE2(t+1)=(\neg ANG2(t))\wedge ANG1(t)\wedge (ETS(t)\\ \;\vee KLF2(t)) \end{array}$$

(49) $$\begin{array}{l} TSC\left(t+1\right)=AMPK\left(t\right)\wedge \left(\neg AKT\left(t\right)\right) \end{array}$$

(50) $$\begin{array}{l} VEcadherin(t+1)=ETS(t)\wedge (SRC(t)\vee FAK(t)\\ \;\vee ShearStress(t)\vee HIF1(t)) \end{array}$$

(51) $$\begin{array}{l} VegfA\left(t+1\right)=\left(\neg Oxygen\left(t\right)\right)\vee HIF1\left(t\right)\vee STAT3\left(t\right)\\ \;\vee FOXO1\left(t\right)\vee NFAT\left(t\right)\vee KLF2\left(t\right) \end{array}$$

(52) $$\begin{array}{l} VEGFAxxx\left(t+1\right)=VEGFAxxxP\left(t\right)\vee VEGFAxxxA\left(t\right) \end{array}$$

(53) $$\begin{array}{l} VEGFAxxxA(t+1)=VegfA(t)\wedge IGF(t)\wedge ((\neg NICD(t)\\ \;\wedge \neg HIF1(t)\wedge \neg ETS(t))\\ \;\vee NFAT(t))\wedge (\neg AMPK(t)) \end{array}$$

(54) $$\begin{array}{l} VEGFAxxxd\left(t+1\right)=p38MAPK\left(t\right)\wedge VegfA\left(t\right) \end{array}$$

(55) $$\begin{array}{l} VEGFAxxxP\left(t+1\right)=VEGFAxxxP\left(t\right) \end{array}$$

(56) $$\begin{array}{l} VEGFC\text{\_}D\left(t+1\right)=VEGFC\text{\_}D\left(t\right) \end{array}$$

(57) $$\begin{array}{l} VEGFC\text{\_}Dp\left(t+1\right)=VEGFC\text{\_}Dp\left(t\right) \end{array}$$

(58) $$\begin{array}{l} Vegfr2\left(t+1\right)=\left(ETS\left(t\right)\wedge \left(\neg HEY1\left(t\right)\right)\right)\vee \neg Oxygen\left(t\right) \end{array}$$

(59) $$\begin{array}{l} VEGFR22(t+1)=Vegfr2(t)\wedge (PECAM1(t)\\ \;\vee ((VEGFC\_Dp(t)\vee VEGFAxxx(t))\\ \;\wedge \neg (VEGFAxxxd(t)\vee HIF1(t))) \end{array}$$

(60) $$\begin{array}{l} VEGFR23(t+1)=Vegfr2(t)\wedge Vegfr3(t)\wedge (PECAM1(t)\\ \;\vee \;VEGFAxxx(t)\vee VEGFC\_Dp(t)) \end{array}$$

(61) $$\begin{array}{l} Vegfr3\left(t+1\right)=NICD\left(t\right) \end{array}$$

(62) $$\begin{array}{l} VEGFR33(t+1)=Vegfr3(t)\wedge (PECAM1(t)\\ \;\vee VEGFC\_D(t)\vee VEGFC\_Dp(t)) \end{array}$$

(63) $$\begin{array}{l} WNT5a\left(t+1\right)=WNT5a\left(t\right) \end{array}$$

(64) $$\begin{array}{l} WNT7a\left(t+1\right)=WNT7a\left(t\right) \end{array}$$

**Figure 2:**
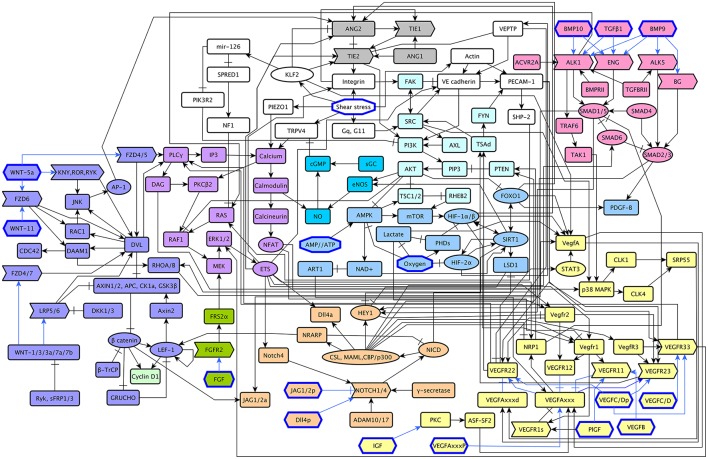
A diagram of our extended model: The *ANG/TIE* signaling pathway is shown in gray, *Shear Stress* in white, *Oxygen and Energy* in blue, *NO* in turquoise, *VEGF* in yellow, *AKT/SRC* in light blue, *TGF* in pink, *NOTCH* in orange, *WNT* in purple, *RAS/PLC*γ in violet, *CyclinD1* in light green, and *FGF* in green. Ligands are represented as hexagons, other micro-environment variables as octagons, receptors as right arrows, transcription factors as ellipses, and signal transducers as rounded rectangles. Intracellular signaling is represented in black arrows, extracellular signaling is represented with blue arrows. Activatory interactions are shown as regular arrows and inhibitory interactions are shown as blunt arrows.

**Figure 3:**
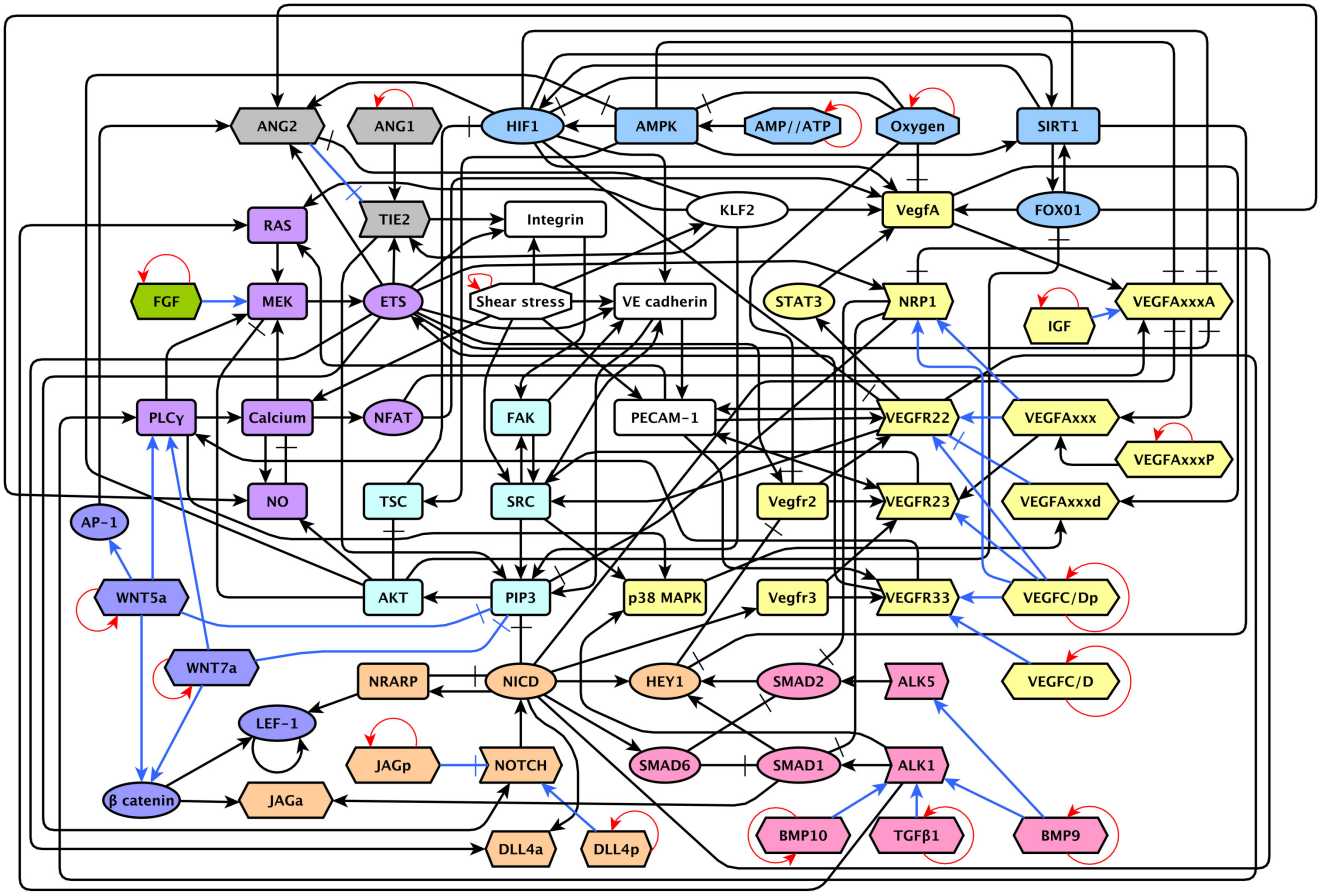
A diagram of our reduced network model: The *ANG/TIE* signaling pathway is shown in gray, *Shear Stress* in white, *Oxygen and Energy* in blue, *VEGF* in yellow, *AKT/SRC* in light blue, *TGF* in pink, *NOTCH* in orange, *WNT* in purple, *RAS/PLC*γ in violet, and *FGF* in green. Ligands are represented as hexagons, other micro-environment variables as octagons, receptors as right arrows, transcription factors as ellipses, and signal transducers as rounded rectangles. Intracellular signaling is represented in black arrows, extracellular signaling is represented with blue arrows. Activatory interactions are shown as regular arrows and inhibitory interactions are shown as blunt arrows. The self activatory feedback circuits required to keep the micro-environment constant during the simulation are shown in red.

**Figure 4:**
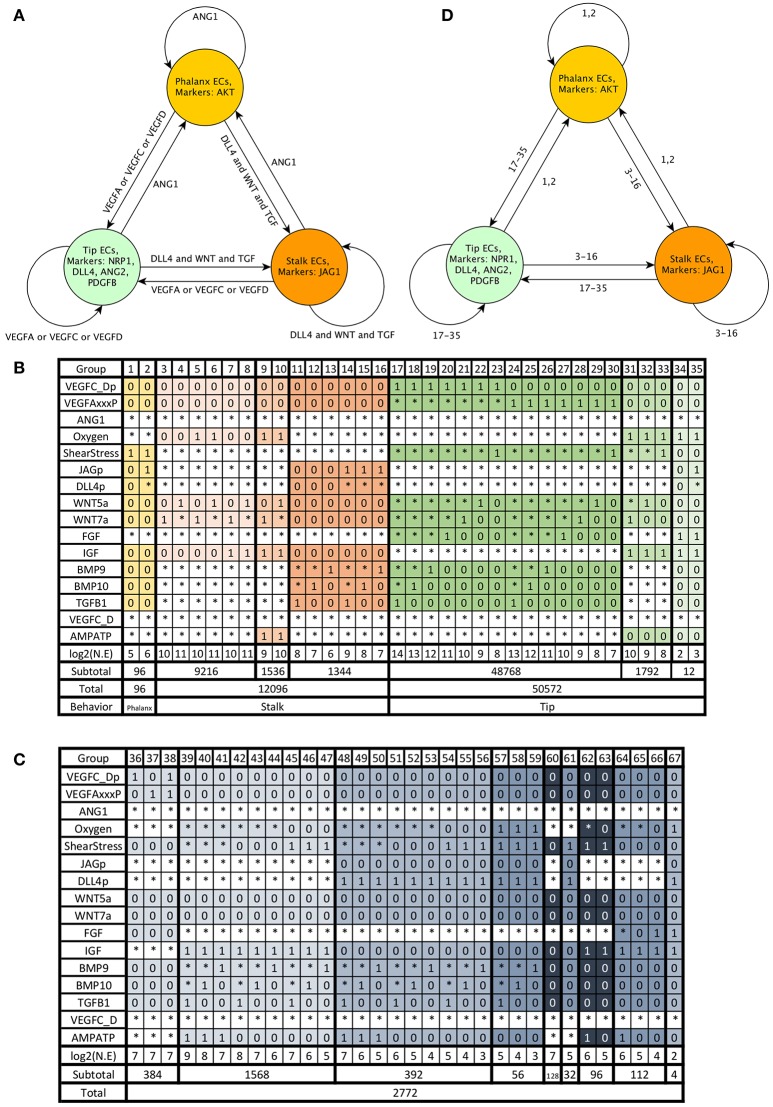
Endothelial cell behavior: Phalanx EC behavior shown in yellow, Stalk EC behavior shown in orange, Tip EC behavior shown in green, and other EC behavior is shown in gray: **(A)** Expected EC behavior in an extracellular micro-environment with normal oxygen concentration, ATP to ADP ratio and shear stress, **(B)** The extracellular micro-environments that cause Phalanx, Stalk, and Tip EC behavior according to the simulation of the dynamic behavior of our simplified model, **(C)** The extracellular micro-environments that cause other EC behavior, **(D)** Summary of EC behavior according to our model, the numbers on the edges represent the groups of micro-environments shown as columns in panel **(B)**.

### 3.2. The effect of the extracellular micro-environment on EC behavior

One of the main goals of this work is to understand how the concentration of several molecules in the extracellular micro-environment combines with the mechanical forces sensed by the mechano-receptors connected to the cytoskeleton of ECs controls EC behavior. We propose that the presence (1) or absence (0) of sufficient *VEGFC_Dp, VEGFAxxxP, ANG1, Oxygen, ShearStress, JAGp, DLL4p, WNT5a, WNT7a, FGF, IGF, BMP9, BMP10, TGFB1, VEGFC_D*, and *AMPATP* in the micro-environment of an EC determines its behavior. Further, we propose that Phalanx, Stalk, and Tip ECs retain the ability respond to the micro-environment in a similar manner and that explains the plasticity in EC behavior that has been experimentally observed (Blancas et al., 2012; Glaser et al., 2016). To investigate the effect of the extracellular micro-environment on EC behavior, we first found all the attractors that can be reached through the simulation of the dynamic behavior of our model. Then, we classified them according to their extracellular micro-environment. After that we interpreted the EC behavior represented by the attractors in each micro-environment. If all the attractors that correspond to a certain micro-environment represent the same kind of EC behavior, then we can state that the micro-environment causes that EC behavior. If most micro-environments cause either Tip, Stalk, or Phalanx EC behavior, then to a large extent the extracellular micro-environment controls EC behavior.

Notably, there are 2^16^ = 65536 possible micro-environments. From these, according to our model, under wild-type conditions 50,572 (77.16675%) micro-environments cause Tip EC behavior, 12,096 (18.45703%) cause Stalk EC behavior, and 96 (0.1464844%) cause Phalanx EC behavior. The characteristics of the groups of micro-environments that lead to Phalanx, Stalk, and Tip EC behavior are summarized in Figure 4B and Table 1. The intracellular molecules that are active or inactive in all the patterns of molecular activation in each group are also summarized in Table 1. The other 2,772 micro-environments (4.229736%) cause atypical dynamical patterns, including attractors that cycle between the Tip, Stalk, and/or Phalanx EC behaviors. This means that 62,764 (95.770374%) of the micro-environments induce a certain EC behavior regardless of the internal pattern of molecular activation (Figure 4D). Therefore, according to our model, in most cases, the extracellular micro-environment controls EC behavior.

**Table 1:** Phalanx, Stalk, and Tip EC behavior: The groups correspond to those in Figure 4B, active molecules shown in blue, inactive molecules shown in red.

**Behavior (groups, micro–environments, attractors)**	**Micro–environment characteristics**	**Molecular activity inside the cell**
Phalanx (1–2, 96, 96)	ShearStress, and VEGFC_Dp, VEGFAxxxP, WNT5a, WNT7a, IGF, BMP9, BMP10, TGFB1, and (JAGp or DLL4p)	RAS, KLF2, VegfA, Calcium, NFAT, FAK, PECAM1, NO, SRC, VEGFAxxxd, AKT, PIP3, p38MAPK, and ANG2, HIF1, AMPK, SIRT1, FOXO1, NRP1, VEGFAxxxA, VEGFAxxx, TSC, VEGFR23, AP1, Vegfr3, VEGFR33, catenin, LEF1, NRARP, NICD, HEY1, SMAD2, ALK5, JAGa, NOTCH, SMAD6, SMAD1, ALK1 do not divide or recruit mural cells.
Stalk I (3–8, 9216, 58896)	VEGFC_Dp and VEGFAxxxP and (WNT5a or WNT7a), and (Oxygen or IGF)	VegfA, MEK, ETS, PLCg, Calcium, NFAT, NO, VEGFAxxd, p38MAPK, βcatenin, JAGa, DLL4a, NRP1, VEGFAxxxA, VEGFAxxx, AKT, PIP3
Stalk II (9–10, 1536, 14688)	Oxygen and IGF and AMPATP and VEGFC_Dp and VEGFAxxxP and (WNT5a or WNT7a)	AMPK, Oxygen, VegfA, AMPATP, MEK, ETS, IGF, PLCg, Calcium, NFAT, NO, TSC, VEGFAxxd, p38MAPK, βcatenin, JAGa, DLL4a NRP1, VEGFAxxxA, VEGFAxxx, AKT, PIP3
Stalk III (11–16, 1344, 3276)	VEGFC_Dp and VEGFAxxxP and WNT5a WNT7a and IGF and (JAGp or DLL4p) and (BMP9 or BMP10 or TGF1)	RAS, p38MAPK, JAGa, SMAD1, ALK1, HIF1, NRP1, IGF, VEGFAxxxA, VEGFAxxx, VEGFR23, βcatenin, LEF1, NICD, NOTCH, SMAD6 do not divide
Tip I (17–30, 48768, 244680)	(VEGFC Dp, or VEGFAxxxP) and (ShearStress or WNT5a or WNT7a or FGF or BMP9 or BMP10 or TGFB1)	VegfA, MEK, ETS, NRP1, VEGFAxxxd, p38MAPK, DLL4a, AKT, PIP3, SMAD1, SMAD2
Tip II (31–33, 1792, 4096)	VEGFAxxxP and VEGFC Dp and AMPATP and IGF and Oxygen and (ShearStress or WNT5a or WNT7a)	Oxygen, VegfA, MEK, ETS, NRP1, Calcium, NFAT, VEGFAxxxA, VEGFAxxx, NO, VEGFAxxxd, p38MAPK, DLL4a, HIF1, AMPK, SIRT1, FOXO1, TSC, AKT, PIP3, SMAD1, SMAD2 do not recruit mural cells
Tip III (34–35, 12, 12)	VEGFAxxxP and VEGFC Dp and AMPATP and IGF and Oxygen and ShearStress and WNT5a and BMP9 and BMP10 and TGF1 and WNT7a and (JAGp or DLL4p)	ANG2,Oxygen, RAS, VegfA, FGF, MEK, ETS, VEcadherin, STAT3, NRP1, PLCg, Calcium, NFAT, FAK, PECAM1, VEGFR22, VEGFAxxxA, VEGFAxxx, NO, SRC, Vegfr2, VEGFAxxxd, p38MAPK, DLL4a, TIE2, HIF1, AMPK, SIRT1, Integrin, KLF2, FOXO1, TSC, VEGFR23, AP1, AKT, PIP3, Vegfr3, VEGFR33, βcatenin, LEF1, NRARP, NICD, HEY1, SMAD2, ALK5, JAGa, NOTCH, SMAD6, SMAD1, ALK1 do not divide and do not recruit mural cells

*βcatenin and LEF1 activity is required to allow Cyclin D1–mediated activation of the cell cycle. FOXO1 or SMAD2 activity is required for PDGFβ–mediated mural cell recruitment*.

*Tip ECs* are localized at the leading edge of vessel sprouts forming numerous long dynamic filipodia. Additionally, Tip cells migrate toward angiogenic stimuli, do not contribute to lumen formation, and seldom divide. Tip ECs are characterized by expressing high levels of DLL4, CXCR4, ANG2, PDGFB, receptors for axon guidance cues, such as the Netrin receptor UNC5B, APLN, various proteases like uPAR and NRP1, (del Toro et al., 2010; Blancas et al., 2012). We use NRP1 activity as a Tip EC-specific marker, and also require DLL4 expression, because DLL4 directly inhibits neighboring cells from becoming Tip ECs. Additionally, AKT must be inactive in Tip ECs. It is known that an increase above a certain threshold on the concentration of VEGFA or proteolytically processed VEGFC or D in the micro-environment surrounding an EC triggers Tip EC behavior (sections 1.2 and 1.10 in the Supplementary Material). According to the simulated dynamic behavior of our model, the micro-environments that include VEGFAxxxP or VEGFC_Dp and induce Tip EC behavior, also include either *ShearStress, WNT5a, WNT7a, FGF, BMP9, BMP10*, or *TGFB1*. Alternatively, the model also allows for the possibility that two groups of micro-environments that lack paracrine VEGF activity may cause Tip EC behavior, achieved by inducing autocrine VEGFA activity.

*Stalk ECs* trail Tip ECs, proliferate rapidly and contribute to lumen formation. While *TIE2* is constitutively expressed in all ECs, its protein is detectable by antibody staining on Stalk ECs but not on Tip ECs. Stalk cells also express the Apelin receptor *APJ* and *JAG1* (del Toro et al., 2010; Blancas et al., 2012). We use autocrine JAG1 as a Stalk EC marker due to the specificity of its expression and its function, which is to suppress Notch signaling in neighboring Tip ECs, further, Stalk ECs, are characterized by their lack of NRP1 activity. A sufficient concentration of WNT, TGF and NOTCH ligands, as well as an absence of VEGF in the extracellular micro-environment of an EC, is known to cause Stalk EC behavior (section 1.10 in the Supplementary Material). According to the simulated dynamic behavior of our model, it is possible to obtain the Stalk EC behavior in a micro-environment that complies with either of the following three lists of requirements: (a) WNT activity, lack of VEGF activity, and low Oxygen or IGF; (b) WNT activity, no VEGF activity, Oxygen, IGF, and sufficient energy; and (c) lack of VEGF, NOTCH, WNT, and IGF ligands that includes one of the TGF ligands.

*Phalanx ECs* form strong EC–EC bonds to compose the tunica intima in stable blood vessels. The Pericytes and SMCs that cover stable blood vessels secrete ANG1 to maintain the integrity of the layer of Phalanx ECs. Phalanx ECs are characterized by a high level of *VEGFR1 (FLT1)* and *TIE1* expression (Blancas et al., 2012), even though neither is a Phalanx EC specific marker. We use active AKT (Kerr et al., 2016) as well as inactive NRP1 and JAGa as specific Phalanx EC markers. Changes in the extracellular concentration of VEGFs, a decrease in the availability of oxygen or energy within the cell, and shear stress cause ANG2-mediated activation of the ECs that line blood vessels (section 1.8 in the Supplementary Material). According to our model, the lack of *VEGF, NOTCH, WNT* and *TGF* pathway activity is necessary to observe a stable Phalanx EC behavior. The simulated Phalanx ECs do not divide and do not recruit mural cells.

#### 3.2.1. Atypical EC behavior

We performed with our model a systematic study of the dynamical behavior of a regulatory network under all possible combination of the micro-environments. Apart from the clearly identifiable phenotypes mentioned in the previous paragraphs, we observed some atypical responses. If the attractors that correspond to a certain micro-environment represented different EC behaviors, or any of the attractors represented an EC behavior that was different from Tip, Stalk, or Phalanx EC behavior, we considered that the micro-environment causes atypical EC behavior. For completeness, we describe such atypical behaviors in Table 2.

**Table 2:** Atypical EC behavior: The groups correspond to those in Figure 4C, active molecules shown in blue, inactive molecules shown in red.

**Behavior (groups, micro–environments, attractors)**	**Micro–environment characteristics**	**Molecular activity inside the cell**
Atypical I (36–38, 384, 3920)	(VEGFAxxxP or VEGFC Dp) and ShearStress, and WNT5a, and WNT7a, and FGF, and BMP9, and BMP10, and TGFB1	KLF2, FGF, AP1, βcatenin, LEF1, SMAD2, JAGa, SMAD1,ALK1
Atypical II (39–47, 1568, 11172)	IGF and VEGFC_Dp and VEGFAxxxP and WNT5a and WNT7a and (AMPATP or Oxygen or ShearStress) and (BMP9 or BMP10 or TGFB1)	RAS, p38MAPK, ALK1, HIF1, AP1, βcatenin, LEF1
Atypical III (48–56, 392, 1876)	VEGFC_Dp and VEGFAxxxP and WNT5a and WNT7a and JAGp and IGF and DLL4p and (AMPATP or Oxygen or ShearStress) and (BMP9 or BMP10 or TGFB1)	RAS, p38MAPK, ALK1, HIF1, NRP1, VEGFAxxxA, VEGFAxxx, AP1, βcatenin and LEF1
Atypical IV (57–59, 56, 112)	VEGFC_Dp and VEGFAxxxP and WNT5a and WNT7a and JAGp and IGF and AMPATP and Oxygen and ShearStress and DLL4p and (BMP9 or BMP10 or TGFB1)	RAS, KLF2, VegfA, Calcium, NFAT, FAK, PECAM1, NO, SRC, VEGFAxxxd, p38MAPK, ALK1, ANG2, HIF1, SIRT1, FOXO1, NRP1, VEGFAxxxA, VEGFAxxx, TSC, AP1, βcatenin, LEF1
Atypical V (60, 128, 687)	VEGFC_Dp and VEGFAxxxP and ShearStress and WNT5a and WNT7a and IGF and BMP9 and BMP10 and TGFB1	KLF2, NRP1, VEGFAxxxA, VEGFAxxx, AP1, βcatenin, LEF1, SMAD2, ALK5, JAGa, ALK1
Atypical VI (61, 32, 64)	ShearStress and DLL4p and VEGFC_Dp and VEGFAxxxP and WNT5a and WNT7a and JAGp and IGF and BMP9 and BMP10 and TGFB1	RAS, KLF2, VegfA, Calcium, NFAT, FAK, PECAM1, NO, SRC, VEGFAxxxd, p38MAPK, ANG2, HIF1, SIRT1, FOXO1, NRP1, VEGFAxxxA, VEGFAxxx, AP1, βcatenin, LEF1, SMAD2, ALK5, JAGa, SMAD1, ALK1 do not recruit mural cells
Atypical VII (62–63, 96, 456)	ShearStress and IGF and VEGFC_Dp and VEGFAxxxP and WNT5a and WNT7a and BMP9 and BMP10 and TGFB1 and (AMPATP or Oxygen)	RAS, KLF2, VegfA, Calcium, NFAT, FAK, PECAM1, NO, SRC, VEGFAxxxd, p38MAPK, ANG2, HIF1, SIRT1, FOXO1, AP1, βcatenin, LEF1, SMAD2, ALK5, SMAD1, ALK1 do not recruit mural cells
Atypical VIII (64–66, 112, 1358)	IGF and VEGFC_Dp and VEGFAxxxP and ShearStress and WNT5a and WNT7a and BMP9 and BMP10 and TGF and (AMPATP or FGF or Oxygen)	KLF2, AP1, βcatenin, LEF1, SMAD2, ALK5, SMAD1 , ALK1
Atypical IX (67, 4, 8)	VEGFC_Dp and VEGFAxxxP and ShearStress and JAGp and WNT5a and WNT7a and BMP9 and BMP10 and TGF and AMPATP and Oxygen and DLL4p and FGF and IGF	ANG2, MEK, ETS, NOTCH, DLL4a, TIE2, HIF1, AMPK, SIRT1, Integrin, KLF2, FOXO1, TSC, AP1, βcatenin, LEF1, SMAD2, ALK5, JAGa, SMAD1, ALK1, DLL4a do not recruit mural cells

*All the atypical EC behaviors include a quiescent cell cycle because βcatenin and LEF1 activity is required to allow Cyclin D1–mediated activation of the cell cycle. FOXO1 or SMAD2 activity is required for PDGFβ–mediated mural cell recruitment*.

#### 3.2.2. EC proliferation

EC proliferation allows the number of ECs to increase during sprout elongation. We describe the effect of the micro-environment on EC proliferation according to the simulated dynamic behavior of our model in Table 3. Note that in accordance with what has been reported in the literature, only Tip and Stalk ECs proliferate.

**Table 3:** EC proliferation: Cyclin D1–mediated activation of the cell cycle requires βcatenin and LEF1 activity. Active molecules shown in blue, inactive molecules shown in red.

**Behavior (micro–environments, attractors)**	**Micro–environment characteristics**	**Molecular activity inside the cell**
All divide (12288, 42432)	JAGp and DLL4p and (WNT5a or WNT7a)	VegfA, MEK, ETS, PLCg, Calcium, NFAT, NO, VEGFAxxd, p38MAPK, βcatenin, LEF1, JAGa, NOTCH, DLL4a, AKT, PIP3
Some divide (36864, 244656)	(JAGp or DLL4p) and (WNT5a or WNT7a)	VegfA, MEK, ETS, PLCg, Calcium, NFAT, NO, VEGFAxxd, p38MAPK, βcatenin, JAGa, DLL4a, VEGFR23, AKT, PIP3, Vegfr3, VEGFR33, NRARP, NICD, NOTCH, SMAD6
None divide (16384, 58309)	WNT5a and WNT7a	βcatenin, LEF1, AP1

### 3.3. Model validation

Certain diseases exhibit abnormal angiogenesis, because the affected tissue or organ secretes abnormal amounts of angiogenic signals. Simulating the effect of a pathological extracellular micro-environment on EC behavior can be used to understand how a disease is causing abnormal vascular remodeling, the insights are only valid if the dynamic behavior of the model can reproduce the relevant experimental observations. If an experimental observation includes a sufficiently well-defined extracellular micro-environment and an observed EC behavior. Then the extracellular micro-environment fits only one column in Figure 4B or Figure 4C. If the EC behavior according to our model (shown at the bottom row of the column that corresponds to the micro-environment) is the same as the observed EC behavior, then our model fits that experimental observation.

#### 3.3.1. Tumor angiogenesis

The micro-environment inside many tumors is hypoxic, containing a high concentration of VEGFA and FGF. This state causes the formation of many leaky blood vessels (Nussenbaum and Herman, 2010). Our model can describe this state, as shown in Figure 4B group 27. The results indicate that the mentioned micro-environment induces Tip EC behavior, and inhibits Phalanx EC behavior, which is consistent with experimental observations.

#### 3.3.2. Pathological ocular angiogenesis

Diabetic retinopathy, age-related macular degeneration, retinopathy of prematurity, and other irreversible causes of blindness involve pathological angiogenesis. The capillaries of the retina are unique, the inner layer of the blood-retinal barrier is like that of other capillaries, and is composed of a single layer of ECs. However, the outer layer of the blood-retinal barrier is formed by retinal pigment epithelial cells instead of pericytes and SMCs. Pathological ocular angiogenesis is triggered by hypoxia from neuronal metabolism, inflammatory signals, and oxidative stress. Those micro-environmental conditions cause retinal pigmented epithelium, astrocytes, Müller cells, ECs, ganglion cells to secrete VEGFA (Siemerink et al., 2010). According to our model, the Tip ECs that secrete VEGFA during pathological ocular angiogenesis are likely exposed the extracellular micro-environments in groups 34–35 in Figure 4B, and are affected by oxidative stress, lack of shear stress and have sufficient oxygen. The other Tip ECs involved in pathological ocular angiogenesis and induced by paracrine VEGFA correspond to groups 17–30 in Figure 4B.

Other angiogenic pathologies are caused by mutations that affect how an EC responds to changes in the extracellular micro-environment. We used our simplified model to simulate the effect of all single gain- and loss-of-function mutations on EC behavior. Specifically, we analyzed how each mutation affects the groups of extracellular micro-environments that cause Tip, Stalk, and Phalanx EC behaviors in our simplified model. The effect of some of the mutations has been observed experimentally and it should be possible to simulate the observed behavior using our model. The expected effect of reducing, or enlarging the number of extracellular micro-environments that cause each EC behavior depends on the likelihood of appearance of each micro-environment. Only when almost all or none of the micro-environments lead to a certain EC behavior, and the mutation has been observed *in–vitro* or *in–vivo* it is possible to compare the simulated effect of a certain mutation (Supplementary Table 13) with its experimentally observed effect.

Simulated loss of autocrine function of *DLL4, ETS, MEK*, or *NRP1*, leads to the loss of functional Tip EC behavior, strongly favoring Stalk EC behavior. Importantly, all four mutations have been observed to cause severe vascular defects *in vivo* and *in vitro* (Supplementary Tables 6, 10, and 12). The loss of autocrine DLL4 leads to the formation of a higher number of Tip ECs that do not inhibit their neighbor ECs from becoming Tip ECs (del Toro et al., 2010).

Simulated gain-of-function mutations for proteolytically active VEGFA, VEGFC, and VEGFD as well as NRP1, prevent Stalk EC behavior and cause more than 99% of the extracellular micro-environments to induce Tip EC behavior. *In vivo* and *in vitro*, proteolytically active VEGFA, VEGFC, and VEGFD increase blood vessel branching, angiogenesis, and permeability (Supplementary Tables 11, 12).

Simulations indicate that the Phalanx EC behavior is prevented by a loss of AKT, PIP3, or ShearStress function, or alternatively by constitutive ALK1, βcatenin, BMP10, BMP9, IGF, autocrine JAG, NICD, NOTCH, NRP1, SMAD1, TGFβ1, proteolytically active VEGFA, VEGFC, or VEGFD, WNT5a, or WNT7a activity. *In vitro* and *in vivo*, loss of AKT, PIP3, or ShearStress leads to mural cell loss, blood vessel destabilization and regression (Supplementary Tables 2, 3). Constitutive βcatenin, IGF, NOTCH, NRP1, SMAD1, proteolytically active VEGFA, VEGFC, or VEGFD, WNT5a, or WNT7a activity induces EC migration, proliferation, survival, or angiogenesis (Supplementary Tables 4, 8–12).

### 3.4. Robustness analysis

Molecular regulatory networks must balance the need to ignore noise perturbations with the need to respond adequately to stimuli. A Boolean network can be classified as ordered, critical, or chaotic. Ordered Boolean networks resist most perturbations without any important changes in their dynamic behavior and are not sufficiently sensitive to stimuli. Chaotic Boolean networks tend to magnify perturbations and do not resist enough noise. Critical Boolean networks are selectively sensitive to certain perturbations and are sufficiently resilient to noise to be adequate models of molecular regulatory networks (Lloyd-Price et al., 2012). Additionally, the robustness of each trait has specific implications.

#### 3.4.1. The robustness of Tip, Stalk, and Phalanx EC behavior to single gain and loss-of-function mutations

The resilience of a functional phenotype to changes in the genotype allows the accumulation of genetic variation in a population, and needs to be achieved without limiting excessively the ability of a species to adapt by evolving different traits (Kirschner and Gerhart, 1998; Jiménez et al., 2015). The simulations showed that 23/128 = 17.96875% of all single gain- and loss-of-function mutations did not affect EC behavior at all. Furthermore, 82/128 = 64.0625% of mutations only cause changes in the response of an EC to certain extracellular micro-environments. The other 23/128 = 17.96875% of the mutations led to the loss of an EC behavior. Then, 4/128 = 3.125% of all mutations cause loss of Tip EC behavior. The same number of mutations cause Stalk EC behavior loss and strongly favor Tip EC behavior. Finally, 18/128 = 14.0625% of the mutations cause loss of Phalanx EC behavior. This set of results imply that our model of the network is robust to the complete loss of any of the main EC behaviors, however many mutations change the number of micro–environments that cause Tip, Stalk, and Phalanx EC behaviors (Supplementary Tables 13, 14).

#### 3.4.2. The robustness of attractor determination and EC behavior to molecular activation noise

Only 33.0538% of the trajectories followed by the perturbed copies of 1,000,000 random initial states reached the same attractor as the original state. In contrast, when we used 1,000,000 random initial states to test the robustness of EC behavior to molecular activation noise in 98.90088% of the relevant experiments the perturbation did not affect Tip EC, in 95.30536% of the relevant experiments, the perturbation did not affect Stalk EC behavior, and in 86.58824% of the relevant experiments, the perturbation did not affect Phalanx EC behavior. In general, 97.91060% of the random initial states reached the same EC behavior as the one reached by their perturbed copies.

#### 3.4.3. The sensitivity of each component of the update rule to molecular activation noise

**Figure 5:**
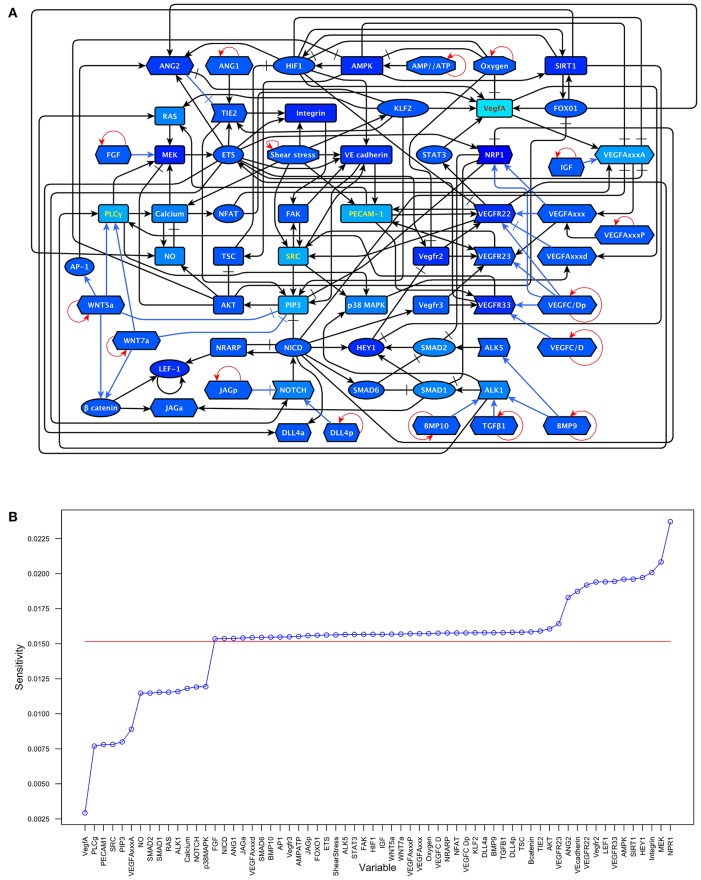
Update rule component sensitivity: **(A)** A darker shade of blue indicates a higher sensitivity in the update rule. Values range from VegfA = 0.002926 to NRP1 = 0.023716. **(B)** The sensitivities of the components of the update rule arranged from smallest to largest compared to the average sensitivity (0.01515947) which is shown as a red line.

To understand which variables are more sensitive to stimuli and which ones tend to be more resilient to molecular activation noise. We estimated the sensitivity of each component of the update rule as described in the methods section. The results are shown in Figure 5 and the sensitivity values in section 2.1 in the Supplementary Material. The nodes with the six most sensitive update rules in our network are NRP1, MEK, Integrin, HEY1, SIRT1, AMPK. Even the update rule of NRP, the most sensitive in our model, has a relatively low sensitivity of 0.023716.

## 4. Discussion

We presented in this work a reconstruction of the regulatory network involved in the control of angiogenesis, integrating the largest set of canonical signaling pathways to date. The dynamical behavior of the network, simulated as a Boolean network model, recovered the qualitative patterns of molecular activation observed in Phalanx, Tip, and Stalk ECs. Furthermore, the simulated behavior of the model corresponded to what has been reported in the literature regarding the high degree of behavioral plasticity between Phalanx, Stalk, and Tip EC behaviors in response to specific molecular micro-environments. Moreover, the model was also able to describe the effect of gain- and loss-of-function mutations.

### 4.1. Insights and predictions based on the simulated dynamic behavior of our model

The qualitative agreement between our model and published data shows that the model is a useful framework to understand the mechanisms that underly normal angiogenesis. Furthermore, it allows generating hypotheses on the mechanisms by which a disruption in the system might lead to deviation in EC behavior, which might eventually lead to a pathogenic phenotype. The qualitative agreement between our model and published results cannot be attributed to some sort of model fitting. This is evidenced by the high robustness observed in the model against the complete loss of any of the main EC behaviors (Supplementary Tables 13, 14), despite the perturbations introduced in the update rules. Nonetheless, when we analyzed the effect of single gain- and loss-of-function mutations, the simulations recovered the observed effects of such mutations under certain micro-environments.

Our micro-environment EC behavior map allows us to put forward the following hypotheses about the requirements for Tip Stalk and Phalanx EC behaviors: (1) In a micro-environment with an active, paracrine VEGF ligand, the presence of either *ShearStress, WNT5a, WNT7a, FGF, BMP9, BMP10*, or *TGFB1* is necessary to induce Tip EC behavior. (2) A micro-environment without VEGF can induce Tip EC behavior if it includes Oxygen, nutrients and IGF (Tip II, and Tip III in Table 1). However, the resulting Tip ECs secrete autocrine VEGFA. (3) DLL4 is not required for a micro-environment to induce Stalk EC behavior. (4) Shear stress and the absence of VEGF, TGF, IGF, WNT, and NOTCH ligands in the micro-environment is needed to observe a stable Phalanx EC behavior.

Based on the simulated effect of constitutive NRP1 activity, we predict that it prevents Stalk EC behavior and induces Tip EC behavior. We predict that constitutive ALK1, BMP9, BMP10, autocrine JAG, NICD, NRP1, SMAD1, and TGFβ1 activity inhibits Phalanx EC behavior based on the simulated effect of the corresponding gain-of-function mutations. Therefore, the model helps predict which mutations cause augmented mural cell loss, EC migration, proliferation, and angiogenesis, concomitant with inhibited Phalanx EC behavior.

Knowing the response of endothelial cells under a specific micro-environment is extremely relevant because inhibiting angiogenesis is an important medical goal during the treatment of vascular retinal disorders and cancer. Most of the drugs that are used to inhibit angiogenesis target the VEGF signaling pathway, inhibiting Tip EC behavior (Yadav et al., 2015). Our model suggests alternative ways to eliminate Tip EC behavior. Specifically, by eliminating the function of DLL4, ETS, MEK, or NRP1. Notably, both NRP1 and DLL4 are located on the cell membrane of ECs and are therefore easily reachable by drugs. Furthermore, in vascular retinal disorders, vascular permeability increases and vascular integrity diminishes, that is associated with intra-ocular hemorrhage and invasive potential of cancer. In principle, an extracellular micro-environment conducive to Phalanx EC behavior would help increase vascular integrity. Finally, stimulating angiogenesis is also an important medical goal during wound healing. It would be possible, thus, to use our model to explore one of the micro-environments that lead to Tip EC behavior and therefore, induce the wound healing process.

Arteriovenous malformations are very frequent in patients who suffer from Hereditary Hemorrhagic Telangiectasia (HHT), a disease associated with reduced ALK1, ENG, or SMAD4 function. In addition, Pulmonary Arterial Hypertension (PAH) is associated with reduced BMPRII or SMAD1 function. Furthermore, venous malformations have been observed in mice with constitutive TIE2 activity, as well as in mice with loss of ERK function. According to our model, the simulated effect of the mutations mentioned above includes an increase in the number of micro-environments that lead to Phalanx EC behavior, suggesting that the mentioned diseases are a consequence of ectopic blood vessel stabilization.

### 4.2. Assumptions and limitations of our model

In this first version of the model of angiogenesis, we focus on the effect of the extracellular micro-environment on the behavior of a single endothelial cell. By using a Boolean model, we assume that all variables can only be active or inactive. Further, we use a synchronous update approach, therefore, we assume that all variables are activated or inhibited simultaneously. The limitations of our model affect the number of sprouting angiogenesis processes that we can reproduce and the extent to which we can simulate them. Some of the processes that are beyond the scope of our model have been studied using other previously published models (Peirce, 2008; Qutub et al., 2009; Scianna et al., 2013; Logsdon et al., 2014; Heck et al., 2015; Qutub and Popel, 2015) while other processes offer opportunities for further research as specified in the following paragraphs.

#### 4.2.1. Secretion of angiogenic factors

According to our model, certain conditions cause ECs to secrete vascular growth factors (Figure 4B columns 31–35), the conditions that cause ECs to secrete active VEGFA (VEGFAxxxA) include sufficient oxygen, IGF, and a low AMP to ATP ratio. Normally, ECs are in contact with blood preventing hypoxia and lack of nutrients. The cells that compose other tissues respond to hypoxia or a high AMP to ATP ratio by secreting angiogenic factors; however, those cells are not included in our model. Additionally, Oxygen and then the secreted VEGF form concentration gradients. A continuous model that includes the geometry of the region or organ of interest as a boundary condition is necessary to simulate the gradient. Moreover, VEGFR1s secretion modulates the VEGFA concentration gradient (Chappell et al., 2016).

#### 4.2.2. Vessel destabilization

ANG2 activity is associated with mural cell detachment and it is possible to reproduce EC behavior during blood vessel destabilization using our model. However, it is not possible to reproduce pericyte and smooth muscle cell detachment because they are not included in our model. Some previous modeling efforts have included blood vessel destabilization (Zheng et al., 2013). However, in our opinion, mural cell behavior during angiogenesis merits a more detailed exploration.

#### 4.2.3. Tip and Stalk cell differentiation

We carefully analyzed tip and stalk EC differentiation using our model emphasizing the interaction between the VEGF, WNT, TGF, NOTCH, Calcium, and NO signaling pathways during Tip and Stalk behavior specification. It is noteworthy that while Tip cells induce Stalk behavior in their neighbors by expressing DLL4 (Blanco and Gerhardt, 2013), according to our model NOTCH signaling inhibits Tip EC behavior only in a small group of micro-environments (Figure 4B, columns 34 and 35). A possible explanation for this apparent discrepancy is that active NOTCH signaling induces the secretion of VEGFR1s, which binds VEGFA, effectively raising the extracellular concentration of VEGFA needed to induce Tip EC behavior in the cells with active NOTCH signaling. In our Boolean model, it is not possible to include the changing VEGFAxxxP threshold, this would require a continuous model. Further, at the multicellular level, the chronological order in which ECs are affected by VEGFA and DLL4-mediated lateral inhibition creates a race condition (Bentley and Chakravartula, 2017). The temporal modulation of Tip and stalk EC behavior, including the effect of filipodia on tip cell sensitivity to VEGF, has been explored by previous modeling efforts (Venkatraman et al., 2016). A continuous, asynchronous, multicellular model that includes Matrix metalloproteinase, Apelin signaling (Palm et al., 2016) and VEGFR1s secretion (Chappell et al., 2016) would offer additional valuable insights.

#### 4.2.4. Sprout elongation

We simulated the micro-environmental conditions that may cause ECs to divide. However, our model does not include cell shape, which also changes during sprout elongation. Further sprout elongation is a multicellular process and our model includes only one EC. Several previous modeling efforts have studied sprout elongation (Logsdon et al., 2014). The authors of Norton and Popel (2016) analyzed the effect of EC proliferation, elongation, and migration during sprout elongation. Mechanical forces regulate both the location of sprout initiation and the rate of sprout elongation (Ghaffari et al., 2015), included in the model proposed by the authors of Vavourakis et al. (2017). A multi-scale model including cytoskeletal dynamics, molecular activation, and mechanical forces would greatly enhance our understanding of sprout elongation.

#### 4.2.5. Lumen formation and expansion

PIP3, FAK, and SRC activity has been associated with vacuole secretion that is one of the main processes involved in lumen formation. According to the simulated dynamic behavior of our model, all Phalanx cells secrete vacuoles, additionally, type III Stalk ECs may also secrete vacuoles. Lumen formation is a multicellular process, that involves vacuole secretion and cytoskeletal remodeling. Simulating lumen formation, EC repulsion and flow-mediated lumen formation is beyond the scope of our current model. The authors of Boas and Merks (2014) focused their modeling efforts on the study of lumen formation.

#### 4.2.6. Anastomosis

Is a multicellular process that involves cytoskeletal remodeling including specific shape changes that are beyond the scope of our model. Anastomosis has been included in several 2D and 3D models (Zheng et al., 2013; Norton and Popel, 2016). ECs with a reduced concentration of membrane-localized VEGFR1 are more likely to form stable connections with incoming sprouts (Nesmith et al., 2017). A multicellular model that integrates VEGFR1 regulation, and how it affects anastomosis, may help explain micro–vascular architecture.

#### 4.2.7. Vessel stabilization

Phalanx EC behavior is expected in stable blood vessels and is recovered by our model. PDGFB-mediated mural cell recruitment is also recovered by our model. Other multicellular effects of vessel stabilization, such as decreased blood vessel permeability, are beyond the scope of our model. Some previous modeling efforts have included blood vessel stabilization (Zheng et al., 2013). However, in our opinion, mural cell behavior during angiogenesis merits a more detailed exploration.

#### 4.2.8. Pruning

Some of the micro-environments that cause atypical EC behavior without VEGF, FGF, IGF, and without Shear Stress (Figure 4C, group 60) may correspond to EC behavior during pruning. However, pruning involves changes in EC shape, EC fusion events, and EC migration, which have not been included in our model. Pruning is mainly regulated by blood flow. Apoptosis is implicated in the regression of large diameter blood vessels. In the small-diameter blood vessels that are remodeled by angiogenesis, pruning involves EC migration, self-fusion, and contraction before reabsorption into the remaining vasculature (Korn and Augustin, 2015; Betz et al., 2016). The model proposed by the authors of Chen et al. (2012) provided valuable insights into the role of hemodynamics during Zebrafish midbrain vascular pruning.

In conclusion, we developed a Boolean model of the network involved in EC behavior control during angiogenesis. The simulated dynamic behavior of our model corresponds with what has been observed experimentally and published about EC behavior and the effect of single gain- and loss-of-function mutations. The dynamical behavior of the model can qualitatively describe a wide variety of physiopathological states during angiogenesis. We believe that this characteristic makes the model a good platform to study the effect of altering the micro-environments and/or molecular backgrounds on endothelial cells.

## Author contributions

NW, LM, IG, and JK planned the research, wrote the article, analyzed, and discussed the results. NW reviewed the literature, composed the model, wrote the update rules, wrote the required scripts, and made the tables and figures. NW and LM carried out the simulations. IG and JK obtained funding for this project.

### Conflict of interest statement

The authors declare that the research was conducted in the absence of any commercial or financial relationships that could be construed as a potential conflict of interest.

## Abbreviations

AMP: Adenosine Monophosphate
AMPK: AMP-activated Protein Kinase
ANG: Angiopoietin
APLN: Apelin
ASF: Alternative Splicing Factor
ATP: Adenosine Triphosphate
BMP: Bone Morphogenetic Protein
BTrCP: Beta-Transducin Repeat Containing E3 Ubiquitin Protein Ligase
CXCR4: C-X-C motif chemokine Receptor 4
DAAM: Dishevelled Associated Activator Of Morphogenesis
DLL: Delta-Like canonical notch Ligand
DSH: Dishevelled
EC: Endothelial Cell
ECM: Extracellular Matrix
eNOS: Endothelial Nitric Oxide Synthase
EPC: Endothelial Progenitor Cells
EPH: Ephedrin
ERK: Extracellular signal-Regulated Kinase
FAK: Focal Adhesion Kinase
FOXO1: Forkhead Box O1
FGF: Fibroblast Growth Factor
FZD: Frizzled
GSK3β: Glycogen Synthase Kinase 3 Beta
HSC: Hematopoietic Stem Cell
HEY: Hes related family BHLH transcription factor with YRPW motif
HIF: Hypoxia-Inducible Factor
IA: Intussusceptive Angiogenesis
IGF: Insulin-like Growth Factor
JAG: Jagged
KLF: Kruppel Like Factor
LRP: LDL Receptor Related Protein
LEF: Lymphoid Enhancer binding Factor
MAPK: Mitogen-Activated Protein Kinase
MEK: Mitogen-Activated Protein Kinase Kinase (MAPKK)
MMP: Matrix Metalloproteinase
mTOR: mechanistic Target Of Rapamycin
NFAT: Nuclear Factor of Activated T-cells
NICD: NOTCH Intracellular Domain
NO: Nitric Oxide
Nox2: NADPH oxidase 2
NRARP: NOTCH Regulated Ankyrin Repeat Protein
NRP1: Neuropilin 1
PA: Plasminogen Activator
PDGF: Platelet-Derived Growth Factor
PECAM: Platelet and Endothelial Cell Adhesion Molecule
PI3K: Phosphatidylinositol-4,5-bisphosphate 3-Kinase
PIP3: Phosphatidylinositol (3,4,5)-trisphosphate
PKC: Protein Kinase C
PTEN: Phosphatase and Tensin homolog
RHO: Rhodopsin
SA: Splitting Angiogenesis
SC: Stalk Cell
SF2: pre-mRNA-Splicing Factor 2
SIRT: Sirtuin, S1P, sphingosine-1-Phosphate
S1PR: sphingosine-1-Phosphate Receptor
TC: Tip Cell
TGF: Transforming Growth Factor
TIE: Tyrosine kinase with domains similar to Immunoglobulin and Epidermal growth factor
TSC: Tuberous Sclerosis
uPAR: Urokinase Receptor
VEGF: Vascular Endothelial Growth Factor
VEGFR: Vascular Endothelial Growth Factor-Receptor.
