# Supplementary material for: A Network Model to Explore the Effect of the Micro-environment on Endothelial Cell Behavior during Angiogenesis
Source: Front Physiol. 2017 Nov 27;8:960. doi: 10.3389/fphys.2017.00960 (PMC5711888; doi:10.3389/fphys.2017.00960)
Supplement: Supplementary file 1 [file SupplementaryMaterial.PDF]

Supplementary Material: A network model to explore the effect of the micro-environment on endothelial cell behavior during angiogenesis

Nathan Weinstein [1], **Luis Mendoza,**[3] **Isidoro Gitler** [1] **and Jaime Klapp** [1,2,*]
*Correspondence:
Jaime Klapp
jaime.klapp@inin.gob.mx

Nathan Weinstein
nathan.weinstein4@gmail.com

1 MOLECULAR BASIS OF THE REGULATORY NETWORK

A molecular regulatory network is involved in the control of angiogenesis. This network is able to respond to shear stress, mechanical stretch, as well as the concentration of several molecular cues in the extracellular microenvironment of an endothelial cell (Otrock et al., 2007; Herbert and Stainier, 2011; Geudens and Gerhardt, 2011; Jin et al., 2014; Sewduth and Santoro, 2016; Sivaraj and Adams, 2016; Ferrara et al., 2003; Abhinand et al., 2016; Simons et al., 2016). The following subsections provide the evidence used to infer the regulatory network that controls angiogenesis (see Figure 2 and Supplementary section 2).

1.1 Induction of angiogenesis and the regulation of vascular endothelial growth factors

Hypoxia, the metabolic state of cells, shear stress and cytokines are the main triggers for angiogenesis. These factors act mainly by regulating the activity of the Vascular endothelial growth factor (VEGF) signaling pathway. VEGFs are structurally related dimeric proteins that include vertebrate VEGFA, VEGFB, VEGFC, and VEGFD, placenta growth factor (PlGF), parapoxvirus VEGFE, and snake venom VEGFF. The main agonist involved in vascular development is VEGFA. VEGFB is involved in endothelial cell fatty acid absorption, particularly in the heart. VEGFC and VEGFD are capable of inducing angiogenesis and lymphangiogenesis (Leppänen et al., 2010, 2011). Finally, inflammation-associated angiogenesis requires PlGF.

Oxygen availability is sensed by nitric oxide synthase (eNOS), NADPH oxidases, heme oxygenase and Hypoxia-inducible factors (HIFs). Under hypoxic conditions cells adjust the oxidative metabolism, causing the accumulation of certain metabolites such as lactate and citrate (Kumar et al., 2014). Hypoxia, Ca^{2+} and the metabolic state of cells regulate *VEGFA* expression. HIF1 is the main mediator of the cellular response to hypoxia, where O_2 availability is insufficient to meet tissue metabolic requirements. HIF1 consists of two subunits: HIF1α, which is targeted for proteasomal degradation when sufficient O_2 is present in the micro-environment, and constitutively expressed HIF1b. HIF2α is an HIF1α paralogue that is involved in vascular responses to ischaemia, and activates the transcription of VE-PTP (Eklund et al., 2017). Elevated levels of NAD dependent deacetylase (SIRT1) resulting from hypoxia activate HIF2α, FOXO1 and PGC1, resulting in the expression of VEGFA (Kumar et al., 2014). HIF1 directly activates the transcription of VEGFA, ANG2, and NRP1. Additionally, HIF1 is a direct or indirect activator of the expression of PGF

and PDGFB. Furthermore, HIF1 regulates the expression of cell surface receptors that allow endothelial cells to respond to hypoxia-induced angiogenic factors (Forsythe et al., 1996; Pugh and Ratcliffe, 2003; Rey and Semenza, 2010).

Lactate upregulates *VEGF2* transcription (Simons et al., 2016), and inactivates PHDs, thus allowing HIF1 to activate the expression of VEGFA. Also, hypoxia as well as an increase in the AMP/ATP ratio cause an increase in the activity of AMP Kinase (AMPK), which in turn promotes an increase in NAD+ concentration, leading to the activation of SIRT1. SIRT1 is involved in two positive feedback circuits. First, SIRT1 activates HIF1 and HIF2 by deacetylation, and they in turn positively regulate the transcription of SIRT1 (Chen et al., 2011; Laemmle et al., 2012). And second, SIRT1 activates FOXO1, which up–regulates the transcription of SIRT1 (Xiong et al., 2011). Then, during conditions of energy starvation AMPK is activated, acting through its downstream effector mTOR to upregulate VEGFA. This forms an autocrine feedback loop where VEGFA stimulates VEGFA production via VEGFR2-dependent activation of mTOR (Kumar et al., 2014). Finally, NFAT downregulates *VEGFA* expression (Chang et al., 2004), while STAT3 activates *VEGFA* gene transcription (Simons et al., 2016).

VEGFA mRNA splicing can generate pro-angiogenic proteins (VEGFA$_{xxx}$), or anti-angiogenic proteins (VEGFA$_{xxx}$b). The difference between the two protein families are the six amino acids closest to the C terminal, namely a change from CDKPRR to SLTRKD. Pro-angiogenic splicing is mediated by insulin-like growth factor (IGF). IGF activates protein kinase C (PKC), which causes the phosphorylation of SR protein kinases (SRPKs) that activate the ASF-SF2 splicing factor. These process may depend on the presence of HIF. TGFβ1 causes p38 MAPK activation, which activates the kinases CLK1 and CLK4. These kinases phosphorylate the splicing factor SRP55, which favors anti-angiogenic splicing (Harper and Bates, 2008).

One of the mot important post-translational modifications that reduce the angiogenic potential of VEGFA is poly-ADP ribosylation (PAR). NAD inhibits ART1-mediated PAR modification of VEGFA and the NAD/NADH ratio is negatively regulated by the availability of oxygen. Furthermore, NO activates cytosolic ADP ribosyl transferase (Kumar et al., 2014). Finally, certain proteases such as plasmin may convert ECM-bound VEGFA into a freely diffusible form (Ferrara, 2010).

1.2 Regulation of VEGFR activity

There exist three main receptor tyrosine kinases (RTKs) that bind with high affinity to VEGFs: VEGFR1, VEGFR2 and VEGFR3. During canonical VEGFR2 activation, a VEGF dimer binds to a VEGFR, causing VEGFR dimerization and activation by autophosphorylation on certain tyrosine residues. Noncanonical VEGFR activation involves VEGF-independent mechanisms such as mechanical forces that may also cause receptor dimerization and activation (Simons et al., 2016). The relative availability of different VEGF ligands influences the dimeryzation process. Specific ligands favor the formation of homodimers, while ligands that bind more than one receptor favor the formation of heterodimers (Mac Gabhann and Popel, 2007). There are also VEGF co-receptors, including neuropilin (NRP) family members NRP1 and NRP2, as well as heparan sulfate proteoglycans (HSPGs).

VEGFR2, also known as KDR (Kinase Insert Domain Receptor), is one of the main molecules involved in the control of vascular network formation and remodeling. The transcription and activity of VEGFR2 are tightly regulated. FGF induces *VEGFR2* gene expression by activating the adapter protein FRS2α that activates the MEK-ERK1/2 pathway. ERK1/2 then causes ETS transcription factor activation, and promotes ETS binding to the FOX-ETS motif in the first intron enhancer of *VEGFR2* (Murakami et al., 2011). VEGFA binding to a VEGFR2 homodimer induces the activation of the PLCγ-ERK1/2 pathway and activates ETS mediated *VEGFR2* expression forming a positive feedback circuit. However, VEGFR2

signaling also activates AKT, and AKT phosphorylates RAF, inhibiting ERK1/2 signaling and thus forming a negative feedback circuit (Hong et al., 2006). Finally, the bHLH transcription factor HEY1 is a direct Notch signaling target that inhibits VEGFR2 expression (Holderfield et al., 2006).

VEGFR2 homodimers must be located at the plasma membrane in order to be activated by VEGFA, processed VEGFC, or processed VEGFD dimers. VEGFR2 dimers are maintained dephosphorylated and inactive when forming a complex with VEcadherin, VEPTP, and DEP1, at endothelial cell junctions (Simons et al., 2016). Additionally, VEPTP is also involved in TIE2-mediated VEGFR2 inhibition (Hayashi et al., 2013). Once activated, VEGFR2 dimers may activate PLCγ or SFKs both at the plasma membrane and within endosomes. Nonetheless, endosomal VEGFR angiogenic signaling is stronger and continues until VEGFR2 is dephosphorylated by PTP1B.

Vascular endothelial growth factor receptor 1 (VEGFR1; Fms-like tyrosine kinase (FLT1)) is expressed in endothelial cells, monocytes, macrophages, vascular smooth muscle cells, and neurons, among others. VEGFR1 binds to VEGFA, VEGFB and placenta growth factor (PlGF). PlGF exhibits VEGFR1-specific binding affinity and promotes the formation of VEGFR1 homodimers (Mac Gabhann and Popel, 2007). Notch signaling (Funahashi et al., 2010), ETS1 (Valter et al., 1999) and HIF1 (Gerber et al., 1997) induce *Vegfr1* expression, while NFAT inhibits its expression (Jinnin et al., 2008). VEGFR1 exhibits a high binding affinity (10 pM) to VEGFA. However, this binding induces VEGFR1 phosphorylation very weakly, and also promotes the expression of a splice variant encoding a soluble VEGFR1(sVEGFR1) that binds VEGFA. As a result, VEGFR1 acts as a decoy that reduces the amount of VEGFA available to bind VEGFR2. Then, the activation of the VEGF1-VEGF2 heterodimer stimulates cell migration and NO production, failing to induce endothelial cell proliferation. Additionally, VEGF1-VEGF2 heterodimers inhibit VEGFA-mediated ERK1/2 activation and Ca^2+ uptake, suggesting that VEGF1-VEGF2 heterodimers may negatively regulate VEGFR2 homodimer activation (Simons et al., 2016).

Similarly to sVEGFR1, connective tissue growth factor (CTGF), platelet factor 4 (PF4), and heparin affin regulatory peptide (HARP) bind VEGF, with the result of reducing its availability to activate VEGF-driven angiogenic behavior. However, human MMPs degrade VEGF inhibitors. In particular, MMP7 degrades soluble VEGFR1, thus increasing the sensibility of endothelial cells to VEGF (Ito et al., 2009).

VEGFR3 expression is directly upregulated by Notch signaling (Shawber et al., 2007), and can be found in macrophages, osteoblasts, and neuronal progenitors, as well as lymphatic, venous, arterial and capillary endothelial cells during early development. Thereafter, VEGFR3 expression is recovered in endothelial cells during certain types of angiogenesis. Unprocessed VEGFC and VEGFD only bind VEGFR3 and promote the formation of VEGFR3 active homodimers that induce the activation of ERK1/2 (Simons et al., 2016). Proteolytically processed VEGFC and VEGFD bind to both VEGFR2 and VEGFR3 with high affinity, promoting VEGFR2-VEGFR3 heterodimer formation. VEGFA189 can also induce VEGFR2-VEGFR3 heterodimers. Embryonic and adult lymphangiogenesis requires the formation of VEGFR2-VEGFR3-NRP1 complexes that activate AKT signaling. Finally, vascular endothelial PTP (VEPTP) acts as a VEGFR3 tyrosine phosphatase in lymphatic endothelium and modulates ERK1/2 and AKT activation (Simons et al., 2016).

1.3 VEGF signaling activates the signaling pathways ERK1/2, PI3K-AKT, SRC and p38 MAPK, and also phosphorylates STATs

In endothelial cells, VEGF signaling mediates the phosphorylation of STATs, which constitute a family of SH2 domain-containing proteins, promoting their nuclear translocation. VEGF also signals through the PLCγ-ERK1/2 pathway, regulating endothelial cell homeostasis, migration, proliferation,

and differentiation into arterial endothelium. When VEGFR2 is phosphorylated on tyrosine 1175, it binds to and activates PLCγ, which then induces the generation of inositol 1,4,5-trisphosphate (IP_3) and diacylglycerol (DAG). IP_3 causes the release of Ca^2+ from the endoplasmic reticulum, enabling DAG to induce the activation of Ca^{2+}-dependent protein kinase Cβ2 (PKCβ2). PKCβ2 then activates the RAF1, MEK and ERK1/2 signaling cascade leading to the activation of ETS transcription factors. In particular, ETS1 activates the transcription of NRP1, integrin β3, VE-cadherin, and the metaloproteases MMP1, MMP3, and MMP9 (Teruyama et al., 2001). Additionally, Ca^{2+} signaling is necessary for the activation of the nuclear factor of activated T cell (NFAT) family of transcription factors. The Ca^{2+} sensor Calmodulin (CaM) activates the Ca^{2+}-dependent serine/threonine phosphatase Calcineurin to regulate the transcriptional activation and the nuclear translocation of NFAT proteins (Simons et al., 2016). Furthermore, Ca^{2+} activates the protein tyrosine kinase 2β (PTK2β; also known as RAFTK or PYK2). Finally, SRC and PTK2β activate p38 MAPK.

The AKT serine/threonine kinases regulate endothelial cell survival, proliferation and apoptosis. Phosphorylation of tyrosine 951 in VEGFR2 causes TSAd-mediated activation of Src family kinases (SFKs), leading to the activation of VE-cadherin and AXL. VE-cadherin and AXL then activate PI3K (Ruan and Kazlauskas, 2012; Simons et al., 2016). PI3K catalyzes PIP2 phosphorylation, generating phosphatidylinositol-3,4,5-trisphosphate (PIP3), while the phosphatase and tensin homolog (PTEN) catalyzes the dephosphorylation of PIP3. PIP3 activates AKT by binding to its plextrin homology (PH) domain (Karar and Maity, 2011). AKT ativation induces FOXO1 and eNOS-mediated NO generation, and leads to the phosphorylation and activation of mammalian target of rapamycin (mTOR). Then, activated eNOS forms a complex with CaM that upregulates NO production. Guanylate cyclase (sGC) binds NO to produce sGC-NO, which increases the concentration of cGMP. This molecule inhibits TRPV4 (White et al., 2016), reducing the concentration of Ca^{2+} and inhibiting CaM, thus forming a negative feedback circuit (Sriram et al., 2016). Tuberous sclerosis complex 1 (TSC1) binds TSC2 and inhibits the activity of the small GTPase RHEB. AKT directly phosphorylates TSC2 allowing RHEB-mediated mTOR activation. Additionally, AKT inhibits AMPK activity and TSC2 is directly phosphorylated and activated by AMPK (Hay, 2005). Finally, mTOR activation likely upregulates HIF1 translation (Karar and Maity, 2011).

SRC signaling regulates vascular permeability and cell adhesion. SRC, YES and FYN are members of the SRC family of endothelial cytoplasmic tyrosine kinases. When VEGFR2 is phosphorylated at tyrosin 951, it binds to the SH2 domain of T cell- specific adapter (TSAd). TSAd then binds to the SH3 domain of SRC. Focal adhesions contain focal adhesion kinase (FAK), its substrate paxillinan and other signal transducers. FAK and SRC may activate each other, forming a positive feedback circuit. VEGF or mechanical stimuli regulate cell shape, cell adhesion and vascular leakage by causing SRC-mediated phosphorylation of FAK. One of the mechanisms that allow SRC to regulate cell adhesion and vascular permeability involves the disruption of adherens junctions by phosphorylating and causing the endocytosis of VE-cadherin (Simons et al., 2016).

1.4 Shear stress regulates VEGF signaling

Shear stress and circumferential stress regulate VEGF, NO and Ca^{2+} signaling (Kutys and Chen, 2016). One of the molecular mechanisms that allows mechanical forces to activate VEGFR2 signaling involves the formation of a mechanosensory complex located at adherens junctions (AJs) at the apical or lateral EC membrane. The complex includes vascular endothelial cadherin (VEcadherin), platelet endothelial cell adhesion molecule 1 (PECAM1, also known as CD31), and VEGFR2 or VEGFR3. Flow causes shear stress, which deforms actin and the intermediate fiber networks in the cytoskeleton. The change in filament

tension reaches the AJs where it causes an increase in tension across PECAM1 and a decrease in tension across VE-cadherin (Coon et al., 2015; Kutys and Chen, 2016).

The cytoplasmic tail of PECAM1 contains two immunoreceptor tyrosine-based inhibitory motifs (ITIMs) that are phosphorylated when shear stress originates conformational changes. The changes allow a Src family kinase (SFK) —probably Fyn— to phosphorylate one of the ITIMs on tyrosine 713 and recruit tyrosine protein phosphatase SHP2 to stimulate the ERK1/2 signaling. Mechanical activation of PECAM1 also causes ligand-independent VEGFR2-3 phosphorylation, leading to the activation of the MAPK, PI3K, and AKT signaling pathways (Jin et al., 2003; Chistiakov et al., 2016; Kutys and Chen, 2016).

Blood flow activates the mechanosensitive transcription factor Krüppel-like factor 2 (*KLF2*), which promotes *miR-126* expression. *miR-126* positively regulates VEGF signaling by post-transcriptionally repressing the inhibitor of Ras SPRED1 (Sprouty- related EVH1 domain-containing 1) (Pasmant et al., 2015), and also repressing the inhibitor of PI3K signaling PI3K regulatory subunit 2 (PIK3R2; also known as p85). Also, KLF2 is able to increase VEGFA expression.

Shear stress may induce matrix metalloproteinase-mediated release of VEGFA from the ECM (Herbert and Stainier, 2011; Chistiakov et al., 2016), and also activates G-protein-coupled receptors (GPCRs) leading to G protein activation (Sriram et al., 2016). G proteins Gq and G11 activate SRC and AKT signaling leading to eNOS, PECAM1, and VEGFR2 activation (Chistiakov et al., 2016). In addition, shear stress leads to the phosphorylation at tyrosine Y110 of TRPV4, which then accumulates in the plasma membrane. Furthermore, shear stress activates PIEZO1; both PIEZO1 and TRPV4 augment the intracellular concentration of Ca^{2+} in ECs (Chistiakov et al., 2016). Integrins anchor ECs to the extracellular matrix, and are connected to focal adhesion sites. These integrins transduce mechanical forces by phosphorylating FAKs, thus triggering the activation of PI3K (Sriram et al., 2016).

1.5 The Notch signaling pathway

The Notch signaling pathway is involved in the regulation of cell differentiation, proliferation and survival during development and aging. Notch signaling is initiated when a ligand from the Delta-like 1 (DLL1, DLL3, DLL4) or Jagged1 (JAG1, JAG2) subfamily binds to a Notch receptor. After ligand binding, the Notch receptor (NOTCH1-4) is proteolytically cleaved at extracellular site S2 by a disintegrin and metalloproteinase (ADAM17 or ADAM10). Later, the Notch receptor is proteolytically cleaved at the transmembranal site S3 by a γ-secretase complex composed of presenlin (PSEN1, PSEN2), presenilin enhancer 2 (PEN2), anterior pharynx defective 1 (APH1) and nicastrin (NCSTN). After cleavage, the remaining Notch intracellular domain (NICD) is transported to the nucleus, where it binds the ubiquitous transcription factor CSL, and recruits co-activator mastermind-like (MAML) proteins as well as the histone acetylase CBP/p300, forming a complex that activates the transcription of several target genes.

The expression of Hairy/enhancer of split (HES) and HES-related proteins (HEY/HRT/HERP), PTEN (Serra et al., 2015), DLL4, and NRP1 is directly regulated by Notch signaling (Blanco and Gerhardt, 2013). Also, Notch signaling prevents SIRT1 and LSD1 from inhibiting the transcription of several Notch signaling targets (Morris, 2013). Importantly, Notch signaling is involved in two endothelial cell differentiation processes during vascular development; namely, artery-vein fate specification during vasculogenesis and tip-stalk cell differentiation during angiogenesis (Blanco and Gerhardt, 2013; Andersson and Lendahl, 2014; Cai et al., 2016).

1.6 The Wnt signaling pathway

The Wnt/β-catenin or canonical Wnt signaling pathway and two non-canonical Wnt pathways are involved in vascular remodeling control; namely, the Wnt-Ca^{2+} signaling pathway and the polarity/planar cell polarity PCP pathway. The particular pathway that is activated depends on the composition of the ligand/receptor/co-receptor complex (Reis and Liebner, 2013; Wang et al., 2016b; Peghaire et al., 2016). ECs can express the Wnt ligands *WNT2, WNT2b, WNT3, WNT4, WNT5a, WNT5b, WNT7a, WNT8a, WNT9a, WNT9b*, and *WNT11* (Franco et al., 2009). Additionally, ECs express Wnt Frizzled receptors *FZD1, FZD2, FZD4, FZD5, FZD6, FZD7, FZD9, FZD10*, low density lipoprotein receptor-related protein co-receptors *LRP5* and *LRP6*. The Wnt signaling inhibitors RYK, sFRP1, and sFRP3 negatively regulate Wnt ligand availability, while DKK1 and DKK3 inhibit LRP5/6 function (Franco et al., 2009; Newman and Hughes, 2012).

Wnt/β-catenin signaling is activated when certain Wnt ligands (WNT-1, -3, -3a, -7a and -7b) simultaneously bind a receptor (FZD4,7) and a co-receptor (LRP5,6). Wnt binding leads to LRP phosphorylation, and recruitment of dishevelled (DSH) at the plasma membrane. Then LRP and DSH bind AXIN, inactivating a complex composed of AXIN, Adenomatous polyposis coli (APC), Casein kinase 1α (CK1α), and Glycogen synthase kinase-3β (GSK3β). CK1α and GSK3β then phosphorylate β-catenin allowing the E3 ubiquitin ligase β-TrCP to bind and mark the protein for proteasomal degradation. As the function of the complex is inhibited, the concentration of β-catenin (CTNNB1) in the cytoplasm rises. CTNNB1 then translocates to the nucleus where it replaces the transcription inhibitor Groucho by binding a DNA-bound T cell factor/lymphoid enhancer-binding factor (TCF/LEF) transcription factor, and activating the transcription of several target genes. Additionally, CTNNB1 is a scaffold protein that links the cytoplasmic tail of VE-cadherin and N-cadherin to α-catenin and the actin network (Reis and Liebner, 2013). Finally, the expression of the genes *AXIN2* and *LEF1* is positively regulated by CTNNB1/TCF.

The Wnt-Ca^{2+} signaling pathway is activated when a Wnt ligand (WNT5a) binds to a Fzd receptor (FZD4,5), and ROR1/2 co-receptors induce DSH, leading to the activation of the protein phospholipase C (PLC) by DSH, causing the intracellular Ca^{2+} concentration to increase (Franco et al., 2009). By contrast, when a Wnt ligand (WNT5a) binds to a Fzd receptor (FZD4,5), and associate to the co-receptors KNY, ROR2 and RYK, or RAC1 from the PCP pathway, it may activate c-Jun N-terminal kinase (JNK), leading to activator protein 1 (AP1)-mediated transcription of specific target genes (Reis and Liebner, 2013).

The Wnt-PCP pathway is activated when certain Wnt ligands (WNT5a,11) bid to specific receptors (FZD6) and ROR1/2 coreceptors, causing DSH-mediated activation of the small GTPases CDC42, RHOA/B, and RAC1. This leads to cell polarization and cytoskeletal rearrangements. RAC1 is directly activated by DSH. In contrast, the activation of CDC42 and RHOA/B involves dishevelled-associated activator of morphogenesis 1 (DAAM1) function downstream of DSH. In addition, DAAM1 inhibits EC migration and proliferation. Furthermore, RHOA phosphorylates and activates PTEN (Meili et al., 2005) leading to the inhibition of the AKT signaling pathway.

1.7 Notch and Wnt regulate EC proliferation

Notch signaling activates the expression of the Notch-regulated ankyrin repeat protein (*NRARP*) gene in stalk cells. NRARP negatively regulates Notch signaling by promoting NICD degradation. NRARP is able to induce cell cycle progression by binding to and stabilizing lymphoid enhancer factor 1 (LEF1). In turn, LEF1 forms a complex with CTNNB1, which activates the transcription of Cyclin D1 (*CCND1*). Finally,

NRARP interferes with p21/Rb-dependent cell-cycle arrest by limiting Notch signaling (Phng et al., 2009; Korn and Augustin, 2015).

1.8 Blood vessel stability

The ANG/TIE signaling pathway acts as gatekeeper of EC quiescence. When a blood vessel is exposed to shear stress and requires stabilization, the cell secretes PDGFB to attract mural cells. Once mural cells attach to the ECs, they secrete ANG1 and TGFβ1, and thus inhibit the secretion of PDGFB by EC (Nishishita and Lin, 2004). The ANG1/TIE2 signaling induces EC quiescence and PI3K/AKT-mediated EC survival (Eklund et al., 2017).

ANG2 is an ANG1/TIE2 signaling antagonist that destabilizes blood vessels, causes mural cell migration, and allows the remodeling of the blood vessel network in part by directly activating β_1 integrin. If VEGF signaling is inactive, ANG2 signaling induces capillary regression and EC death. By contrast, if VEGF signaling is active, then ANG2 promotes sprout formation, and EC proliferation and migration.

TIE1 is recruited to the TIE2 signaling complex at EC junctions after the binding of TIE2 by ANG1 or ANG2, a process mediated by β_1 integrin (Korn and Augustin, 2015). In stable blood vessels, ANG1 activates the Tie2/Akt pathway, leading to AKT-mediated FOXO1 phosphorylation and nuclear exclusion. During vascular remodeling, FOXO1 activates the transcription of *ANG2* and *VEGFA*, thus forming a positive feedback mechanism that promotes blood vessel destabilization. Furthermore, FOXO1 (Uebelhoer et al., 2013) SMAD2, and SMAD3 (Taylor and Khachigian, 2000) activate the expression of PDGFB and mural cell recruitment. Additionally, TIE1 negatively regulates ANG-induced TIE2 internalization. During acute inflammation, TIE1 cleavage causes the nuclear translocation of FOXO1, *ANG2* transcription, and blood vessel destabilization (Korhonen et al., 2016).

Shear stress directly regulates ANG/TIE signaling. Specifically, the transcription factor KLF2 downregulates *ANG2* and upregulates *TIE2* expression. Non canonical Wnt siganling via AP1 and ETS1 upregulates *ANG2* and *TIE2* transcription (Ye et al., 2007; Newman and Hughes, 2012). Finally, VEPTP regulates blood vessel stability through the dephosphorylation of TIE2 and VE-cadherin (Frye et al., 2015).

1.9 The transforming growth factor (TGF) signaling pathway

Upon the binding of a dimeric TGFβ ligand to an heteromeric complex composed of one transmembrane type I dimer and one type II serine/threonine kinase receptor dimer, the type II receptors phosphorylate type I receptors. This causes the phosphorylation of receptor-regulated SMAD (R-SMAD) proteins, which consequently form a complex with SMAD4 (Co-Smad) and enter the nucleus to alter gene transcription. Additionally, type III receptors function as co-receptors (Weiss and Attisano, 2013). In this process, the ligand determines the specific combination of type I and type II receptors. In turn, the composition of the ligand-receptor complex determines which SMADs will be phosphorylated, and therefore, which genes will be regulated.

The components of the human TGFβ signaling pathway include at least 30 ligands: 3 TGFβs, 4 ACTIVINs, 10 bone morphogenetic proteins (BMPs), 11 growth and differentiation factors (GDFs), anti-Müllerian hormone (AMH; Müllerian inhibitory substance) and NODAL. In turn, the ligands can be subdivided into two functional groups: the TGFβ-like group that includes TGFβs, Activins, Nodals and some GDFs; and the BMP-like group comprised of BMPs, most GDFs, and AMH.

There are seven activin receptor-like kinase type I receptors (ALK1-7), and five type II receptors: Müllerian inhibiting substance receptor II (MISRII), activin receptor type-2A and -2B (ACVR2A and

ACVR2B), bone morphogenic protein receptor II (BMPRII), and transforming growth factor β receptor II (TGFβRII). Also, the type III receptors —endoglin (ENG) and betaglycan (BG)—, function as TGFβ co-receptors.

Regarding SMADs, there exist two groups that activate gene transcription, conformed by SMAD2,3 and SMAD1,5,9. Conversely, SMAD6 and SMAD7 inhibit the transcription of certain genes. Lastly, SMAD4 functions as a scaffolding co-factor (Weiss and Attisano, 2013).

ALK1, TGFβRII, BMPRII, ACVR2A and the co-receptors ENG and BG are expressed within ECs in vivo. ALK1 and ENG in particular are highly enriched in ECs. In ECs TGFβ1, BMP9 and BMP10 bind to ALK1 and ENG to induce pSMAD1/5/8 signaling (Jin et al., 2014; Maring et al., 2016). Recent studies suggest that BMP9 binds to ALK1 to induce pSMAD2/3 signaling (Aspalter et al., 2015). Additionally, after TGFβ ligand binding, type I or type II receptors may activate Ras/MAPK signaling (Weiss and Attisano, 2013). Furthermore, TGFβ receptors cause Smad-independent p38 MAPK activation via TRAF6 and TAK1 (Yamashita et al., 2008).

1.10 Differentiation of tip and stalk cells

The molecular mechanism involved in the regulation of EC differentiation into tip and stalk cells is an excellent example of Notch-mediated lateral inhibition (Glass et al., 2016). VEGF signaling induces tip cell fate specification, and then tip cells induce neighboring cells to differentiate into stalk cells, preventing them from becoming tip cells, mediated by DLL4/Notch signaling, which reduces the VEGF sensitivity of stalk cells. Lateral inhibition amplifies any initial differences in VEGF concentration and timing. Nonetheless, Tip/Stalk cell differentiation is a reversible process, and the EC compete for the tip cell position during sprouting angiogenesis.

The VEGF signaling pathway activates the transcription of Notch ligands and receptors. VEGF signaling, via PI3K, activates the Forkhead family transcriptions factors FOXC1 and FOXC2 that directly upregulate *DLL4* expression (Hayashi and Kume, 2008). Additionally, TEL and CtBP, which normally are occupying the *DLL4* promoter, transiently disassemble upon VEGFA stimulation, thus allowing for a transient transcription of *DLL4* (Roukens et al., 2010). Furthermore, the Vegf/MAPK pathway activates ETS transcription factors such as ERG and ETS1, which activate the transcription of *DLL4* and *NOTCH4* (Wythe et al., 2013). VEGF, via the phosphatidylinositol 3-kinase/Akt pathway, is also able to activate the transcription of *NOTCH1* (Liu et al., 2003). Stalk cells inhibit Notch signaling in tip cells by Wnt-mediated expression of the *JAG1* ligand. JAG1 antagonizes DLL4Notch signaling in the tip cells when the Notch receptors in tip cells are modified by the glycosyltranferase Fringe (Blanco and Gerhardt, 2013).

Notch, Wnt, and TGFβ signaling pathways interact to repress tip cell specification, favor stalk cell fate determination, and regulate vascular sprouting, although the process is not fully understood. In stalk cells the pSMAD1/5 signaling represses the expression of the tip-cell associated genes *CXCR4, DLL4*, and Apelin *APLN*. Both CTNNB1 and pSMAD1/5 directly activate the expression of JAG1, as well as the expression of inhibitor of DNA-binding (ID) proteins, which interact with the Notch signaling by forming complexes with HES1 and HEY1. pSMAD1/5 and pSMAD2/3 signaling also activate the expression of several Notch target genes including *HEY1, HEY2* and *HES1*, thus decreasing the cell responsiveness to the VEGF signaling (Jin et al., 2014; Zavadil et al., 2004). In tip cells, NRP1 inhibits ALK1/BMP9-mediated SMAD2/3 phosphorylation. Notch activation in stalk cells negatively regulates NRP1 levels leading to higher pSMAD2/3 activity and stalk cell behavior(Aspalter et al., 2015). However, NOTCH signaling also inhibits TGF/BMP signaling by activating the expression of SMAD6 (Mouillesseaux et al., 2016).

2 THE UPDATE RULES OF OUR EXTENDED MODEL

$$ANG1(t+1) = ANG1(t) \tag{1}$$

$$ANG2(t+1) = (\neg KLF2(t)) \land (HIF1(t) \lor AP1(t) \lor ETS(t) \lor FOXO1(t)) \tag{2}$$

$$TIE1(t+1) = (Integrin(t) \lor ANG1(t) \lor ANG2(t)) \tag{3}$$

$$\begin{aligned} TIE2(t+1) = & TIE1(t) \land ANG1(t) \land (\neg ANG2(t)) \land \\ & ((\neg VEPTP(t)) \lor KLF2(t) \lor ETS(t)) \end{aligned} \tag{4}$$

$$VEPTP(t+1) = HIF2a(t) \tag{5}$$
$$ShearStress(t+1) = ShearStress(t) \tag{6}$$
$$mir126(t+1) = KLF2(t) \tag{7}$$
$$SPRED1(t+1) = \neg mir126(t) \tag{8}$$
$$PI3KR2(t+1) = \neg mir126(t) \tag{9}$$
$$NF1(t+1) = SPRED1(t) \tag{10}$$
$$KLF2(t+1) = ShearStress(t) \tag{11}$$
$$PIEZO1(t+1) = ShearStress(t) \tag{12}$$
$$TRPV4(t+1) = ShearStress(t) \wedge \neg cGMP(t) \tag{13}$$
$$Integrin(t+1) = (ShearStress(t) \vee TIE2(t)) \wedge ETS(t) \tag{14}$$
$$GqG11(t+1) = ShearStress(t) \tag{15}$$
$$Actin(t+1) = ShearStress(t) \tag{16}$$
$$VEcadherin(t+1) = ETS(t) \wedge (SRC(t) \vee FAK(t) \vee Actin(t) \vee VEPTP(t)) \tag{17}$$
$$PECAM1(t+1) = FYN(t) \vee Actin(t) \vee VEcadherin(t) \tag{18}$$
$$SHP2(t+1) = PECAM1(t) \tag{19}$$
$$FAK(t+1) = SRC(t) \vee Integrin(t) \tag{20}$$
$$SRC(t+1) = FAK(t) \vee TSAd(t) \vee GqG11(t) \tag{21}$$
$$PI3K(t+1) = AXL(t) \vee (\neg PI3KR2(t)) \vee VEcadherin(t) \vee TIE2(t) \tag{22}$$
$$AKT(t+1) = PIP3(t) \tag{23}$$
$$TSC(t+1) = AMPK(t) \wedge (\neg AKT(t)) \tag{24}$$
$$AXL(t+1) = SRC(t) \tag{25}$$
$$PIP3(t+1) = PI3K(t) \wedge (\neg PTEN(t)) \tag{26}$$
$$RHEB2(t+1) = \neg TSC(t) \tag{27}$$
$$FYN(t+1) = TSAd(t) \tag{28}$$
$$TSAd(t+1) = VEGFR22(t) \vee VEGFR23(t) \tag{29}$$
$$PTEN(t+1) = CSL(t) \vee RHO(t) \tag{30}$$
$$BMP9(t+1) = BMP9(t) \tag{31}$$
$$BMP10(t+1) = BMP10(t) \tag{32}$$
$$TGFB1(t+1) = TGFB1(t) \tag{33}$$
$$ACVR2A(t+1) = 1 \tag{34}$$
$$BMPRII(t+1) = 1 \tag{35}$$
$$TGFBRII(t+1) = 1 \tag{36}$$
$$\begin{aligned} ALK1(t+1) = &(TGFB1(t) \vee BMP9(t) \vee BMP10(t)) \wedge \\ &(ACVR2A(t) \vee BMPRII(t) \vee TGFBRII(t)) \end{aligned} \tag{37}$$

$$ENG(t+1) = TGFB1(t) \vee BMP9(t) \vee BMP10(t) \tag{38}$$
$$ALK5(t+1) = BMP9(t) \wedge TGFBRII(t) \tag{39}$$
$$BG(t+1) = BMP9(t) \tag{40}$$
$$SMAD4(t+1) = 1 \tag{41}$$
$$SMAD6(t+1) = CSL(t) \tag{42}$$
$$SMAD1(t+1) = (\neg SMAD6(t)) \wedge (\neg NRP1(t)) \wedge ALK1(t) \wedge ENG(t) \wedge SMAD4(t) \tag{43}$$
$$SMAD2(t+1) = (\neg SMAD6(t)) \wedge (\neg NRP1(t)) \wedge ALK5(t) \wedge BG(t) \wedge SMAD4(t) \tag{44}$$
$$PDGFB(t+1) = FOXO1(t) \vee SMAD2(t) \tag{45}$$
$$TRAF6(t+1) = ALK1(t) \tag{46}$$
$$TAK1(t+1) = TRAF6(t) \tag{47}$$
$$sGC(t+1) = 1 \tag{48}$$
$$cGMP(t+1) = NO(t) \wedge sGC(t) \tag{49}$$
$$eNOS(t+1) = AKT(t) \vee SIRT1(t) \tag{50}$$
$$NO(t+1) = eNOS(t) \vee Calmodulin(t) \tag{51}$$
$$Calcium(t+1) = IP3(t) \vee TRPV4(t) \vee PIEZO1(t) \tag{52}$$
$$Calmodulin(t+1) = Calcium(t) \tag{53}$$
$$Calcineurin(t+1) = Calmodulin(t) \tag{54}$$
$$NFAT(t+1) = Calcineurin(t) \tag{55}$$
$$ETS(t+1) = ERK(t) \tag{56}$$
$$RAS(t+1) = SHP2(t) \vee ALK1(t) \vee (\neg NF1(t)) \tag{57}$$
$$RAF1(t+1) = (PKCB2(t) \vee RAS(t)) \wedge (\neg AKT(t)) \tag{58}$$
$$MEK(t+1) = RAF1(t) \vee FRS2a(t) \tag{59}$$
$$ERK(t+1) = MEK(t) \vee VEGFR33(t) \tag{60}$$
$$PLCg(t+1) = VEGFR22(t) \vee VEGFR33(t) \vee DSH(t) \vee FZD4\_5(t) \tag{61}$$
$$IP3(t+1) = PLCg(t) \tag{62}$$
$$DAG(t+1) = PLCg(t) \tag{63}$$
$$PKCB2(t+1) = DAG(t) \wedge Calcium(t) \tag{64}$$
$$AMPATP(t+1) = AMPATP(t) \tag{65}$$
$$AMPK(t+1) = (AMPATP(t) \vee (\neg Oxygen(t))) \wedge (\neg AKT(t)) \tag{66}$$
$$mTOR(t+1) = AMPK(t) \vee RHEB2(t) \tag{67}$$
$$HIF1(t+1) = mTOR(t) \wedge \neg PHDs(t) \wedge SIRT1(t) \tag{68}$$
$$FOXO1(t+1) = SIRT1(t) \wedge (\neg AKT(t)) \tag{69}$$
$$ART1(t+1) = (\neg NAD(t)) \tag{70}$$
$$NAD(t+1) = AMPK(t) \tag{71}$$
$$Oxygen(t+1) = Oxygen(t) \tag{72}$$

$$Lactate(t+1) = \neg Oxygen(t) \tag{73}$$
$$PHDs(t+1) = Oxygen(t) \wedge \neg Lactate(t) \tag{74}$$
$$HIF2a(t+1) = SIRT1(t) \vee (\neg Oxygen(t)) \tag{75}$$
$$LSD1(t+1) = SIRT1(t) \wedge (\neg NICD(t)) \tag{76}$$
$$SIRT1(t+1) = (HIF2a(t) \vee HIF1(t) \vee FOXO1(t)) \wedge NAD(t) \tag{77}$$
$$WNT5a(t+1) = WNT5a(t) \tag{78}$$
$$ROR(t+1) = WNT5a(t) \tag{79}$$
$$JNK(t+1) = (ROR(t) \vee RAC1(t)) \wedge DSH(t) \tag{80}$$
$$AP1(t+1) = JNK(t) \tag{81}$$
$$FZD4\_5(t+1) = WNT5a(t) \tag{82}$$
$$WNT11(t+1) = WNT11(t) \tag{83}$$
$$FZD6(t+1) = WNT11(t) \vee WNT5a(t) \tag{84}$$
$$RAC1(t+1) = FZD6(t) \wedge DSH(t) \tag{85}$$
$$DAAM1(t+1) = FZD6(t) \vee DSH(t) \tag{86}$$
$$CDC42(t+1) = DAAM1(t) \tag{87}$$
$$RHO(t+1) = DAAM1(t) \vee NRP1(t) \tag{88}$$
$$WNT7a(t+1) = WNT7a(t) \wedge (\neg sFRP1(t)) \tag{89}$$
$$sFRP1(t+1) = 0 \tag{90}$$
$$FZD4\_7(t+1) = WNT7a(t) \tag{91}$$
$$LRP5\_6(t+1) = WNT7a(t) \wedge (\neg DKK1(t)) \tag{92}$$
$$DKK1(t+1) = 0 \tag{93}$$
$$DSH(t+1) = (FZD4\_5(t) \wedge ROR(t)) \vee FZD6(t) \vee (FZD4\_7(t) \wedge LRP5\_6(t)) \tag{94}$$
$$GSK3B(t+1) = \neg DSH(t) \wedge \neg LRP5\_6(t) \tag{95}$$
$$Axin2(t+1) = LEF1(t) \tag{96}$$
$$BTrCP(t+1) = 0 \tag{97}$$
$$Bcatenin(t+1) = \neg GSK3B(t) \wedge \neg BTrCP(t) \tag{98}$$
$$GROUCHO(t+1) = \neg Bcatenin(t) \tag{99}$$
$$LEF1(t+1) = (Bcatenin(t) \wedge \neg GROUCHO(t)) \wedge (NRARP(t) \vee LEF1(t)) \tag{100}$$
$$CyclinD1(t+1) = Bcatenin(t) \wedge LEF1(t) \tag{101}$$
$$FGF(t+1) = FGF(t) \tag{102}$$
$$FGFR2(t+1) = FGF(t) \tag{103}$$
$$FRS2a(t+1) = FGFR2(t) \tag{104}$$
$$JAGa(t+1) = SMAD1(t) \vee Bcatenin(t) \tag{105}$$
$$JAGp(t+1) = JAGp(t) \tag{106}$$
$$DLL4a(t+1) = ETS(t) \vee CSL(t) \tag{107}$$

$$DLL4p(t+1) = DLL4p(t) \tag{108}$$

$$ADAM10(t+1) = 1 \tag{109}$$

$$gSecretase(t+1) = 1 \tag{110}$$

$$Notch4(t+1) = ETS(t) \tag{111}$$

$$\begin{aligned} NOTCH(t+1) =& Notch4(t) \wedge gSecretase(t) \wedge ADAM10(t) \wedge \\ & DLL4p(t) \wedge (\neg JAGp(t)) \end{aligned} \tag{112}$$

$$NICD(t+1) = NOTCH(t) \wedge (\neg NRARP(t)) \tag{113}$$

$$CSL(t+1) = NICD(t) \tag{114}$$

$$NRARP(t+1) = CSL(t) \tag{115}$$

$$\begin{aligned} HEY1(t+1) =& CSL(t) \vee ((SMAD1(t) \vee SMAD2(t)) \wedge \\ & (\neg SIRT1(t) \wedge \neg LSD1(t))) \end{aligned} \tag{116}$$

$$Vegfr2(t+1) = (ETS(t) \wedge \neg HEY1(t)) \vee Lactate(t) \tag{117}$$

$$Vegfr1(t+1) = CSL(t) \vee ((HIF1(t) \vee ETS(t)) \wedge (\neg NFAT(t))) \tag{118}$$

$$PlGF(t+1) = PlGF(t) \tag{119}$$

$$VEGFR1s(t+1) = PlGF(t) \wedge Vegfr1(t) \tag{120}$$

$$VEGFB(t+1) = VEGFB(t) \tag{121}$$

$$VEGFR11(t+1) = Vegfr1(t) \tag{122}$$

$$VEGFR12(t+1) = Vegfr1(t) \wedge Vegfr2(t) \tag{123}$$

$$IGF(t+1) = IGF(t) \tag{124}$$

$$PKC(t+1) = IGF(t) \tag{125}$$

$$ASF\_SF2(t+1) = PKC(t) \tag{126}$$

$$VEGFAxxxP(t+1) = VEGFAxxxP(t) \tag{127}$$

$$\begin{aligned} VEGFAxxx(t+1) =& ((\neg(VEGFR1s(t) \vee VEGFR12(t) \vee VEGFR11(t)) \wedge \\ & VegfA(t) \wedge ART1(t) \wedge ASF\_SF2(t)) \vee VEGFAxxxP(t) \end{aligned} \tag{128}$$

$$p38MAPK(t+1) = TAK1(t) \vee SRC(t) \vee DAG(t) \quad (129)$$

$$CLK1(t+1) = p38MAPK(t) \quad (130)$$

$$CLK4(t+1) = p38MAPK(t) \quad (131)$$

$$SRP55(t+1) = CLK4(t) \vee CLK1(t) \quad (132)$$

$$VEGFAxxxd(t+1) = VegfA(t) \wedge SRP55(t) \quad (133)$$

$$VEGFC\_Dp(t+1) = VEGFC\_Dp(t) \quad (134)$$

$$VEGFC\_D(t+1) = VEGFC\_D(t) \quad (135)$$

$$VEGFR22(t+1) = Vegfr2(t) \wedge (PECAM1(t) \vee ((VEGFC\_Dp(t) \vee VEGFAxxx(t)) \wedge \neg(VEGFAxxxd(t) \vee VEPTP(t)))) \quad (136)$$

$$Vegfr3(t+1) = CSL(t) \quad (137)$$

$$VEGFR23(t+1) = Vegfr2(t) \wedge Vegfr3(t) \wedge (PECAM1(t) \vee VEGFAxxx(t) \vee VEGFC\_Dp(t)) \quad (138)$$

$$VEGFR33(t+1) = Vegfr3(t) \wedge (PECAM1(t) \vee VEGFC\_Dp(t) \vee VEGFC\_D(t)) \quad (139)$$

$$STAT3(t+1) = VEGFR22(t) \quad (140)$$

$$VegfA(t+1) = STAT3(t) \vee NFAT(t) \vee HIF1(t) \vee Lactate(t) \vee FOXO1(t) \vee KLF2(t) \quad (141)$$

$$NRP1(t+1) = (VEGFAxxx \vee VEGFC\_Dp) \wedge (\neg CSL \vee ETS) \quad (142)$$

3 SUPPLEMENTARY DATA

3.1 Update rule sensitivity

We estimated the sensitivity of each boolean rule by taking for each rule 500,000 random patterns of molecular activation and pairing them with a pattern that differs only in the activity of one molecule. The fraction of pairs, where the value of the molecule after update is different, constitutes our estimation of the sensitivity.

ANG1 = 0.015374, ANG2 = 0.018300, TIE2 = 0.015908, HIF1 = 0.015668, AMPK = 0.019604, Oxygen = 0.015726, SIRT1 = 0.019610, RAS = 0.011540, Integrin = 0.020086, KLF2 = 0.015780, VegfA = 0.002926, AMPATP = 0.015524, FOXO1 = 0.015602, FGF = 0.015350, MEK = 0.020836, ETS = 0.015620, ShearStress = 0.015632, VEcadherin = 0.018740, STAT3 = 0.015662, NRP1 = 0.023716, IGF = 0.015668, PLCg = 0.007694, Calcium = 0.011812, NFAT = 0.015768, FAK = 0.015666, PECAM1 = 0.007802, VEGFR22 = 0.019190, VEGFAxxxP = 0.015710, VEGFAxxxA = 0.008900, VEGFAxxx = 0.015716, NO = 0.011476, TSC = 0.015822, SRC = 0.007812, Vegfr2 = 0.019404, VEGFR23 = 0.016430, VEGFAxxxd = 0.015444, WNT5a = 0.015684, AP1 = 0.015478, AKT = 0.016062, PIP3 = 0.007992, p38MAPK = 0.011938, Vegfr3 = 0.015494, VEGFR33 = 0.019446, VEGFC_D = 0.015756, Bcatenin

= 0.015846, LEF1 = 0.019416, NRARP = 0.015766, NICD = 0.015372, HEY1 = 0.019726,SMAD2 = 0.011476,ALK5 = 0.015656,VEGFC_Dp = 0.015768,WNT7a = 0.015686,JAGa = 0.015412, NOTCH = 0.011916, SMAD6 = 0.015454, SMAD1 = 0.011536, ALK1 = 0.011590, BMP9 = 0.015786, DLL4a = 0.015782, JAGp = 0.015578, DLL4p = 0.015814, BMP10 = 0.015468, TGFB1 = 0.015790

4 SUPPLEMENTARY TABLES AND FIGURES

4.1 The effect of mutations that affect angiogenesis

Supplementary Table 1. Effect of mutations in the ANG-TIE signaling pathway (Eklund et al., 2017)

Gene	Reduced function	Augmented function
ANG1	In mice, deletion is lethal(E12.5); less complex vasculature, reduced number of ECs.	In mice, causes an increase in vessel number, diameter, branching and stability
ANG2	In mice, deletion sometimes is lethal (2 weeks) and defective vascular remodeling.	Decreased endothelial integrity, associated with numerous human diseases, including cancer, sepsis, infectious diseases, diabetes, atherosclerosis and tissue injury.
TIE1	In mice, deletion causes death due to extensive hemorrhage (E13.5); compromised capillary remodeling and EC survival.	NA
TIE2	Deletion is embryonic lethal at E9.510.5; causes glaucoma	Human venous malformations (VMs)
VEPTP	Stabilization of the endothelial cortical actin cytoskeleton via Tie2. In mice and zebrafish, defective EC polarization and lumen formation by stalk cells of newly-formed vascular sprouts via increased VE-cadherin phosphorylation and VEGFR2 activity. In a Tie2-deficient mouse background, VE-PTP inhibition promoted vascular leak via VE-cadherin.	Increased the integrity of adherence junctions in a lipo- polysaccharide induced murine systemic inflammation model

4.2 The simulated effect of gain and loss of function mutations

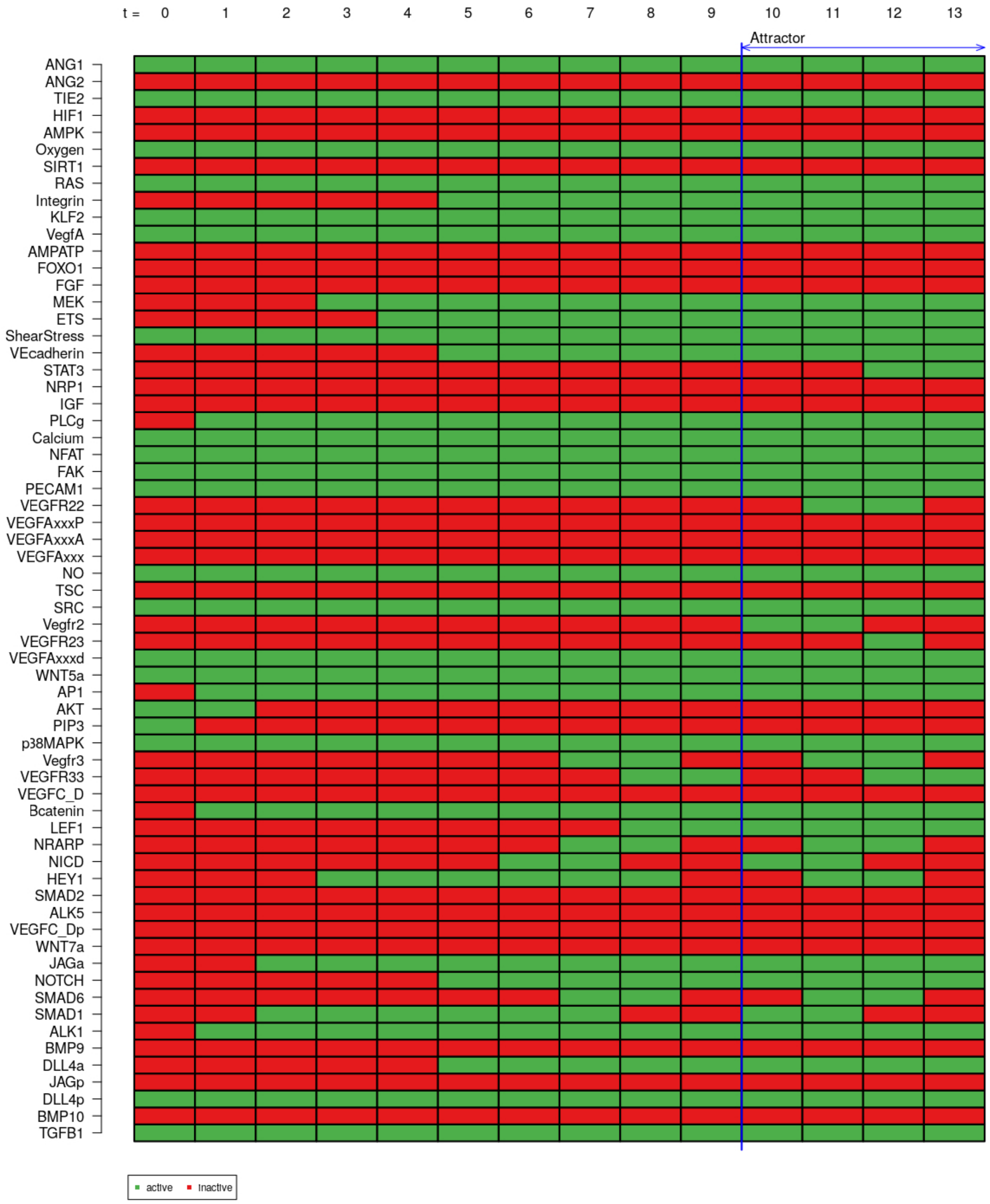

Supplementary Figure 1. Transition from Phalanx to Stalk EC behavior in our simplified model.

REFERENCES

Abhinand, C. S., Raju, R., Soumya, S. J., Arya, P. S., and Sudhakaran, P. R. (2016). VEGF-A/VEGFR2 signaling network in endothelial cells relevant to angiogenesis. *Journal of Cell Communication and Signaling* , 1–8doi:10.1007/s12079-016-0352-8

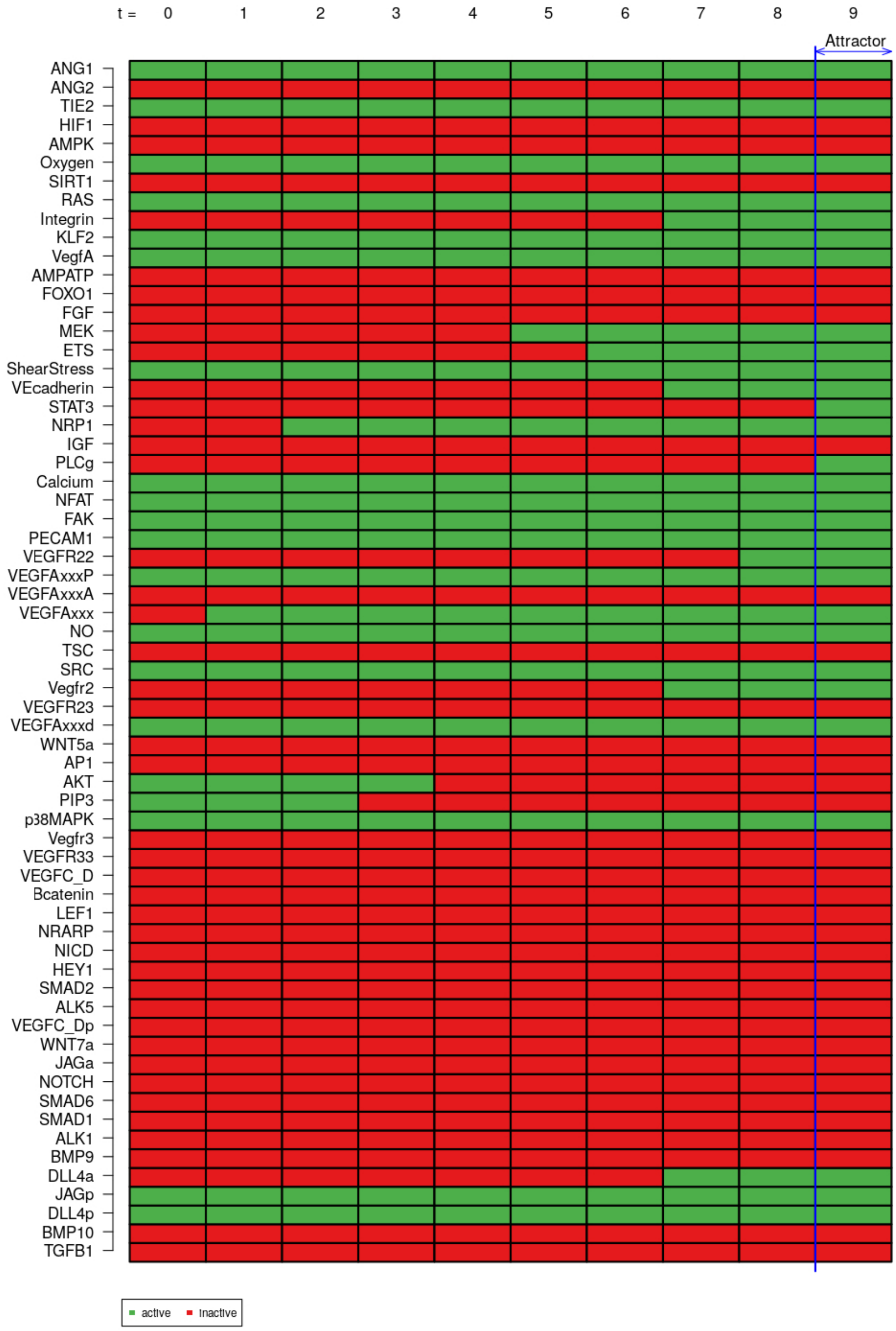

Supplementary Figure 2. Transition from Phalanx to Tip EC behavior in our simplified model.

Akhurst, R. J. (2004). TGFbeta signaling in health and disease. *Nature genetics* 36, 790–792. doi:10.1038/ng0804-790

Alavi, A., Hood, J. D., Frausto, R., Stupack, D. G., and Cheresh, D. A. (2003). Role of Raf in vascular protection from distinct apoptotic stimuli. *Science* 301, 94–96. doi:10.1126/science.1082015

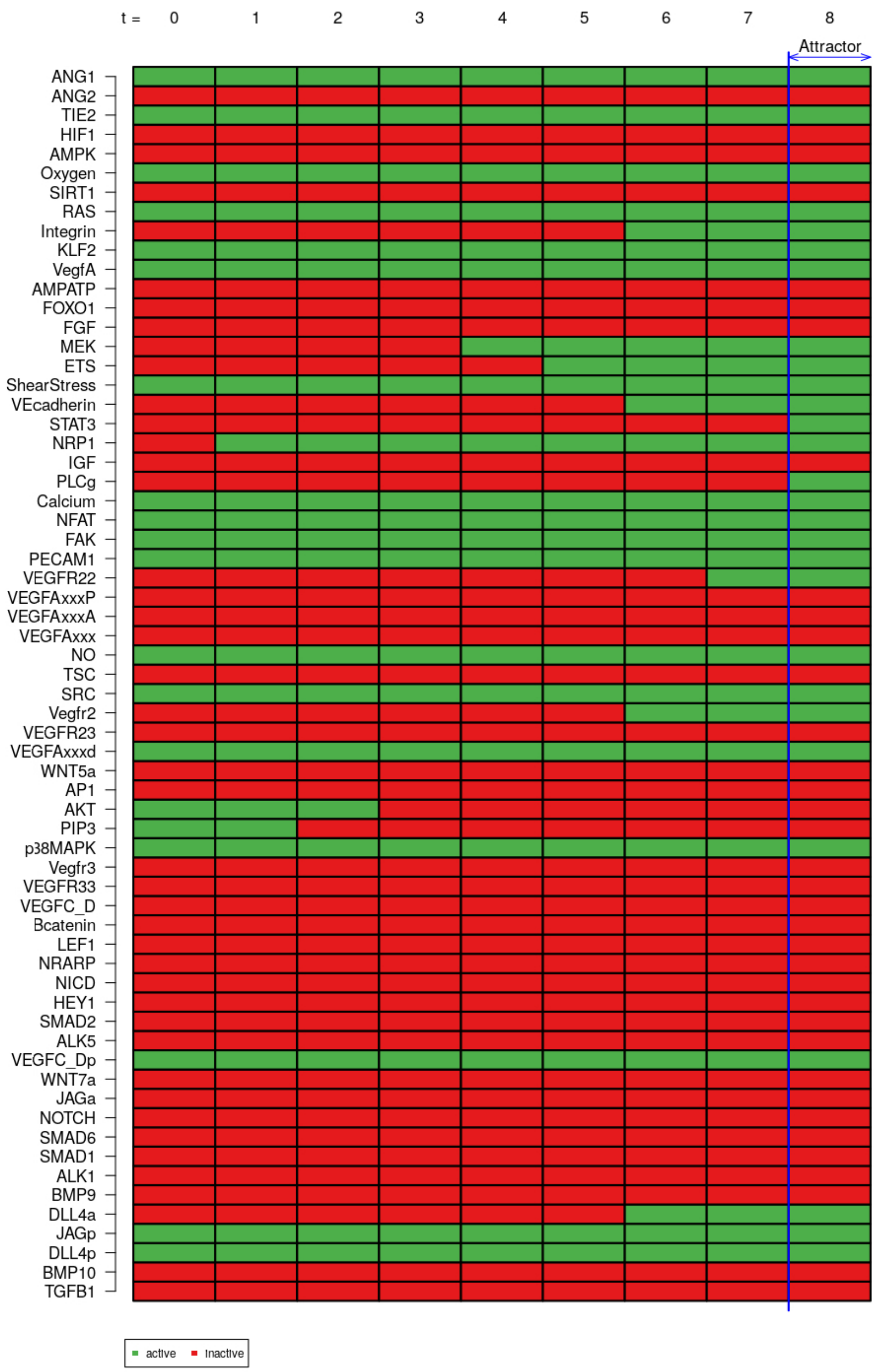

Supplementary Figure 3. Transition from Phalanx to Tip EC behavior with VEGFC or VEGFD in our simplified model.

Ampe, C. and Van Troys, M. (2017). Mammalian actins: Isoform-specific functions and diseases. In *The Actin Cytoskeleton*, ed. B. M. Jockusch (Cham: Springer International Publishing). 1–37. doi:10.1007/164_2016_43

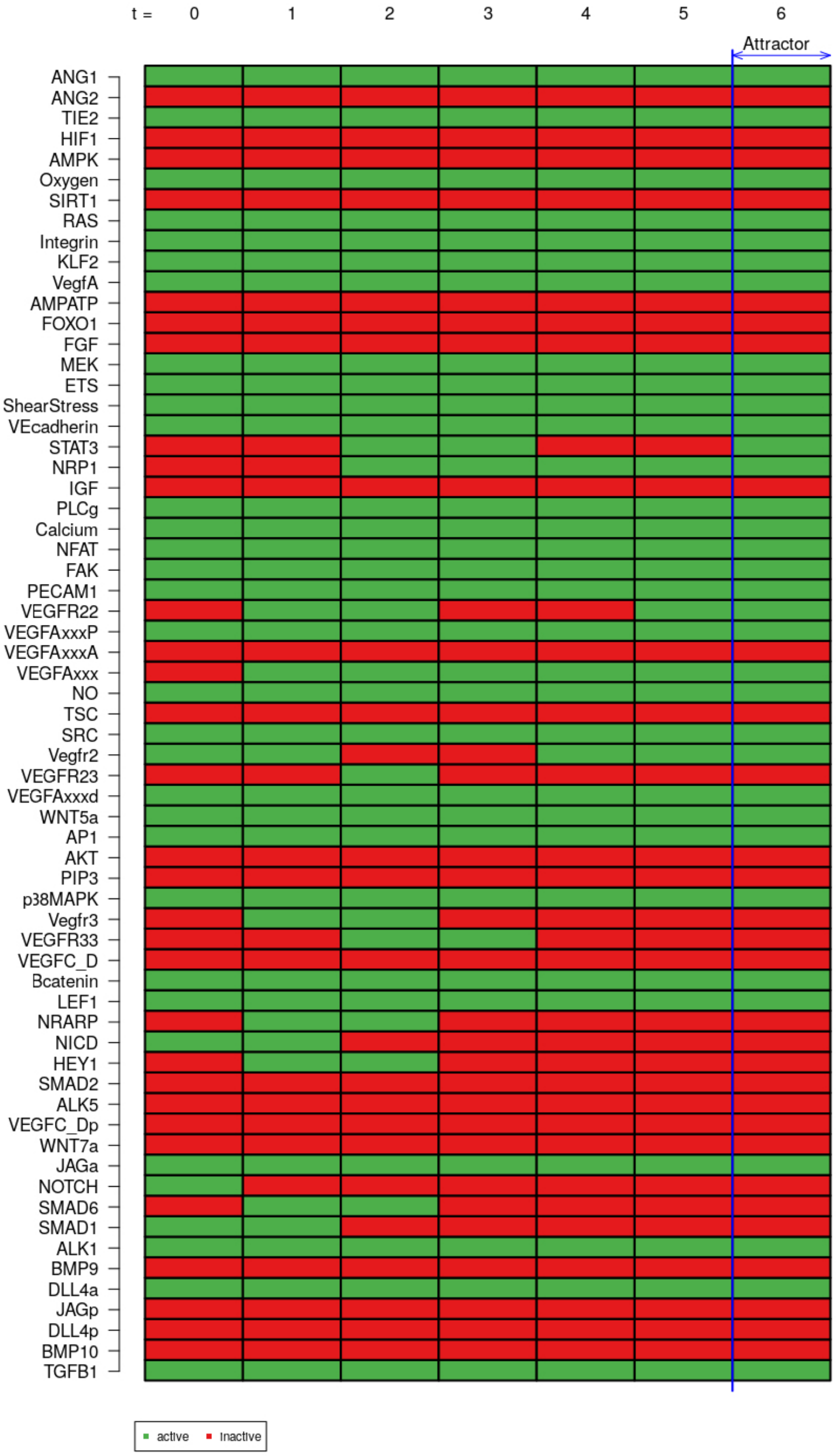

Supplementary Figure 4. Transition from Stalk to Phalanx EC behavior in our simplified model.

Andersson, E. R. and Lendahl, U. (2014). Therapeutic modulation of Notch signallingare we there yet? *Nature reviews Drug discovery* 13, 357–378. doi:10.1038/nrd4252

Arderiu, G., Espinosa, S., Peña, E., Aledo, R., and Badimon, L. (2014). Monocyte-secreted Wnt5a interacts with FZD5 in microvascular endothelial cells and induces angiogenesis through tissue factor signaling. *Journal of molecular cell biology* , mju036doi:10.1093/jmcb/mju036

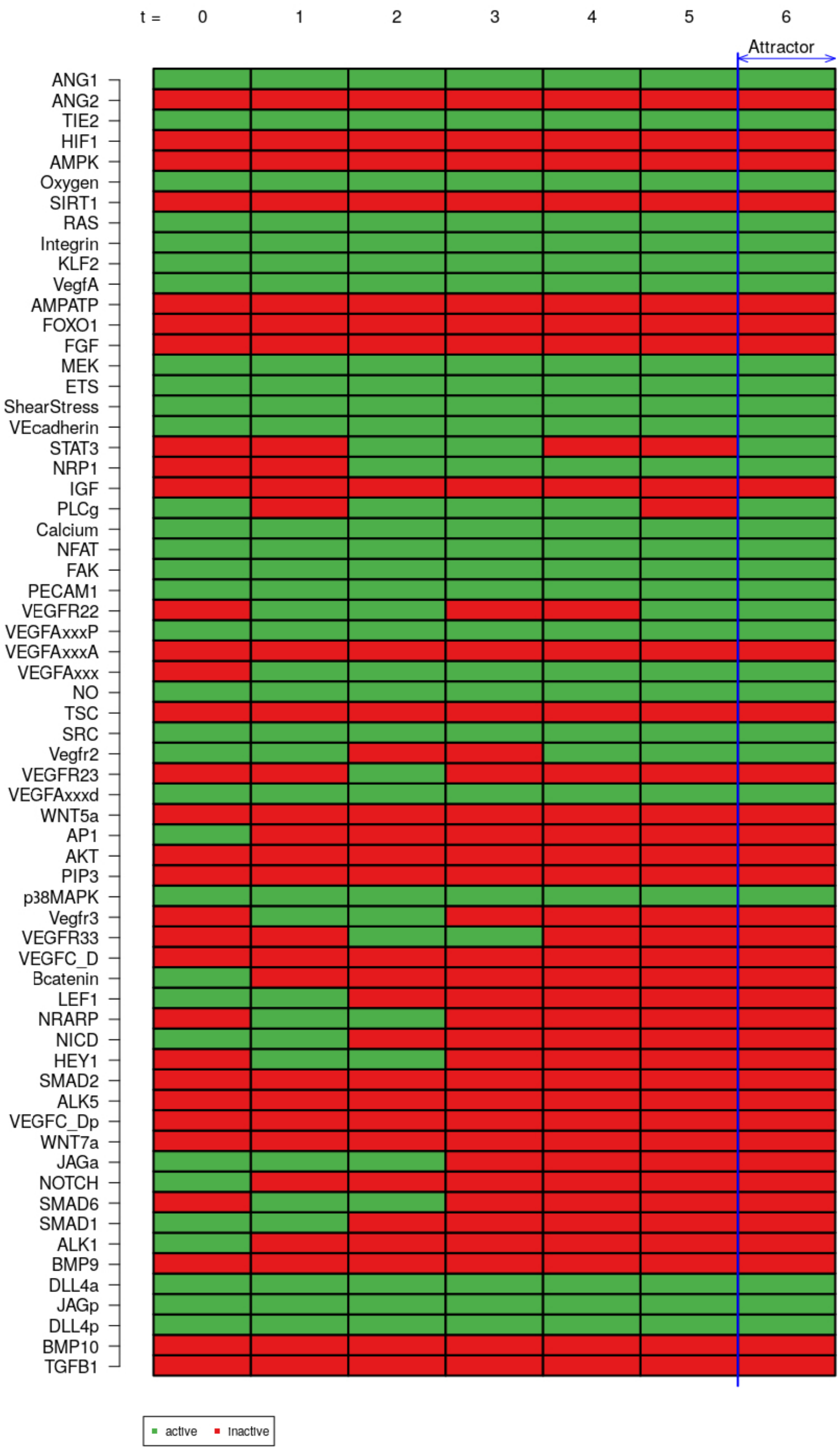

Supplementary Figure 5. Transition from Stalk to Tip EC behavior in our simplified model.

Aspalter, I. M., Gordon, E., Dubrac, A., Ragab, A., Narloch, J., Vizán, P., et al. (2015). Alk1 and Alk5 inhibition by Nrp1 controls vascular sprouting downstream of Notch. *Nature communications* 6. doi:10.1038/ncomms8264

Bayless, K. J. and Davis, G. E. (2002). The Cdc42 and Rac1 GTPases are required for capillary lumen formation in three-dimensional extracellular matrices. *Journal of cell science* 115, 1123–1136

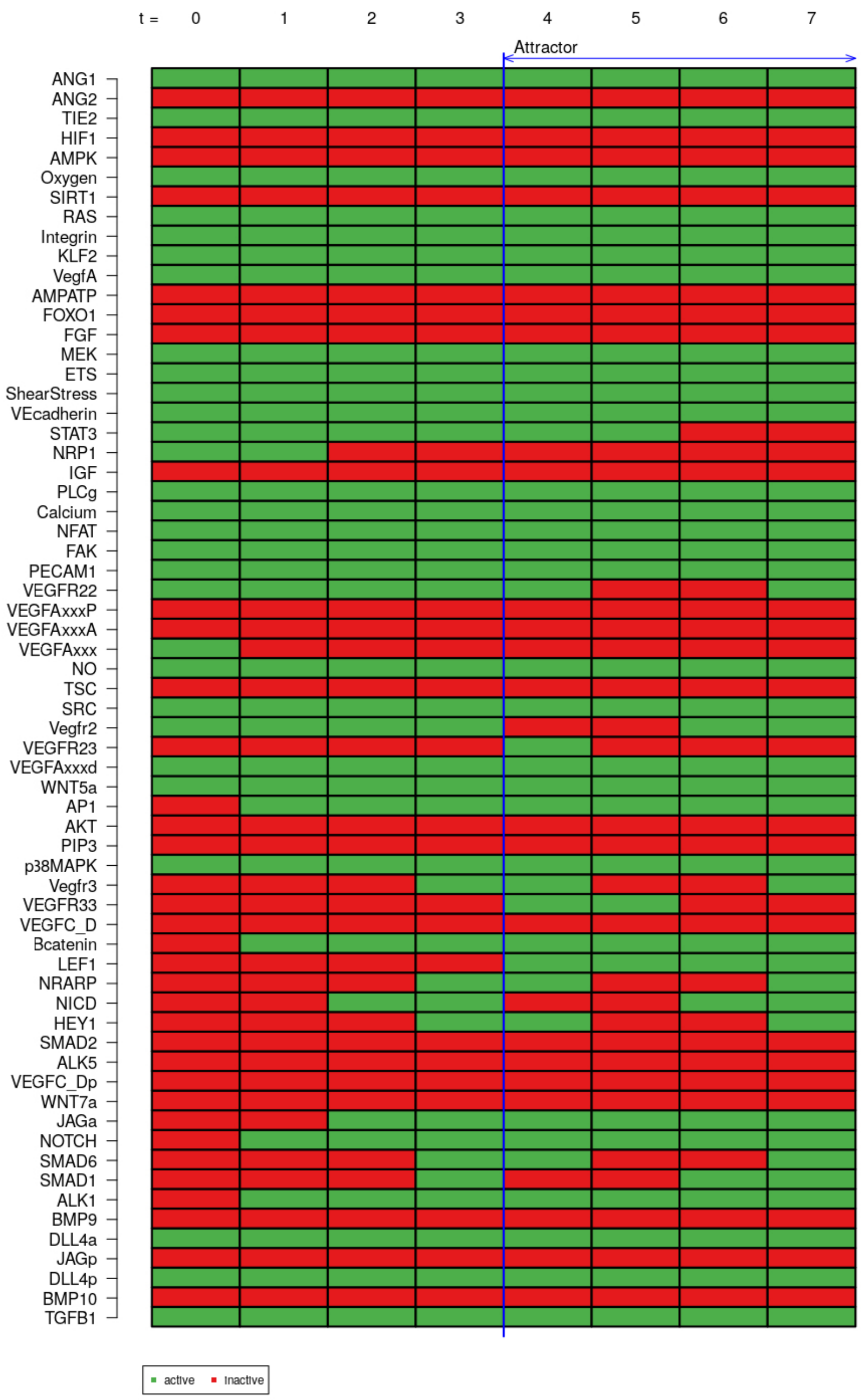

Supplementary Figure 6. Transition from Tip to Stalk EC behavior in our simplified model.

Betz, C., Lenard, A., Belting, H.-G., and Affolter, M. (2016). Cell behaviors and dynamics during angiogenesis. *Development* 143, 2249–2260. doi:10.1242/dev.135616

Blanco, R. and Gerhardt, H. (2013). VEGF and Notch in tip and stalk cell selection. *Cold Spring Harbor perspectives in medicine* 3, a006569. doi:10.1101/cshperspect.a006569

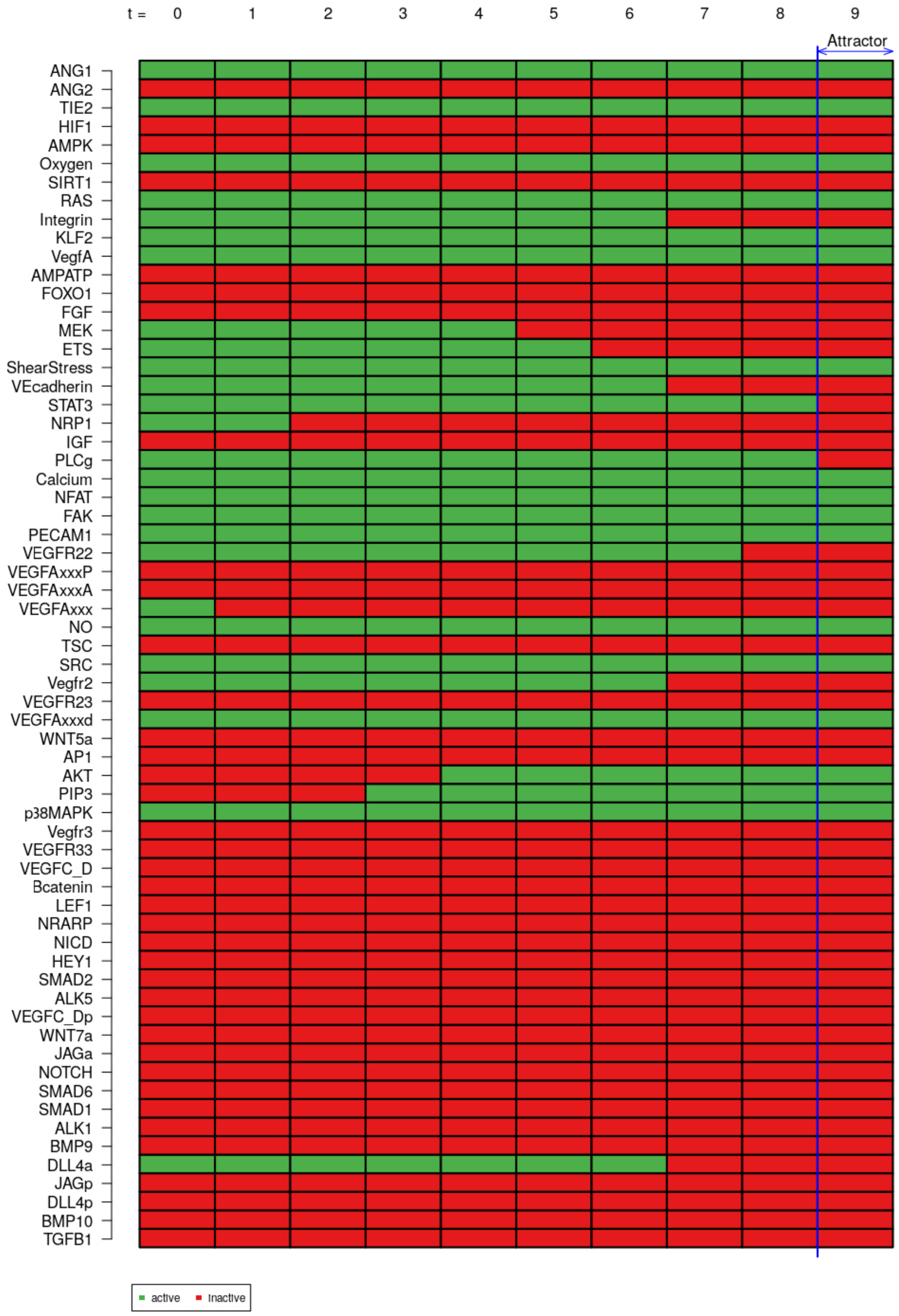

Supplementary Figure 7. Transition from Tip to Phalanx EC behavior in our simplified model.

Bower, N. I., Vogrin, A. J., Le Guen, L., Chen, H., Stacker, S. A., Achen, M. G., et al. (2017). Vegfd modulates both angiogenesis and lymphangiogenesis during zebrafish embryonic development. *Development* 144, 507–518. doi:10.1242/dev.146969

Brantjes, H., Roose, J., van de Wetering, M., and Clevers, H. (2001). All Tcf HMG box transcription factors interact with Groucho-related co-repressors. *Nucleic acids research* 29, 1410–1419

Cai, Z., Zhao, B., Deng, Y., Shangguan, S., Zhou, F., Zhou, W., et al. (2016). Notch signaling in cerebrovascular diseases (Review). *Molecular Medicine Reports* 14, 2883–2898. doi:10.3892/mmr.2016.5641

Cao, R., Eriksson, A., Kubo, H., Alitalo, K., Cao, Y., and Thyberg, J. (2004). Comparative Evaluation of FGF-2–, VEGF-A–, and VEGF-C–Induced Angiogenesis, Lymphangiogenesis, Vascular Fenestrations, and Permeability. *Circulation research* 94, 664–670. doi:10.1161/01.RES.0000118600.91698.BB

Caolo, V., Swennen, G., Chalaris, A., Wagenaar, A., Verbruggen, S., Rose-John, S., et al. (2015). ADAM10 and ADAM17 have opposite roles during sprouting angiogenesis. *Angiogenesis* 18, 13–22. doi:10.1007/s10456-014-9443-4

Carmeliet, P. and Jain, R. K. (2011). Molecular mechanisms and clinical applications of angiogenesis. *Nature* 473, 298–307. doi:10.1038/nature10144

Carter, J., Cherry, J., Williams, K., Turner, S., Bates, D., and Churchill, A. (2011). Splicing factor polymorphisms, the control of VEGF isoforms and association with angiogenic eye disease. *Current eye research* 36, 328–335. doi:10.3109/02713683.2010.548892

Cattelino, A., Liebner, S., Gallini, R., Zanetti, A., Balconi, G., Corsi, A., et al. (2003). The conditional inactivation of the β-catenin gene in endothelial cells causes a defective vascular pattern and increased vascular fragility. *The Journal of cell biology* 162, 1111–1122. doi:10.1083/jcb.200212157

Chamorro-Jorganes, A., Grande, M. T., Herranz, B., Jerkic, M., Griera, M., Gonzalez-Nuñez, M., et al. (2010). Targeted genomic disruption of H-Ras induces hypotension through a NO-cGMP-PKG pathway–dependent mechanism. *Hypertension* 56, 484–489. doi:10.1161/HYPERTENSIONAHA.110.152587

Chang, C.-P., Neilson, J. R., Bayle, J. H., Gestwicki, J. E., Kuo, A., Stankunas, K., et al. (2004). A field of myocardial-endocardial NFAT signaling underlies heart valve morphogenesis. *Cell* 118, 649–663. doi:10.1016/j.cell.2004.08.010

Chen, P.-Y., Qin, L., Tellides, G., and Simons, M. (2014). Fibroblast growth factor receptor 1 is a key inhibitor of TGF signaling in the endothelium. *Science Signaling* 7. doi:10.1126/scisignal.2005504

Chen, R., Dioum, E. M., Hogg, R. T., Gerard, R. D., and Garcia, J. A. (2011). Hypoxia increases sirtuin 1 expression in a hypoxia-inducible factor-dependent manner. *Journal of Biological Chemistry* 286, 13869–13878. doi:10.1074/jbc.M110.175414

Chistiakov, D. A., Orekhov, A. N., and Bobryshev, Y. V. (2016). Effects of shear stress on endothelial cells: go with the flow. *Acta Physiologica* doi:10.1111/apha.12725

Choi, E.-J., Kim, Y. H., Choe, S.-w., Tak, Y. G., Garrido-Martin, E. M., Chang, M., et al. (2013). Enhanced responses to angiogenic cues underlie the pathogenesis of hereditary hemorrhagic telangiectasia 2. *PloS one* 8, e63138. doi:10.1371/journal.pone.0063138

Chung, S.-H., Kim, S.-K., Kim, J. K., Yang, Y.-R., Suh, P.-G., and Chang, J.-S. (2010). A double point mutation in PCL-γ1 (y509a/f510a) enhances y783 phosphorylation and inositol phospholipid-hydrolyzing activity upon EGF stimulation. *Experimental & molecular medicine* 42, 216–222. doi:10.3858/emm.2010.42.3.023

Coon, B. G., Baeyens, N., Han, J., Budatha, M., Ross, T. D., Fang, J. S., et al. (2015). Intramembrane binding of VE-cadherin to VEGFR2 and VEGFR3 assembles the endothelial mechanosensory complex. *The Journal of cell biology* 208, 975–986. doi:10.1083/jcb.201408103

Covassin, L. D., Siekmann, A. F., Kacergis, M. C., Laver, E., Moore, J. C., Villefranc, J. A., et al. (2009). A genetic screen for vascular mutants in zebrafish reveals dynamic roles for Vegf/Plcg1 signaling during artery development. *Developmental biology* 329, 212–226. doi:10.1016/j.ydbio.2009.02.031

Craig, M. P. and Sumanas, S. (2016). ETS transcription factors in embryonic vascular development. *Angiogenesis* 19, 275–285. doi:10.1007/s10456-016-9511-z

Cruciat, C.-M. and Niehrs, C. (2013). Secreted and transmembrane wnt inhibitors and activators. *Cold Spring Harbor perspectives in biology* 5, a015081. doi:10.1101/cshperspect.a015081

Daneman, R., Agalliu, D., Zhou, L., Kuhnert, F., Kuo, C. J., and Barres, B. A. (2009). Wnt/β-catenin signaling is required for CNS, but not non-CNS, angiogenesis. *Proceedings of the National Academy of Sciences* 106, 641–646. doi:10.1073/pnas.0805165106

Dekker, R. J., Boon, R. A., Rondaij, M. G., Kragt, A., Volger, O. L., Elderkamp, Y. W., et al. (2006). KLF2 provokes a gene expression pattern that establishes functional quiescent differentiation of the endothelium. *Blood* 107, 4354–4363. doi:10.1182/blood-2005-08-3465

Delafontaine, P., Song, Y.-H., and Li, Y. (2004). Expression, regulation, and function of IGF-1, IGF-1R, and IGF-1 binding proteins in blood vessels. *Arteriosclerosis, Thrombosis, and Vascular Biology* 24, 435–444. doi:10.1161/01.ATV.0000105902.89459.09

Dummler, B., Tschopp, O., Hynx, D., Yang, Z.-Z., Dirnhofer, S., and Hemmings, B. A. (2006). Life with a single isoform of Akt: mice lacking Akt2 and Akt3 are viable but display impaired glucose homeostasis and growth deficiencies. *Molecular and cellular biology* 26, 8042–8051. doi:10.1128/MCB.00722-06

Eklund, L., Kangas, J., and Saharinen, P. (2017). Angiopoietin–Tie signalling in the cardiovascular and lymphatic systems. *Clinical Science* 131, 87–103. doi:10.1042/CS20160129

Erdmann, J., Stark, K., Esslinger, U. B., Rumpf, P. M., Koesling, D., de Wit, C., et al. (2013). Dysfunctional nitric oxide signalling increases risk of myocardial infarction. *Nature* 504, 432. doi:10.1038/nature12722

Faehling, M., Kroll, J., Föhr, K. J., Fellbrich, G., Mayr, U., Trischler, G., et al. (2002). Essential role of calcium in vascular endothelial growth factor A-induced signaling: mechanism of the antiangiogenic effect of carboxyamidotriazole. *The FASEB Journal* 16, 1805–1807. doi:10.1096/fj.01-0938fje

Fei, P., Zhang, Q., Huang, L., Xu, Y., Zhu, X., Tai, Z., et al. (2014). Identification of two novel LRP5 mutations in families with familial exudative vitreoretinopathy. *Molecular vision* 20, 395

Ferrara, N. (2010). Binding to the extracellular matrix and proteolytic processing: two key mechanisms regulating vascular endothelial growth factor action. *Molecular biology of the cell* 21, 687–690. doi:10.1091/mbc.E09-07-0590

Ferrara, N., Carver-Moore, K., Chen, H., Dowd, M., et al. (1996). Heterozygous embryonic lethality induced by targeted inactivation of the VEGF gene. *Nature* 380, 439. doi:10.1038/380439a0

Ferrara, N., Gerber, H.-P., and LeCouter, J. (2003). The biology of VEGF and its receptors. *Nature medicine* 9, 669–676. doi:10.1038/nm0603-669

Fischer, A., Schumacher, N., Maier, M., Sendtner, M., and Gessler, M. (2004). The notch target genes Hey1 and Hey2 are required for embryonic vascular development. *Genes & development* 18, 901–911. doi:10.1101/gad.291004

Fischer, C., Mazzone, M., Jonckx, B., and Carmeliet, P. (2008). FLT1 and its ligands VEGFB and PlGF: drug targets for anti-angiogenic therapy? *Nature Reviews Cancer* 8, 942–956. doi:10.1038/nrc2524

Fish, J. E., Santoro, M. M., Morton, S. U., Yu, S., Yeh, R.-F., Wythe, J. D., et al. (2008). mir-126 regulates angiogenic signaling and vascular integrity. *Developmental cell* 15, 272–284. doi:10.1016/j.devcel.2008.07.008

Fong, G.-H., Rossant, J., Gertsenstein, M., and Breitman, M. L. (1995). Role of the Flt-1 receptor tyrosine kinase in regulating the assembly of vascular endothelium. *Nature* 376, 66. doi:10.1038/376066a0

Forsythe, J. A., Jiang, B.-H., Iyer, N. V., Agani, F., Leung, S. W., Koos, R. D., et al. (1996). Activation of vascular endothelial growth factor gene transcription by hypoxia-inducible factor 1. *Molecular and cellular biology* 16, 4604–4613. doi:10.1128/MCB.16.9.4604

Fraisl, P., Mazzone, M., Schmidt, T., and Carmeliet, P. (2009). Regulation of angiogenesis by oxygen and metabolism. *Developmental cell* 16, 167–179. doi:10.1016/j.devcel.2009.01.003

Franco, C. A., Liebner, S., and Gerhardt, H. (2009). Vascular morphogenesis: a wnt for every vessel? *Current opinion in genetics & development* 19, 476–483. doi:10.1016/j.gde.2009.09.004

Frémin, C., Saba-El-Leil, M. K., Lévesque, K., Ang, S.-L., and Meloche, S. (2015). Functional redundancy of ERK1 and ERK2 MAP kinases during development. *Cell reports* 12, 913–921. doi:10.1016/j.celrep.2015.07.011

Frye, M., Dierkes, M., Küppers, V., Vockel, M., Tomm, J., Zeuschner, D., et al. (2015). Interfering with VE-PTP stabilizes endothelial junctions in vivo via Tie-2 in the absence of VE-cadherin. *The Journal of experimental medicine* 212, 2267–2287. doi:10.1084/jem.20150718

Fukumura, D., Gohongi, T., Kadambi, A., Izumi, Y., Ang, J., Yun, C.-O., et al. (2001). Predominant role of endothelial nitric oxide synthase in vascular endothelial growth factor-induced angiogenesis and vascular permeability. *Proceedings of the National Academy of Sciences* 98, 2604–2609. doi:10.1073/pnas.041359198

Funahashi, Y., Shawber, C. J., Vorontchikhina, M., Sharma, A., Outtz, H. H., and Kitajewski, J. (2010). Notch regulates the angiogenic response via induction of VEGFR-1. *Journal of angiogenesis research* 2, 1. doi:10.1186/2040-2384-2-3

Furuyama, T., Kitayama, K., Shimoda, Y., Ogawa, M., Sone, K., Yoshida-Araki, K., et al. (2004). Abnormal angiogenesis in Foxo1 (Fkhr)-deficient mice. *Journal of Biological Chemistry* 279, 34741–34749. doi:10.1074/jbc.M314214200

Gerber, H.-P., Condorelli, F., Park, J., and Ferrara, N. (1997). Differential transcriptional regulation of the two vascular endothelial growth factor receptor genes Flt-1, but not Flk-1/KDR, is up-regulated by hypoxia. *Journal of Biological Chemistry* 272, 23659–23667. doi:10.1074/jbc.272.38.23659

Geudens, I. and Gerhardt, H. (2011). Coordinating cell behaviour during blood vessel formation. *Development* 138, 4569–4583. doi:10.1242/dev.062323

Giannotta, M., Trani, M., and Dejana, E. (2013). VE-cadherin and endothelial adherens junctions: active guardians of vascular integrity. *Developmental cell* 26, 441–454. doi:10.1016/j.devcel.2013.08.020

Gilmour, D. (2015). Familial exudative vitreoretinopathy and related retinopathies. *Eye* 29, 1–14. doi:10.1038/eye.2014.70

Giroux, S., Tremblay, M., Bernard, D., Cardin-Girard, J., Aubry, S., Larouche, L., et al. (1999). Embryonic death of Mek1-deficient mice reveals a role for this kinase in angiogenesis in the labyrinthine region of the placenta. *Current Biology* 9, 369–376. doi:10.1016/S0960-9822(99)80164-X

Glass, D. S., Jin, X., and Riedel-Kruse, I. H. (2016). Signaling delays preclude defects in lateral inhibition patterning. *Physical review letters* 116, 128102. doi:10.1103/PhysRevLett.116.128102

Glueck, C. J., Munjal, J., Khan, A., Umar, M., and Wang, P. (2010). Endothelial nitric oxide synthase T-786C mutation, a reversible etiology of Prinzmetal's angina pectoris. *The American journal of cardiology* 105, 792–796. doi:10.1016/j.amjcard.2009.10.062

Goorden, S. M., Hoogeveen-Westerveld, M., Cheng, C., van Woerden, G. M., Mozaffari, M., Post, L., et al. (2011). Rheb is essential for murine development. *Molecular and cellular biology* 31, 1672–1678. doi:10.1128/MCB.00985-10

Gordon, E. J., Fukuhara, D., Weström, S., Padhan, N., Sjöström, E. O., van Meeteren, L., et al. (2016). The endothelial adaptor molecule TSAd is required for VEGF-induced angiogenic sprouting through junctional c-Src activation. *Sci. Signal.* 9, ra72–ra72. doi:10.1126/scisignal.aad9256

Graef, I. A., Chen, F., Chen, L., Kuo, A., and Crabtree, G. R. (2001). Signals transduced by Ca 2+/calcineurin and NFATc3/c4 pattern the developing vasculature. *Cell* 105, 863–875. doi:10.1016/S0092-8674(01)00396-8

Guangqi, E., Cao, Y., Bhattacharya, S., Dutta, S., Wang, E., and Mukhopadhyay, D. (2012). Endogenous vascular endothelial growth factor-A (VEGF-A) maintains endothelial cell homeostasis by regulating VEGF receptor-2 transcription. *Journal of Biological Chemistry* 287, 3029–3041. doi:10.1074/jbc.M111.29398

Guo, J., Li, Y., Ren, Y.-H., Sun, Z., Dong, J., Yan, H., et al. (2016). Mutant LRP6 Impairs Endothelial Cell Functions Associated with Familial Normolipidemic Coronary Artery Disease. *International Journal of Molecular Sciences* 17, 1173. doi:10.3390/ijms17071173

Hamada, K., Sasaki, T., Koni, P. A., Natsui, M., Kishimoto, H., Sasaki, J., et al. (2005). The PTEN/PI3K pathway governs normal vascular development and tumor angiogenesis. *Genes & development* 19, 2054–2065. doi:10.1101/gad.1308805

Han, J., Hubbard, B. P., Lee, J., Montagna, C., Lee, H.-W., Sinclair, D. A., et al. (2013). Analysis of 41 cancer cell lines reveals excessive allelic loss and novel mutations in the SIRT1 gene. *Cell Cycle* 12, 263–270. doi:10.4161/cc.23056

Hanai, J.-i., Dhanabal, M., Karumanchi, S. A., Albanese, C., Waterman, M., Chan, B., et al. (2002). Endostatin causes G1 arrest of endothelial cells through inhibition of cyclin D1. *Journal of biological chemistry* 277, 16464–16469. doi:10.1074/jbc.M112274200

Harper, S. J. and Bates, D. O. (2008). VEGF-A splicing: the key to anti-angiogenic therapeutics? *Nature Reviews Cancer* 8, 880–887. doi:10.1038/nrc2505

Hay, N. (2005). The Akt-mTOR tango and its relevance to cancer. *Cancer cell* 8, 179–183. doi:10.1016/j.ccr.2005.08.008

Hayashi, H. and Kume, T. (2008). Foxc transcription factors directly regulate Dll4 and Hey2 expression by interacting with the VEGF-Notch signaling pathways in endothelial cells. *PloS one* 3, e2401. doi:10.1371/journal.pone.0002401

Hayashi, M., Majumdar, A., Li, X., Adler, J., Sun, Z., Vertuani, S., et al. (2013). VE-PTP regulates VEGFR2 activity in stalk cells to establish endothelial cell polarity and lumen formation. *Nature communications* 4, 1672. doi:10.1038/ncomms2683

Hellström, M., Phng, L.-K., Hofmann, J. J., Wallgard, E., Coultas, L., Lindblom, P., et al. (2007). Dll4 signalling through Notch1 regulates formation of tip cells during angiogenesis. *Nature* 445, 776–780. doi:10.1038/nature05571

Herbert, S. P. and Stainier, D. Y. (2011). Molecular control of endothelial cell behaviour during blood vessel morphogenesis. *Nature reviews Molecular cell biology* 12, 551–564. doi:10.1038/nrm3176

Hoang, M. V., Nagy, J. A., and Senger, D. R. (2011). Active Rac1 improves pathologic VEGF neovessel architecture and reduces vascular leak: mechanistic similarities with angiopoietin-1. *Blood* 117, 1751–1760. doi:10.1182/blood-2010-05-286831

Hoang, M. V., Whelan, M. C., and Senger, D. R. (2004). Rho activity critically and selectively regulates endothelial cell organization during angiogenesis. *Proceedings of the National Academy of Sciences* 101, 1874–1879. doi:10.1073/pnas.0308525100

Hodivala-Dilke, K. (2008). $\alpha v \beta 3$ integrin and angiogenesis: a moody integrin in a changing environment. *Current opinion in cell biology* 20, 514–519. doi:10.1016/j.ceb.2008.06.007

Holderfield, M. T., Anderson, A. M. H., Kokubo, H., Chin, M. T., Johnson, R. L., and Hughes, C. C. (2006). HESR1/CHF2 suppresses VEGFR2 transcription independent of binding to E-boxes. *Biochemical and biophysical research communications* 346, 637–648. doi:10.1016/j.bbrc.2006.05.177

Hong, C. C., Peterson, Q. P., Hong, J.-Y., and Peterson, R. T. (2006). Artery/vein specification is governed by opposing phosphatidylinositol-3 kinase and MAP kinase/ERK signaling. *Current Biology* 16, 1366–1372. doi:10.1016/j.cub.2006.05.046

Hu, J., Dong, A., Fernandez-Ruiz, V., Shan, J., Kawa, M., Martínez-Ansó, E., et al. (2009). Blockade of Wnt signaling inhibits angiogenesis and tumor growth in hepatocellular carcinoma. *Cancer research* 69, 6951–6959. doi:10.1158/0008-5472.CAN-09-0541

Huang, Y., Miao, Z., Hu, Y., Yuan, Y., Zhou, Y., Wei, L., et al. (2017). Baicalein reduces angiogenesis in the inflammatory microenvironment via inhibiting the expression of AP-1. *Oncotarget* 8, 883. doi:10.18632/oncotarget.13669

Ito, T.-K., Ishii, G., Saito, S., Yano, K., Hoshino, A., Suzuki, T., et al. (2009). Degradation of soluble VEGF receptor-1 by MMP-7 allows VEGF access to endothelial cells. *Blood* 113, 2363–2369. doi:10.1182/blood-2008-08-172742

Jadrich, J. L., O'Connor, M. B., and Coucouvanis, E. (2006). The TGFβ activated kinase TAK1 regulates vascular development in vivo. *Development* 133, 1529–1541. doi:10.1242/dev.02333

Jin, Y., Kaluza, D., and Jakobsson, L. (2014). VEGF, Notch and TGFβ/BMPs in regulation of sprouting angiogenesis and vascular patterning. *Biochemical Society Transactions* 42, 1576–1583. doi:10.1042/BST20140231

Jin, Z.-G., Ueba, H., Tanimoto, T., Lungu, A. O., Frame, M. D., and Berk, B. C. (2003). Ligand-independent activation of vascular endothelial growth factor receptor 2 by fluid shear stress regulates activation of endothelial nitric oxide synthase. *Circulation research* 93, 354–363. doi:10.1161/01.RES.0000089257.94002.96

Jinnin, M., Medici, D., Park, L., Limaye, N., Liu, Y., Boscolo, E., et al. (2008). Suppressed NFAT-dependent VEGFR1 expression and constitutive VEGFR2 signaling in infantile hemangioma. *Nature medicine* 14, 1236–1246. doi:10.1038/nm.1877

Ju, R., Cirone, P., Lin, S., Griesbach, H., Slusarski, D. C., and Crews, C. M. (2010). Activation of the planar cell polarity formin DAAM1 leads to inhibition of endothelial cell proliferation, migration, and angiogenesis. *Proceedings of the National Academy of Sciences* 107, 6906–6911. doi:10.1073/pnas.1001075107

Kappas, N. C., Zeng, G., Chappell, J. C., Kearney, J. B., Hazarika, S., Kallianos, K. G., et al. (2008). The VEGF receptor flt-1 spatially modulates Flk-1 signaling and blood vessel branching. *The Journal of cell biology* 181, 847–858. doi:10.1083/jcb.200709114

Karar, J. and Maity, A. (2011). PI3K/AKT/mTOR pathway in angiogenesis. *Frontiers in molecular neuroscience* 4, 51. doi:10.3389/fnmol.2011.00051

Keller, A., Westenberger, A., Sobrido, M. J., García-Murias, M., Domingo, A., Sears, R. L., et al. (2013). Mutations in the gene encoding PDGF-B cause brain calcifications in humans and mice. *Nature genetics* 45, 1077–1082. doi:10.1038/ng.2723

Kerr, B. A., West, X. Z., Kim, Y.-W., Zhao, Y., Tischenko, M., Cull, R. M., et al. (2016). Stability and function of adult vasculature is sustained by Akt/Jagged1 signalling axis in endothelium. *Nature communications* 7. doi:10.1038/ncomms10960

Kilarski, W. W., Jura, N., and Gerwins, P. (2003). Inactivation of Src family kinases inhibits angiogenesis in vivo: implications for a mechanism involving organization of the actin cytoskeleton. *Experimental cell research* 291, 70–82. doi:10.1016/S0014-4827(03)00374-4

Kim, H.-S., Skurk, C., Thomas, S. R., Bialik, A., Suhara, T., Kureishi, Y., et al. (2002). Regulation of angiogenesis by glycogen synthase kinase-3β. *Journal of Biological Chemistry* 277, 41888–41896. doi:10.1074/jbc.M206657200

Kim, Y., Nam, H. J., Lee, J., Park, D. Y., Kim, C., Yu, Y. S., et al. (2016). Methylation-dependent regulation of HIF-1α stability restricts retinal and tumour angiogenesis. *Nature communications* 7. doi:10.1038/ncomms10347

Kong, D., Zhan, Y., Liu, Z., Ding, T., Li, M., Yu, H., et al. (2016). SIRT1-mediated ERβ suppression in the endothelium contributes to vascular aging. *Aging Cell* 15, 1092–1102. doi:10.1111/acel.12515

Korhonen, E. A., Lampinen, A., Giri, H., Anisimov, A., Kim, M., Allen, B., et al. (2016). Tie1 controls angiopoietin function in vascular remodeling and inflammation. *The Journal of Clinical Investigation* 126, 3495–3510. doi:10.1172/JCI84923

Korn, C. and Augustin, H. G. (2015). Mechanisms of vessel pruning and regression. *Developmental cell* 34, 5–17. doi:10.1016/j.devcel.2015.06.004

Krebs, L. T., Xue, Y., Norton, C. R., Shutter, J. R., Maguire, M., Sundberg, J. P., et al. (2000). Notch signaling is essential for vascular morphogenesis in mice. *Genes & development* 14, 1343–1352. doi:10.1101/gad.14.11.1343

Kumar, V. S., Binu, S., Soumya, S., Haritha, K., and Sudhakaran, P. (2014). Regulation of vascular endothelial growth factor by metabolic context of the cell. *Glycoconjugate journal* 31, 427–434. doi:10.1007/s10719-014-9547-5

Kutys, M. L. and Chen, C. S. (2016). Forces and mechanotransduction in 3D vascular biology. *Current opinion in cell biology* 42, 73–79. doi:10.1161/01.RES.0000089257.94002.96

Laemmle, A., Lechleiter, A., Roh, V., Schwarz, C., Portmann, S., Furer, C., et al. (2012). Inhibition of SIRT1 impairs the accumulation and transcriptional activity of HIF-1α protein under hypoxic conditions. *PLoS One* 7, e33433. doi:10.1371/journal.pone.0033433

LaGory, E. L. and Giaccia, A. J. (2016). The ever-expanding role of HIF in tumour and stromal biology. *Nature cell biology* 18, 356–365. doi:10.1038/ncb3330

Lamar, E., Deblandre, G., Wettstein, D., Gawantka, V., Pollet, N., Niehrs, C., et al. (2001). Nrarp is a novel intracellular component of the Notch signaling pathway. *Genes & development* 15, 1885–1899. doi:10.1101/gad.908101

Leppänen, V.-M., Jeltsch, M., Anisimov, A., Tvorogov, D., Aho, K., Kalkkinen, N., et al. (2011). Structural determinants of vascular endothelial growth factor-D receptor binding and specificity. *Blood* 117, 1507–1515. doi:10.1182/blood-2010-08-301549

Leppänen, V.-M., Prota, A. E., Jeltsch, M., Anisimov, A., Kalkkinen, N., Strandin, T., et al. (2010). Structural determinants of growth factor binding and specificity by VEGF receptor 2. *Proceedings of the National Academy of Sciences* 107, 2425–2430. doi:10.1073/pnas.0914318107

Lertkiatmongkol, P., Liao, D., Mei, H., Hu, Y., and Newman, P. J. (2016). Endothelial functions of platelet/endothelial cell adhesion molecule-1 (CD31). *Current opinion in hematology* 23, 253–259. doi:10.1097/MOH.0000000000000239

Li, J.-L. and Harris, A. L. (2005). Notch signaling from tumor cells: a new mechanism of angiogenesis. *Cancer cell* 8, 1–3. doi:10.1016/j.ccr.2005.06.013

Li, L., Saliba, P., Reischl, S., Marti, H. H., and Kunze, R. (2016). Neuronal deficiency of HIF prolyl 4-hydroxylase 2 in mice improves ischemic stroke recovery in an HIF dependent manner. *Neurobiology of disease* 91, 221–235. doi:10.1016/j.nbd.2016.03.018

Limbourg, F. P., Takeshita, K., Radtke, F., Bronson, R. T., Chin, M. T., and Liao, J. K. (2005). Essential role of endothelial Notch1 in angiogenesis. *Circulation* 111, 1826–1832. doi:10.1161/01.CIR.0000160870.93058.DD

Linder, M., Duplaa, C., Couffinhal, T., and Malandain, G. (2015). Quantitative comparison of micro-vasculatures. In *Biomedical Imaging (ISBI), 2015 IEEE 12th International Symposium on* (IEEE), 516–519. doi:10.1109/ISBI.2015.7163924

Liu, J., Stevens, J., Matsunami, N., and White, R. L. (2004). Targeted degradation of β-catenin by chimeric F-box fusion proteins. *Biochemical and biophysical research communications* 313, 1023–1029. doi:10.18632/oncotarget.10134

Liu, J., You, P., Chen, G., Fu, X., Zeng, X., Wang, C., et al. (2016). Hyperactivated FRS2α-mediated signaling in prostate cancer cells promotes tumor angiogenesis and predicts poor clinical outcome of patients. *Oncogene* 35, 1750–1759. doi:10.1038/onc.2015.239

Liu, Z.-J., Shirakawa, T., Li, Y., Soma, A., Oka, M., Dotto, G. P., et al. (2003). Regulation of Notch1 and Dll4 by vascular endothelial growth factor in arterial endothelial cells: implications for modulating arteriogenesis and angiogenesis. *Molecular and cellular biology* 23, 14–25. doi:10.1128/MCB.23.1.14-25.2003

Luttun, A., Tjwa, M., Moons, L., Wu, Y., Angelillo-Scherrer, A., Liao, F., et al. (2002). Revascularization of ischemic tissues by PlGF treatment, and inhibition of tumor angiogenesis, arthritis and atherosclerosis by anti-Flt1. *Nature medicine* 8, 831–840. doi:10.1038/nm731

Ma, A., Wang, L., Gao, Y., Chang, Z., Peng, H., Zeng, N., et al. (2014). Tsc1 deficiency-mediated mTOR hyperactivation in vascular endothelial cells causes angiogenesis defects and embryonic lethality. *Human molecular genetics* 23, 693–705. doi:10.1093/hmg/ddt456

Ma, J., Xue, Y., Liu, W., Yue, C., Bi, F., Xu, J., et al. (2013). Role of activated rac1/cdc42 in mediating endothelial cell proliferation and tumor angiogenesis in breast cancer. *PloS one* 8, e66275. doi:10.1371/journal.pone.0066275

Mac Gabhann, F. and Popel, A. S. (2007). Dimerization of VEGF receptors and implications for signal transduction: a computational study. *Biophysical chemistry* 128, 125–139. doi:10.1016/j.bpc.2007.03.010

Manalo, D. J., Rowan, A., Lavoie, T., Natarajan, L., Kelly, B. D., Shui, Q. Y., et al. (2005). Transcriptional regulation of vascular endothelial cell responses to hypoxia by HIF-1. *Blood* 105, 659–669. doi:10.1182/blood-2004-07-2958

Mannell, H., Hellwig, N., Gloe, T., Plank, C., Sohn, H.-Y., Groesser, L., et al. (2008). Inhibition of the tyrosine phosphatase SHP-2 suppresses angiogenesis in vitro and in vivo. *Journal of vascular research* 45, 153–163. doi:10.1159/000110081

Marcu, R., Kotha, S., Zhi, Z., Qin, W., Neeley, C. K., Wang, R., et al. (2015). Mitochondrial Permeability Transition Pore Regulates Endothelial Bioenergetics and Angiogenesis. *Circulation research* , CIRCRESAHA–114doi:10.1161/CIRCRESAHA.116.304881

Maring, J., van Meeteren, L., Goumans, M., and ten Dijke, P. (2016). Interrogating TGF-β function and regulation in endothelial cells. *TGF-β Signaling: Methods and Protocols* , 193–203doi:10.1007/978-1-4939-2966-5_11

Masckauchán, T. N. H., Agalliu, D., Vorontchikhina, M., Ahn, A., Parmalee, N. L., Li, C.-M., et al. (2006). Wnt5a signaling induces proliferation and survival of endothelial cells in vitro and expression of MMP-1 and Tie-2. *Molecular biology of the cell* 17, 5163–5172. doi:10.1091/mbc.E06-04-0320

Meili, R., Sasaki, A. T., and Firtel, R. A. (2005). Rho rocks PTEN. *Nature Cell Biology* 7, 334–335. doi:10.1038/ncb0405-334

Minami, Y., Oishi, I., Endo, M., and Nishita, M. (2010). Ror-family receptor tyrosine kinases in noncanonical Wnt signaling: Their implications in developmental morphogenesis and human diseases. *Developmental Dynamics* 239, 1–15. doi:10.1002/dvdy.21991

Moriya, J. and Ferrara, N. (2015). Inhibition of protein kinase C enhances angiogenesis induced by platelet-derived growth factor C in hyperglycemic endothelial cells. *Cardiovascular diabetology* 14, 19. doi:10.1186/s12933-015-0180-9

Morris, B. J. (2013). Seven sirtuins for seven deadly diseases of aging. *Free Radical Biology and Medicine* 56, 133–171. doi:10.1016/j.freeradbiomed.2012.10.525

Mouillesseaux, K. P., Wiley, D. S., Saunders, L. M., Wylie, L. A., Kushner, E. J., Chong, D. C., et al. (2016). Notch regulates BMP responsiveness and lateral branching in vessel networks via SMAD6. *Nature Communications* 7. doi:doi:10.1038/ncomms13247

Mudgett, J. S., Ding, J., Guh-Siesel, L., Chartrain, N. A., Yang, L., Gopal, S., et al. (2000). Essential role for p38α mitogen-activated protein kinase in placental angiogenesis. *Proceedings of the National Academy of Sciences* 97, 10454–10459. doi:10.1073/pnas.180316397

Murakami, M., Nguyen, L. T., Hatanaka, K., Schachterle, W., Chen, P.-Y., Zhuang, Z. W., et al. (2011). FGF-dependent regulation of VEGF receptor 2 expression in mice. *The Journal of clinical investigation* 121, 2668–2678. doi:10.1172/JCI44762

Newman, A. C. and Hughes, C. C. (2012). Macrophages and angiogenesis: a role for Wnt signaling. *Vascular cell* 4, 1. doi:10.1186/2045-824X-4-13

Nishishita, T. and Lin, P. C. (2004). Angiopoietin 1, PDGF-B, and TGF-β gene regulation in endothelial cell and smooth muscle cell interaction. *Journal of cellular biochemistry* 91, 584–593. doi:10.1002/jcb.10718

Niu, G., Wright, K. L., Huang, M., Song, L., Haura, E., Turkson, J., et al. (2002). Constitutive Stat3 activity up-regulates VEGF expression and tumor angiogenesis. *Oncogene* 21, 2000. doi:10.1038/sj.onc.1205260

Nowak, D. G., Amin, E. M., Rennel, E. S., Hoareau-Aveilla, C., Gammons, M., Damodoran, G., et al. (2010). Regulation of vascular endothelial growth factor (VEGF) splicing from pro-angiogenic to anti-angiogenic isoforms a novel therapeutic strategy for angiogenesis. *Journal of Biological Chemistry* 285, 5532–5540. doi:10.1074/jbc.M109.074930

Nowak, D. G., Woolard, J., Amin, E. M., Konopatskaya, O., Saleem, M. A., Churchill, A. J., et al. (2008). Expression of pro-and anti-angiogenic isoforms of VEGF is differentially regulated by splicing and growth factors. *Journal of cell science* 121, 3487–3495. doi:10.1242/jcs.016410

Oh, H., Takagi, H., Otani, A., Koyama, S., Kemmochi, S., Uemura, A., et al. (2002). Selective induction of neuropilin-1 by vascular endothelial growth factor (vegf): a mechanism contributing to vegf-induced angiogenesis. *Proceedings of the National Academy of Sciences* 99, 383–388. doi:10.1073/pnas.012074399

Ohne, Y., Takahara, T., and Maeda, T. (2009). Evaluation of mTOR function by a gain-of-function approach. *Cell Cycle* 8, 573–579. doi:10.4161/cc.8.4.7660

Oliveira, H., Catros, S., Boiziau, C., Siadous, R., Marti-Munoz, J., Bareille, R., et al. (2016). The proangiogenic potential of a novel calcium releasing biomaterial: Impact on cell recruitment. *Acta biomaterialia* 29, 435–445. doi:10.1016/j.actbio.2015.10.003

Otrock, Z. K., Mahfouz, R. A., Makarem, J. A., and Shamseddine, A. I. (2007). Understanding the biology of angiogenesis: review of the most important molecular mechanisms. *Blood Cells, Molecules, and Diseases* 39, 212–220. doi:10.1016/j.bcmd.2007.04.001

Pardali, E. and Ten Dijke, P. (2012). TGFbeta signaling and cardiovascular diseases. *Int J Biol Sci* 8, 195–213. doi:10.7150/ijbs.3805

Pasmant, E., Gilbert-Dussardier, B., Petit, A., de Laval, B., Luscan, A., Gruber, A., et al. (2015). SPRED1, a RAS MAPK pathway inhibitor that causes Legius syndrome, is a tumour suppressor downregulated in paediatric acute myeloblastic leukaemia. *Oncogene* 34, 631–638. doi:10.1074/jbc.M115.703710

Pate, K. T., Stringari, C., Sprowl-Tanio, S., Wang, K., TeSlaa, T., Hoverter, N. P., et al. (2014). Wnt signaling directs a metabolic program of glycolysis and angiogenesis in colon cancer. *The EMBO journal* 33, 1454–1473. doi:10.15252/embj.201488598

Pedrosa, A.-R., Trindade, A., Fernandes, A.-C., Carvalho, C., Gigante, J., Tavares, A. T., et al. (2015). Endothelial Jagged1 antagonizes Dll4 regulation of endothelial branching and promotes vascular maturation downstream of Dll4/notch1. *Arteriosclerosis, thrombosis, and vascular biology* , ATVBAHA–114doi:10.1161/ATVBAHA.114.304741

Peghaire, C., Bats, M. L., Sewduth, R., Jeanningros, S., Jaspard, B., Couffinhal, T., et al. (2016). Fzd7 (Frizzled-7) Expressed by Endothelial Cells Controls Blood Vessel Formation Through Wnt/β-Catenin Canonical Signaling. *Arteriosclerosis, Thrombosis, and Vascular Biology* , ATVBAHA–116doi:10.1161/ATVBAHA.116.307926

Peng, X., Ueda, H., Zhou, H., Stokol, T., Shen, T.-L., Alcaraz, A., et al. (2004). Overexpression of focal adhesion kinase in vascular endothelial cells promotes angiogenesis in transgenic mice. *Cardiovascular research* 64, 421–430. doi:10.1016/j.cardiores.2004.07.012

Peyton, K. J., Liu, X.-m., Yu, Y., Yates, B., and Durante, W. (2012). Activation of AMP-activated protein kinase inhibits the proliferation of human endothelial cells. *Journal of Pharmacology and Experimental Therapeutics* 342, 827–834. doi:10.1124/jpet.112.194712

Phng, L.-K., Potente, M., Leslie, J. D., Babbage, J., Nyqvist, D., Lobov, I., et al. (2009). Nrarp coordinates endothelial Notch and Wnt signaling to control vessel density in angiogenesis. *Developmental cell* 16, 70–82. doi:10.1016/j.devcel.2008.12.009

Phung, T. L., Ziv, K., Dabydeen, D., Eyiah-Mensah, G., Riveros, M., Perruzzi, C., et al. (2006). Pathological angiogenesis is induced by sustained Akt signaling and inhibited by rapamycin. *Cancer cell* 10, 159–170. doi:10.1016/j.ccr.2006.07.003

Presta, M., DellEra, P., Mitola, S., Moroni, E., Ronca, R., and Rusnati, M. (2005). Fibroblast growth factor/fibroblast growth factor receptor system in angiogenesis. *Cytokine & growth factor reviews* 16, 159–178. doi:10.1016/j.cytogfr.2005.01.004

Pugh, C. W. and Ratcliffe, P. J. (2003). Regulation of angiogenesis by hypoxia: role of the HIF system. *Nature medicine* 9, 677–684. doi:10.1038/nm0603-677

Pyriochou, A., Zhou, Z., Koika, V., Petrou, C., Cordopatis, P., Sessa, W. C., et al. (2007). The phosphodiesterase 5 inhibitor sildenafil stimulates angiogenesis through a protein kinase G/MAPK pathway. *Journal of cellular physiology* 211, 197–204. doi:10.1002/jcp.20929

Rad, E. and Tee, A. R. (2016). Neurofibromatosis type 1: Fundamental insights into cell signalling and cancer. In *Seminars in cell & developmental biology* (Elsevier), vol. 52, 39–46. doi:10.1016/j.semcdb.2016.02.007

Ranade, S. S., Qiu, Z., Woo, S.-H., Hur, S. S., Murthy, S. E., Cahalan, S. M., et al. (2014). Piezo1, a mechanically activated ion channel, is required for vascular development in mice. *Proceedings of the National Academy of Sciences* 111, 10347–10352. doi:10.1073/pnas.1409233111

Reis, M. and Liebner, S. (2013). Wnt signaling in the vasculature. *Experimental cell research* 319, 1317–1323. doi:10.1016/j.yexcr.2012.12.023

Rey, S. and Semenza, G. L. (2010). Hypoxia-inducible factor-1-dependent mechanisms of vascularization and vascular remodeling. *Cardiovascular research* , cvq045doi:10.1093/cvr/cvq045

Ribatti, D. (2008). The discovery of the placental growth factor and its role in angiogenesis: a historical review. *Angiogenesis* 11, 215–221. doi:10.1007/s10456-008-9114-4

Roukens, M. G., Alloul-Ramdhani, M., Baan, B., Kobayashi, K., Peterson-Maduro, J., van Dam, H., et al. (2010). Control of endothelial sprouting by a Tel-CtBP complex. *Nature cell biology* 12, 933–942. doi:10.1038/ncb2096

Ruan, G.-X. and Kazlauskas, A. (2012). Axl is essential for VEGF-A-dependent activation of PI3K/Akt. *The EMBO journal* 31, 1692–1703. doi:10.1038/emboj.2012.21

Saaristo, A., Veikkola, T., Enholm, B., Hytönen, M., Arola, J., Pajusola, K., et al. (2002). Adenoviral VEGF-C overexpression induces blood vessel enlargement, tortuosity, and leakiness but no sprouting angiogenesis in the skin or mucous membranes. *FASEB journal: official publication of the Federation of American Societies for Experimental Biology* 16, 1041. doi:10.1096/fj.01-1042com

Sakurai, Y., Ohgimoto, K., Kataoka, Y., Yoshida, N., and Shibuya, M. (2005). Essential role of Flk-1 (VEGF receptor 2) tyrosine residue 1173 in vasculogenesis in mice. *Proceedings of the National Academy of Sciences of the United States of America* 102, 1076–1081. doi:10.1073/pnas.0404984102

Serban, D., Leng, J., and Cheresh, D. (2008). H-ras regulates angiogenesis and vascular permeability by activation of distinct downstream effectors. *Circulation research* 102, 1350–1358. doi:10.1161/CIRCRESAHA.107.169664

Serra, H., Chivite, I., Angulo-Urarte, A., Soler, A., Sutherland, J. D., Arruabarrena-Aristorena, A., et al. (2015). PTEN mediates Notch-dependent stalk cell arrest in angiogenesis. *Nature communications* 6. doi:10.1038/ncomms8935

Sessa, R., Seano, G., di Blasio, L., Gagliardi, P. A., Isella, C., Medico, E., et al. (2012). The mir-126 regulates angiopoietin-1 signaling and vessel maturation by targeting p85β. *Biochimica et Biophysica Acta (BBA)-Molecular Cell Research* 1823, 1925–1935. doi:10.1016/j.bbamcr.2012.07.011

Sewduth, R. and Santoro, M. M. (2016). decoding angiogenesis: new facets controlling endothelial cell behavior. *Frontiers in Physiology* 7. doi:10.3389/fphys.2016.00306

Shawber, C. J., Funahashi, Y., Francisco, E., Vorontchikhina, M., Kitamura, Y., Stowell, S. A., et al. (2007). Notch alters VEGF responsiveness in human and murine endothelial cells by direct regulation of VEGFR-3 expression. *The Journal of clinical investigation* 117, 3369–3382. doi:10.1172/JCI24311

Shen, W.-G., Peng, W.-X., Dai, G., Xu, J.-F., Zhang, Y., and Li, C.-J. (2007). Calmodulin is essential for angiogenesis in response to hypoxic stress in endothelial cells. *Cell biology international* 31, 126–134. doi:10.1016/j.cellbi.2006.09.017

SHIGEMATSU, S., YAMAUCHI, K., NAKAJIMA, K., IIJIMA, S., AIZAWA, T., and HASHIZUME, K. (1999). IGF-1 regulates migration and angiogenesis of human endothelial cells. *Endocrine journal* 46, S59–S62. doi:10.1507/endocrj.46.Suppl_S59

Simons, M., Gordon, E., and Claesson-Welsh, L. (2016). Mechanisms and regulation of endothelial VEGF receptor signalling. *Nature Reviews Molecular Cell Biology* 17, 611–625. doi:10.1038/nrm.2016.87

Sivaraj, K. K. and Adams, R. H. (2016). Blood vessel formation and function in bone. *Development* 143, 2706–2715. doi:10.1242/dev.136861

Sivaraj, K. K., Li, R., Albarran-Juarez, J., Wang, S., Tischner, D., Grimm, M., et al. (2015). Endothelial Gαq/11 is required for VEGF-induced vascular permeability and angiogenesis. *Cardiovascular research* 108, 171–180. doi:10.1093/cvr/cvv216

Smadja, D. M., d'Audigier, C., Weiswald, L.-B., Badoual, C., Dangles-Marie, V., Mauge, L., et al. (2010). The Wnt antagonist Dickkopf-1 increases endothelial progenitor cell angiogenic potential. *Arteriosclerosis, thrombosis, and vascular biology* 30, 2544–2552. doi:10.1161/ATVBAHA.110.213751

Sonveaux, P., Copetti, T., De Saedeleer, C. J., Végran, F., Verrax, J., Kennedy, K. M., et al. (2012). Targeting the lactate transporter MCT1 in endothelial cells inhibits lactate-induced HIF-1 activation and tumor angiogenesis. *PloS one* 7, e33418. doi:10.1371/journal.pone.0033418

Spyridopoulos, I., Luedemann, C., Chen, D., Kearney, M., Chen, D., Murohara, T., et al. (2002). Divergence of angiogenic and vascular permeability signaling by VEGF. *Arteriosclerosis, thrombosis, and vascular biology* 22, 901–906. doi:10.1161/01.ATV.0000020006.89055.11

Sriram, K., Laughlin, J. G., Rangamani, P., and Tartakovsky, D. M. (2016). Shear-Induced Nitric Oxide Production by Endothelial Cells 111, 208–221. doi:10.1016/j.bpj.2016.05.034

Stahmann, N., Woods, A., Spengler, K., Heslegrave, A., Bauer, R., Krause, S., et al. (2010). Activation of AMP-activated protein kinase by vascular endothelial growth factor mediates endothelial angiogenesis independently of nitric-oxide synthase. *Journal of Biological Chemistry* 285, 10638–10652. doi:10.1074/jbc.M110.108688

Stefater III, J. A., Lewkowich, I., Rao, S., Mariggi, G., Carpenter, A. C., Burr, A. R., et al. (2011). Regulation of angiogenesis by a non-canonical Wnt-Flt1 pathway in myeloid cells. *Nature* 474, 511–515. doi:10.1038/nature10085

Suzuma, K., Takahara, N., Suzuma, I., Isshiki, K., Ueki, K., Leitges, M., et al. (2002). Characterization of protein kinase C β isoform's action on retinoblastoma protein phosphorylation, vascular endothelial growth factor-induced endothelial cell proliferation, and retinal neovascularization. *Proceedings of the National Academy of Sciences* 99, 721–726. doi:10.1073/pnas.022644499

Syeda, R., Florendo, M. N., Cox, C. D., Kefauver, J. M., Santos, J. S., Martinac, B., et al. (2016). Piezo1 channels are inherently mechanosensitive. *Cell reports* 17, 1739–1746. doi:10.1016/j.celrep.2016.10.033

Tammela, T., Zarkada, G., Nurmi, H., Jakobsson, L., Heinolainen, K., Tvorogov, D., et al. (2011). VEGFR-3 controls tip to stalk conversion at vessel fusion sites by reinforcing Notch signalling. *Nature cell biology* 13, 1202–1213. doi:10.1038/ncb2331

Tashiro, E., Tsuchiya, A., and Imoto, M. (2007). Functions of cyclin D1 as an oncogene and regulation of cyclin D1 expression. *Cancer science* 98, 629–635. doi:10.1111/j.1349-7006.2007.00449.x

Taylor, L. M. and Khachigian, L. M. (2000). Induction of platelet-derived growth factor B-chain expression by transforming growth factor-β involves transactivation by smads. *Journal of Biological Chemistry* 275, 16709–16716. doi:10.1074/jbc.275.22.16709

Teruyama, K., Abe, M., Nakano, T., Takahashi, S., Yamada, S., and Sato, Y. (2001). Neurophilin-1 is a downstream target of transcription factor Ets-1 in human umbilical vein endothelial cells. *FEBS letters* 504, 1–4. doi:10.1016/S0014-5793(01)02724-7

Tian, Y., Cohen, E. D., and Morrisey, E. E. (2010). The importance of Wnt signaling in cardiovascular development. *Pediatric cardiology* 31, 342–348. doi:10.1007/s00246-009-9606-z

Tsimafeyeu, I., Ludes-Meyers, J., Stepanova, E., Daeyaert, F., Khochenkov, D., Joose, J.-B., et al. (2016). Targeting FGFR2 with alofanib (RPT835) shows potent activity in tumour models. *European Journal of Cancer* 61, 20–28. doi:10.1016/j.ejca.2016.03.068

Uchida, C., Gee, E., Ispanovic, E., and Haas, T. L. (2008). JNK as a positive regulator of angiogenic potential in endothelial cells. *Cell biology international* 32, 769–776. doi:10.1016/j.cellbi.2008.03.005

Uebelhoer, M., Nätynki, M., Kangas, J., Mendola, A., Nguyen, H.-L., Soblet, J., et al. (2013). Venous malformation-causative TIE2 mutations mediate an akt-dependent decrease in pdgfb. *Human molecular genetics* , ddt198doi:10.1093/hmg/ddt198

Upton, P. D., Davies, R. J., Trembath, R. C., and Morrell, N. W. (2009). Bone morphogenetic protein (BMP) and activin type ii receptors balance BMP9 signals mediated by activin receptor-like kinase-1 in human pulmonary artery endothelial cells. *Journal of Biological Chemistry* 284, 15794–15804. doi:10.1074/jbc.M109.002881

Valter, M. M., Hügel, A., Huang, H. S., Cavenee, W. K., Wiestler, O. D., Pietsch, T., et al. (1999). Expression of the Ets-1 transcription factor in human astrocytomas is associated with Fms-like tyrosine

kinase-1 (Flt-1)/vascular endothelial growth factor receptor-1 synthesis and neoangiogenesis. *Cancer research* 59, 5608–5614

Vittet, D., Buchou, T., Schweitzer, A., Dejana, E., and Huber, P. (1997). Targeted null-mutation in the vascular endothelial–cadherin gene impairs the organization of vascular-like structures in embryoid bodies. *Proceedings of the National Academy of Sciences* 94, 6273–6278. doi:10.1152/physrev.00016.2015

Wang, S., Aurora, A. B., Johnson, B. A., Qi, X., McAnally, J., Hill, J. A., et al. (2008). The endothelial-specific microrna mir-126 governs vascular integrity and angiogenesis. *Developmental cell* 15, 261–271. doi:10.1016/j.devcel.2008.07.002

Wang, S., Lu, J., You, Q., Huang, H., Chen, Y., and Liu, K. (2016a). The mTOR/AP-1/VEGF signaling pathway regulates vascular endothelial cell growth. *Oncotarget* 7, 53269. doi:10.18632/oncotarget.10756

Wang, Y., Chang, H., Rattner, A., and Nathans, J. (2016b). Chapter Seven - Frizzled Receptors in Development and Disease. In *Essays on Developmental Biology, Part B*, ed. P. M. Wassarman (Academic Press), vol. 117 of *Current Topics in Developmental Biology*. 113 – 139. doi:10.1016/bs.ctdb.2015.11.028

Weiss, A. and Attisano, L. (2013). The TGFbeta superfamily signaling pathway. *Wiley Interdisciplinary Reviews: Developmental Biology* 2, 47–63. doi:10.1002/wdev.86

Werdich, X. Q. and Penn, J. S. (2006). Src, Fyn and Yes play differential roles in VEGF-mediated endothelial cell events. *Angiogenesis* 8, 315–326. doi:10.1007/s10456-005-9021-x

White, J. P., Cibelli, M., Urban, L., Nilius, B., McGeown, J. G., and Nagy, I. (2016). TRPV4: molecular conductor of a diverse orchestra. *Physiological reviews* 96, 911–973. doi:10.1152/physrev.00016.2015

Wilhelm, K., Happel, K., Eelen, G., Schoors, S., Oellerich, M. F., Lim, R., et al. (2016). FOXO1 couples metabolic activity and growth state in the vascular endothelium. *Nature* 529, 216–220. doi:10.1038/nature16498

Wimmer, R., Cseh, B., Maier, B., Scherrer, K., and Baccarini, M. (2012). Angiogenic sprouting requires the fine tuning of endothelial cell cohesion by the Raf-1/Rok-α complex. *Developmental cell* 22, 158–171. doi:10.1016/j.devcel.2011.11.012

Wu, X. Y., Xu, H., Wu, Z. F., Chen, C., Liu, J. Y., Wu, G. N., et al. (2015). Formononetin, a novel FGFR2 inhibitor, potently inhibits angiogenesis and tumor growth in preclinical models. *Oncotarget* 6, 44563. doi:10.18632/oncotarget.6310

Wythe, J. D., Dang, L. T., Devine, W. P., Boudreau, E., Artap, S. T., He, D., et al. (2013). ETS factors regulate Vegf-dependent arterial specification. *Developmental cell* 26, 45–58. doi:10.1016/j.devcel.2013.06.007

Xia, P., Chen, H.-y., Chen, S.-f., Wang, L., Strappe, P. M., Yang, H.-l., et al. (2016). The stimulatory effects of eNOS/F92A-Cav1 on NO production and angiogenesis in BMSCs. *Biomedicine & Pharmacotherapy* 77, 7–13. doi:10.1016/j.biopha.2015.11.001

Xiong, S., Salazar, G., Patrushev, N., and Alexander, R. W. (2011). Foxo1 mediates an autofeedback loop regulating SIRT1 expression. *Journal of Biological Chemistry* 286, 5289–5299. doi:10.1074/jbc.M110.163667

Yamashita, M., Fatyol, K., Jin, C., Wang, X., Liu, Z., and Zhang, Y. E. (2008). TRAF6 mediates Smad-independent activation of JNK and p38 by TGF-β. *Molecular cell* 31, 918–924. doi:10.1016/j.molcel.2008.09.002

Yang, C.-S., Hung, K.-C., Huang, Y.-M., and Hsu, W.-M. (2013). Intravitreal bevacizumab (Avastin) and panretinal photocoagulation in the treatment of high-risk proliferative diabetic retinopathy. *Journal of Ocular Pharmacology and Therapeutics* 29, 550–555. doi:10.1089/jop.2012.0202

Yang, H., Liu, C., Zhou, R.-M., Yao, J., Li, X.-M., Shen, Y., et al. (2016a). Piezo2 protein: A novel regulator of tumor angiogenesis and hyperpermeability. *Oncotarget* 7, 44630. doi:10.18632/oncotarget.10134

Yang, L., Xiao, M., Li, X., Tang, Y., and Wang, Y.-L. (2016b). Arginine ADP-ribosyltransferase 1 promotes angiogenesis in colorectal cancer via the PI3K/Akt pathway. *International journal of molecular medicine* 37, 734–742. doi:10.3892/ijmm.2016.2473

Ye, F.-C., Blackbourn, D. J., Mengel, M., Xie, J.-P., Qian, L.-W., Greene, W., et al. (2007). Kaposi's sarcoma-associated herpesvirus promotes angiogenesis by inducing angiopoietin-2 expression via AP-1 and Ets1. *Journal of virology* 81, 3980–3991. doi:10.1128/JVI.02089-06

Zarate, Y. A., Lichty, A. W., Champion, K. J., Clarkson, L. K., Holden, K. R., and Matheus, M. G. (2014). Unique cerebrovascular anomalies in Noonan syndrome with RAF1 mutation. *Journal of child neurology* 29, NP13–NP17. doi:10.1177/0883073813492384

Zavadil, J., Cermak, L., Soto-Nieves, N., and Böttinger, E. P. (2004). Integration of TGF-β/Smad and Jagged1/Notch signalling in epithelial-to-mesenchymal transition. *The EMBO journal* 23, 1155–1165. doi:10.1038/sj.emboj.7600069

Zerlin, M., Julius, M. A., and Kitajewski, J. (2008). Wnt/Frizzled signaling in angiogenesis. *Angiogenesis* 11, 63–69. doi:10.1007/s10456-008-9095-3

Zhang, Y., Call, M. K., Yeh, L.-K., Liu, H., Kochel, T., Wang, I.-J., et al. (2010). Aberrant expression of a β-catenin gain-of-function mutant induces hyperplastic transformation in the mouse cornea. *J Cell Sci* 123, 1285–1294. doi:doi:10.1242/jcs.063321

Zmajkovicova, K., Jesenberger, V., Catalanotti, F., Baumgartner, C., Reyes, G., and Baccarini, M. (2013). MEK1 is required for PTEN membrane recruitment, AKT regulation, and the maintenance of peripheral tolerance. *Molecular cell* 50, 43–55. doi:10.1016/j.molcel.2013.01.037

Supplementary Table 2. Mutations that affect response to mechanical forces (Kutys and Chen, 2016)

Gene	Reduced function	Augmented function
Shear Stress	The difference in blood flow between different branches of a blood vessel networkmay cause blood vessel pruning (Betz et al., 2016).	Applied shear stress induces cellular and cytoskeletal alignment in the direction of flow and the strengthening of cell-cell adherens junction complexes. Intramolecular tension increases across PECAM1, but decreases across VE-cadherin and cell-cell adhesions. Triggers local angiogenic sprouting.
miR-126	In mice, vascular leakage, hemorrhaging, and embryonic lethality, elevated rate of proliferation, reduced migration in response to VEGF and FGF, defective VEGF–induced actin cytoskeleton rearrangement (Wang et al., 2008; Fish et al., 2008).	miR-126 over expression does not affect the expression of EC specific genes. However, it causes increased migration in response to VEGF stimulation (Fish et al., 2008).
SPRED1	No effect on VEGF-induced endothelial cell migration. However, rescued the migration defect in cells with decreased miR-126 expression (Fish et al., 2008).	In zebrafish, it disrupts vascular integrity, similar to miR-126 knockdown (Fish et al., 2008)
PI3KR2	Rescues AKT phosphorylation defects caused by miR126(rf) (Sessa et al., 2012).	Over-expression inhibits AKT phosphorylation via TIE2 (Sessa et al., 2012)
NF1	Neurofibromatosis type one, increased RAS activity, immature microvascular blood vessels similar to sprouting angiogenesis in tumors (Rad and Tee, 2016).	NA
KLF2	In mice, embryonic lethal due to deficient blood vessel stabilization (Dekker et al., 2006).	Major reorganization of the cytoskeleton characterized by the formation of typical stress fibers (Dekker et al., 2006).
PIEZO1	In mice, death at midgestation with defects in vascular remodeling (Ranade et al., 2014).	Large mechanically activated currents (Syeda et al., 2016).
TRPV4	Shear-induced increases in endothelial Ca^{2+} are inhibited by ruthenium red, which blocks TRPV4, and by knockdown of TRPV4 using siRNA (White et al., 2016).	Activation of TRPV4 with 4-PDD causes EC-mediated artery dilation (White et al., 2016).
Integrin $\alpha_v\beta_3$	Deletion in some mice causes hemorrhage induced intrauterine death. Phosphorylation of Tyr747 and Tyr459 necesary for angiogenesis. Inhibitory antibodies or low-molecular weight antagonists inhibit EC adhesion (Hodivala-Dilke, 2008).	Mutated Tyr747 and Tyr459 inhibits angiogenesis (Hodivala-Dilke, 2008)
GqG11	In ECs, reduced VEGFA-induced VEGFR2 autophosphorylation, Ca mobilization, and phosphorylation of SRC and VE-cadherin, impairing barrier opening, tube formation, and proliferation. (Sivaraj et al., 2015).	Agonists facilitated VEGFA-mediated VEGFR2 autophosphorylation (Sivaraj et al., 2015)
Actin	In mice, *Actc1* perinatal lethal, *Acta2* impaired vascular contractility (Ampe and Van Troys, 2017).	NA
VE–cadherin	ECs failed to organize a vessel-like pattern (Vittet et al., 1997). Reduced expression prevents the formation of a stable vasculature. Defective anastomosis in zebrafish sprouting vessels (Giannotta et al., 2013).	Stabilization at adherens junctions limits VEGFR signaling, and aberrant angiogenesis (Giannotta et al., 2013).
PECAM–1	Anti-PECAM-1 antibodies inhibit the ability of ECs to form tube-like structures, and when injected into mice, cause fluid leak into the hepatic and renal vasculature (Lertkiatmongkol et al., 2016).	Endothelial cell monolayers exhibit augmented barrier function (Lertkiatmongkol et al., 2016).
SHP2	Prevents HUVEC capillary-like structure formation in vitro, inhibits new vessel growth ex vivo in mice and in vivo in the chicken chorioallantoic membrane. Increased EC apoptosis (Mannell et al., 2008)	NA

Supplementary Table 3. Mutations that affect the PI3K/AKT signaling pathway

Gene	Reduced function	Augmented function
FAK	Lethal in mice (E8.5) with extensive defects in vasculogenesis and angiogenesis (Peng et al., 2004).	In mice, the number of vessels in the granulation tissue of a healing wound increased (Peng et al., 2004)
SRC	Inhibits VEGF induced angiogenesis in vivo. Defective EC migration, polarization, and cytoskeleton remodeling, inhibits VE-cadherin phosphorylation and turnover (Kilarski et al., 2003; Gordon et al., 2016).	More pronounced defects in EC migration, polarization, and cytoskeleton remodeling(Kilarski et al., 2003).
PI3K	Defective EC migration, angiogenesis, and vascular development (Karar and Maity, 2011). Increases capillary density and reduces pericyte coverage (Kerr et al., 2016).	Induces abnormal angiogenesis and increased VEGFA expression (Phung et al., 2006; Karar and Maity, 2011)
AKT	Loss of both *Akt1 and Akt2* is lethal in mice (Dummler et al., 2006). Adult EC specific loss of AKT causes VSMC, arteriole and artery loss in mice hearts despite high capillary density. In mice retina, EC basement membrane deterioration and vascular regression (Kerr et al., 2016).	Restores VSMC coverage (Kerr et al., 2016).
TSC	In humans causes tuberous sclerosis, Tie2-Cre/Tsc1$^{-/-}$ mice embryos died (E14.5), defective angiogenesis, induces EC apoptosis, decreases EC proliferation (Ma et al., 2014).	NA
AXL	Impaired angiogenesis and tube formation in humans and mice, inhibits VEGFAdependent activation of AKT (Ruan and Kazlauskas, 2012).	Associated with cancer(Ruan and Kazlauskas, 2012).
PIP3	see PI3K(lf)	see PTEN(lf)
RHEB2	Murine embryonic lethality (E12), circulatory failure (Goorden et al., 2011).	NA.
FYN	In human retinal ECs, increased cell migration and impaired vascular tube formation in response to VEGF (Werdich and Penn, 2006).	NA
TSAd	Impaired angiogenic sprout growth in mice and embrioid bodies, does not affect tip cell specification (Gordon et al., 2016).	NA
PTEN	mice died before embryonic day 11.5 (E11.5) due to bleeding and cardiac failure caused by impaired recruitment of pericytes and vascular smooth muscle cells (Hamada et al., 2005).	Overexpression inhibits tumor angiogenesis in vivo (Hamada et al., 2005)

Supplementary Table 4. Mutations that affect the TGF signaling pathway

Gene	Reduced function	Augmented function
BMP9	No significant blood vessel phenotype (Jin et al., 2014).	NA
BMP10	Lethal in mice (E9.5E10.5), abnormal cardiac development(Jin et al., 2014).	NA
TGFβ1	In humans it causes the Camurati-Engelmann disease, cardiomyopathy and hypertension (Akhurst, 2004). In mice, lethal (E9.5E11.5) with defective vasculogenesis, those that survive present retinal haemorrhage and abnormal vasculature.	Induces anti-angiogenic splicing of VEGFA in human retinal ECs (Nowak et al., 2008).
ACVR2A	Reduced BMP9-mediated SMAD2 phosphorylation (Upton et al., 2009), together with BMPRII(lf), reduces SMAD1/5 phosphorylation.	NA
BMPRII	KO: Lethal in mice(E8.5). Heterozygous KO: Pulmonary arterial hypertension (PAH), reduced BMP9-mediated SMAD1 phosphorylation (Upton et al., 2009; Pardali and Ten Dijke, 2012).	NA
TGFβRII	In mice, lethal(E10.5) with defects in vasculogenesis, cerebral and retinal haemorrhage (Jin et al., 2014).	NA
ALK1	In mice, lethal(E11.5) with severe AVMs, EC specific KO; lethal pulmonary haemorrhage, AVM and hypersprouting in retinal vasculature (Jin et al., 2014). Heterozygous mutations cause Hereditary Hemorrhagic Telangiectasia type 2 (HHT2), and increased migratory and invasive response to FGF in ECs (Choi et al., 2013).	NA
ENG	In mice, lethal(E11.5), EC specific loss of function in neonate mice causes retinal AVMs, in adults it causes brain AVMs only in combination with VEGF (Jin et al., 2014). In humans, it causes HHT type 1 (Choi et al., 2013).	NA
ALK5	In mice, lethal(E10E11), defective yolk sac vascular development (Jin et al., 2014). LoeysDietz syndrome (LDS) patients present arterial aneurysms or dissections, and tortuosity (Pardali and Ten Dijke, 2012).	NA
BG	In mice, lethal(E18.5) (Jin et al., 2014).	
SMAD4	In mice, lethal(E7.5). EC specific knock out: lethal(E10.5) with reduced vascular sprouting, and ruptured blood vessels (Jin et al., 2014). In humans: Juvenile polyposis (JP) and HHT (Pardali and Ten Dijke, 2012)	NA
SMAD6	In mice: heart abnormalities, aortic ossification and elevated blood pressure (Pardali and Ten Dijke, 2012).	NA.
SMAD1	In mice: lethal(E9.5). EC specific knock out develops PAH.(Jin et al., 2014).	NA
SMAD2	In mice, lethal(E7.5E8.5)(Jin et al., 2014).	NA
PDGFB	In humans and mice, EC loss of function causes brain calcification due to blood-brain barrier defects. Pericytes are not recruited to developing blood microvessels in mice, and causing vascular dysfunction and perinatal death (Keller et al., 2013).	PDGFBR(gf) Leukemias, gastrointestinal sarcomas (Keller et al., 2013).
TRAF6	In mink lung ECs, TGF--induced activation of p38 is inhibited(Yamashita et al., 2008). Abnormal vertebral artery topology and subcutaneous edema in mice (https:/dmdd.org.uk)	NA
TAK1	In mice, defects in developmental angiogenesis (Jadrich et al., 2006).	In mice, cardiac hypertrophy (Jadrich et al., 2006).

Supplementary Table 5. Mutations that affect the Calcium, and NO signaling pathways

Gene	Reduced function	Augmented function
sGC	Increased risk of myocardial infraction in humans. Mice displayed accelerated thrombus formation in the microcirculation after local trauma (Erdmann et al., 2013).	NA
cGMP	see sGC.	Sildenafil mediated inhibition of cGMP-specific phosphodiesterase 5 (PDE5) promotes EC proliferation, migration, and organization into tube-like structures (Pyriochou et al., 2007).
eNOS	In humans, reduced NO production-mediated coronary artery spasm, associated with Prinzmetal's variant angina (Glueck et al., 2010). Mice showed significantly reduced angiogenesis, and impaired response to VEGFA (Fukumura et al., 2001).	In vitro capillary formation was increased in rat mesenchymal stem cells (BMSCs) (Xia et al., 2016)
NO	In mice, reduced angiogenesis and vascular permeability induced by VEGFA(Fukumura et al., 2001)	In vitro capillary formation was increased in rat mesenchymal stem cells (BMSCs) (Xia et al., 2016)
Calcium	Reduction of either extracellular or intracellular free Ca^{2+} in human umbilical vein endothelial cells (HUVECs), inhibits the proliferative response to VEGFA (Faehling et al., 2002)	In mice, calcium ions released by the degradation of calcium phosphate ormoglasses (CaP) promote angiogenesis (Oliveira et al., 2016)
Calmodulin	In HUVECs, suppressed endothelial cell migration, adhesion to collagen I substrate, invasion and formation of tube-like structures (Shen et al., 2007).	NA
Calcineurin	Lethal in mice(E11.5), defective vascular remodeling; excessive fusion into irregular dilated vessels (Graef et al., 2001).	NA
NFAT	NFAT c3/c4 null mice die (E11.5) with severely disorganized blood vessels and mural cell recruitment failure (Graef et al., 2001).	NA

Supplementary Table 6. Mutations that affect the RAS and PLCγ signaling pathways

Gene	Reduced function	Augmented function
ETS1	In mice, inhibits EC migration and cell proliferation, reduces *FGF-2*-mediated angiogenesis and inhibits retinal angiogenesis (Craig and Sumanas, 2016).	In mice, induces expression of *VEGFR2*. In HUVEC cells induces apoptosis. In mice, causes increased levels of MMP-1, MMP-3 and MMP-9 in ECs (Craig and Sumanas, 2016).
HRAS	In mice, increased vasodilatation, lower blood pressure and increased sGC and cGMP concentration in the aorta (Chamorro-Jorganes et al., 2010)	In HUVECs induces vascular tube formation in vitro, and induces angiogenesis in chic chorioallantoic membranes. In mice, Ras(G12$\rightarrow$V12) induces angiogenesis and vascular permeability, Ras(G12$\rightarrow$V12, T40$\rightarrow$C40) induces vascular permeability only, and Ras(G12$\rightarrow$V12, Y35$\rightarrow$S35) angiogenesis only (Serban et al., 2008)
RAF1	Inhibits sprouting angiogenesis and causes defects in cell adhesion in mice ECs (Wimmer et al., 2012). Uninhibited HUVEC apoptosis (Alavi et al., 2003)	In humans, Noonan syndrome including cerebrovascular anomalies (Zarate et al., 2014)
MEK	Mice died(E10.5) with a reduced number of ECs in the labyrinthine region (Giroux et al., 1999), and stabilizes AKT activation (Zmajkovicova et al., 2013).	NA.
ERK	In mice, loss of *Erk2* is lethal(E10.5), if activated later, may cause cardiovascular malformations (Frémin et al., 2015).	Atopic expression of *Erk1* can rescue *Erk2*$^{-/-}$ mutants (Frémin et al., 2015).
PLCγ	In mice, lethal(E9.09.5) (Chung et al., 2010), eliminating the ability of VEGFR2 to phosphorylate PLCγ prevents the formation of organized blood vessels (Sakurai et al., 2005). Loss of *D. rerio* segmental arteries and mutant embryos do not initiate blood circulation (Covassin et al., 2009)	(Chung et al., 2010)
IP3	see PLCγ	
DAG	see PLCγ	NA
PKCB2	Reduced VEGF-mediated EC proliferation and retinal vascular permeability, and a significant decrease in retinal neovascularization in mice (Suzuma et al., 2002). In HUVECs induced vascular permeability and abrogated VEGF-induced EC proliferation (Spyridopoulos et al., 2002).	Augmented angiogenic response to ischemia in mice (Suzuma et al., 2002).

Supplementary Table 7. Mutations that affect the response to Oxygen and energy availability

Gene	Reduced function	Augmented function
AMPATP	(Craig and Sumanas, 2016).	(Craig and Sumanas, 2016).
AMPK	(AMPK$\alpha 1^{-/-}$) Sprouting angiogenesis is inhibited in mice and HUVECs (Stahmann et al., 2010)	Inhibits HUVEC proliferation and DNA synthesis, inhibits EC migration, and induces vascular tube formation (Peyton et al., 2012)
mTOR	Rapmycin inhibits *mTOR* and angiogenesis (Karar and Maity, 2011)	Causes cell size to increase and inhibits autophagy (Ohne et al., 2009)
HIF-1	In mice, decreases SIRT1 levels (Chen et al., 2011). Deletion of *HIF-1*α in ECs inhibits tumor vascularization, impairs cell migration through endothelial layers (LaGory and Giaccia, 2016).	In mice, increases SIRT1 levels (Chen et al., 2011).
FOXO1	Mice die (E11) with vascular anormalities (Furuyama et al., 2004). Endothelial-restricted deletion in mice leads to increased EC proliferation, causing hyperplasia and vessel enlargement (Wilhelm et al., 2016).	In mice, it inhibits vascular growth and causes vessel thinning and hypobranching (Wilhelm et al., 2016).
ART1	Decreases *AKT, VEGFA* and FGF-2 activity in HUVECs (Yang et al., 2016b).	Increases *AKT, VEGFA* and FGF-2 activity in HUVECs (Yang et al., 2016b).
NAD	CypD silencing causes a reduction in the NAD + /NADH ratio that leads to increased angiogenesis and neovascularization (Marcu et al., 2015)	NA
Oxygen	Induces the differentiation of endothelial progenitor cells (EPCs) into ECs, upregulates angiogenesis (Fraisl et al., 2009)	Inhibits angioblast differentiation into ECs, induces EC disfunction and apoptosis (Fraisl et al., 2009).
Lactate	Inhibits HIF-1-dependent angiogenesis (Sonveaux et al., 2012).	Induces angiogenesis, induces the expression of *VEGFA* and enhances its function (Kumar et al., 2014)
PHDs	Increases HIF-1α and HIF-2α activity *appelhoff2004differential*. In mice, it enhances post-stroke recovery and angiogenesis (Li et al., 2016).	
HIF2a	Upregulates tumor angiogenesis, disorganized vasculature, eases cell migration through endothelial layers (LaGory and Giaccia, 2016).	Down-regulates cell growth and proliferation. Similar to hypoxia; increases basement membrane invasion and vascular tube formation (Manalo et al., 2005)
LSD1	In mice, it decreases the concentration of HIF-1 (Kim et al., 2016).	In mice, activates angiogenesis, and increases the concentration of HIF-1 in the nucleus (Kim et al., 2016).
SIRT1	In mice, increased genomic instability and tumorigenesis (Han et al., 2013), and mediates vascular aging (Kong et al., 2016).	In mice, negatively regulates tumorigenesis (Han et al., 2013), reduces vascular damage, including vessel tension, blood pressure, and EC mitochondrial function (Kong et al., 2016).

Supplementary Table 8. Mutations that affect the noncanonical *Wnt* signaling pathway

Gene	Reduced function	Augmented function
WNT5a	(Masckauchán et al., 2006). In HUVECs, reduces proliferation, survival, migration, vascular network formation	Increases HUVEC proliferation, survival, migration, vascular network formation, and induces phosphorylation of DVL-2, DVL-3, and ERK1/2 (Masckauchán et al., 2006).
RORα	In mice, neonatal lethality, cardiac defects and transposition of the great arteries (Minami et al., 2010).	NA
JNK	Reduced sprout growth, EC proliferation, migration, and proteolysis of the capillary basement membrane (Uchida et al., 2008)	NA
AP1	In HUVECS, inhibited angiogenesis in an inflammatory microenvironment (Huang et al., 2017). Decreases rat aortic EC proliferation (Wang et al., 2016a).	Increases rat aortic EC proliferation (Wang et al., 2016a).
FZD4_5	Disruption of the murine *Fzd-5* is lethal(E11.5) and loss of *Fzd-4* function causes cerebellar, auditory, and esophageal dysfunction (Zerlin et al., 2008). In humans, *FZD4* loss of function causes familial exudative vitreoretinopathy, characterized by defects in retinal angiogenesis (Gilmour, 2015). In human microvascular ECs, FZD5 siencing prevents the angiogenic effect of WNT5 (Arderiu et al., 2014)	NA
WNT11	Defective outflow tract development, and cardiac myocyte differentiation (Tian et al., 2010). Increased angiogenesis in the mouse retina (Stefater III et al., 2011).	NA
FZD6	Marked reduction in renal vasculature in mice (Linder et al., 2015)	NA
RAC1	Strongly inhibits angiogenesis, causes blood vessel leakage (Hoang et al., 2011)	Enhances VEGFA-induced angiogenesis, enhances lumen formation, and improved vascular stability.(Hoang et al., 2011)
DAAM1	Inhibits the proliferation of HUVEC and other cells (Ju et al., 2010).	Inhibits EC specific proliferation, migration, and angiogenesis by stabilazing microtubules in HUVECs and zebrafish (Ju et al., 2010).
CDC42	Negatively regulates vacuole formation and lumen formation in HUVECS (Bayless and Davis, 2002)	Increases HUVEC proliferation and vascular tube formation (Ma et al., 2013)
RHOA	Inhibits VEGFA-induced angiogenesis n human ECs implanted into mice; reduced the number of actin stress fibers, prevented EC contraction and reorganization into precapillary cords (Hoang et al., 2004)	ENhances VEGFA-induced angiogenesis; promotes the formation of actin stress fibers, EC contractility, and vascular tube formation(Hoang et al., 2004)

Supplementary Table 9. Mutations that affect the canonical *Wnt* signaling pathway

Gene	Reduced function	Augmented function
WNT7a	Mice die(E11.5) with defective central nervous system vasculature; reduced capillary density, vascular malformations (Daneman et al., 2009).	Induces the migration of mouse brain ECs (Daneman et al., 2009)
sFRP1	*sFrp1* knockout mice with are viable and fertile, however *sFrp1* and *sFrp2*double knockout mice is embryonic lethal (Cruciat and Niehrs, 2013).	Inhibits the formation and migration of human ECs and mouse endothelial progenitor cells, and induces apoptosis of the latter (Hu et al., 2009)
FZD4_7	In mice, loss of *Fzd-4* function causes cerebellar, auditory, and esophageal dysfunction (Zerlin et al., 2008). In mice EC specific deletion of *Fzd7* increased tip EC differentiation and the number of filipodia. Additionally, reduced stalk cell proliferation (Peghaire et al., 2016)	NA
LRP5_6	*LRP5* mutations cause familial exudative vitreoretinopathy in humans (Fei et al., 2014), in humans, *LRP6* loss of function suppresses endothelial cell proliferation and migration and is associated with coronary artery disease (Guo et al., 2016).	NA
DKK1	Decreased the vascular tube formation potential of human culture forming ECs (Smadja et al., 2010).	Increased vascular tube formation and proliferation rate of human culture forming ECs (Smadja et al., 2010).
DSH	In mice EC specific suppression of *Dvl1* and *Dvl3* increased the number of tip cells (Peghaire et al., 2016).	NA
GSK3β	Enhanced angiogenesis, EC migration and promoted EC survival in HUVECs (Kim et al., 2002)	Diminished migration in response to response to VEGFA, induced EC apoptosis, and inhibited apoptosis in HUVECs (Kim et al., 2002).
Axin2	Increases the β-catenin concentration, increases HUVEC proliferation, migration, and tube formation (Yang et al., 2016a)	NA
BTrCP	Increases the concentration of *βcatenin* (Liu et al., 2004).	Reduces the concentration of *βcatenin* (Liu et al., 2004).
βcatenin	In mice, endothelial deletion of *Ctnnb1* decreases retinal blood vessel density and causes ectopic blood vessel regression (Phng et al., 2009), defective angiogenesis, and increased vascular fragility (Cattelino et al., 2003).	In mouse cornea, induces EC proliferation and angiogenesis (Zhang et al., 2010).
GROUCHO	NA	Repress TCF-mediated transcription in mice and humans (Brantjes et al., 2001)
LEF-1	Reduced blood vessel density in mice with implanted colon cancer cells (Pate et al., 2014).	NA
Cyclin D1	Inhibits EC migration, induces apoptosis, and G1 cell cycle arrest in HUVECs (Hanai et al., 2002).	Shortens the G1 phase of the cell cycle in fibroblast cells (Tashiro et al., 2007).

Supplementary Table 10. Mutations that affect the *FGF* and *NOTCH* signaling pathways

Gene	Reduced function	Augmented function
FGF2	*fgfr1-2* null mutations are lethal in mice. Latter expression of dominant negative FGFR1 causes a significant impairment of blood vessel development and maintenance in mice. In chicks, anti-FGF2 antibodies prevent neovascularization (Presta et al., 2005).	FGF1-4 activate mouse EC proliferation, migration, and angiogenesis. Additionally, extracellular matrix degradation, remodeling and blood vessel stabilization (Presta et al., 2005)
FGFR2	The FGFR2 inhibitors formononetin (Wu et al., 2015) and alofanib (Tsimafeyeu et al., 2016) inhibit FGFR2-mediated angiogenesis in HUVECs.	NA
FRS2α	In mice ECspecific deletion causes the shape ECs to change. Further, the ECs express smooth muscle cell markers and secrete more collagen. Reduces HUVEC proliferation and blood vessel formation (Chen et al., 2014).	Promotes prostate cancer tumor angiogenesis (Liu et al., 2016)
JAG	*Jag1*-null mouse mutants die (E11.5) with vascular defects in the head. Human *JAG1* mutations cause Alagille syndrome. Decreases blood vessel density, maturation, and perfusion (Pedrosa et al., 2015).	Increases blood vessel density, maturation, and perfusion (Pedrosa et al., 2015). Fewer tip cells and vessel branches (Hellström et al., 2007).
DLL4	In mice knockout leads to death during early embryogenesis from vascular defects. Haploinsufficiency causes defects in vascular remodeling, arteriovenous malformations, and incomplete artery formation (Blanco and Gerhardt, 2013). Increases vascular density and reduces perivascular cell coverage in mice (Pedrosa et al., 2015).	Increases the perivascular cell coverage in mice (Pedrosa et al., 2015).
ADAM10	Increases vascular branching and density in the retina of mice (Caolo et al., 2015).	()
γSecretase	Increases vascular branching and density in the retina of mice (Caolo et al., 2015).	()
NOTCH4	Mice develop normally, are viable and fertile (Krebs et al., 2000).	Mice die(E10) (Blanco and Gerhardt, 2013). Promotes blood vessel maturation without affecting angiogenic growth (Pedrosa et al., 2015).
NOTCH1	Endothelial specific loss is lethal(E10.5) in mice with defective angiogenesis (Limbourg et al., 2005). ECs preferentially assume tip-cell characteristics (Hellström et al., 2007).	Reduces the number of tip cells (Hellström et al., 2007).
CSL	Mouse EC-specific deletion of CSL is lethal, with vascular defects and causes excessive sprouting and tip cell behavior in zebrafis(Blanco and Gerhardt, 2013).	NA
NRARP	Causes vessel regression. Causes delayed vascularization, and decreases blood vessel density in mice retina (Phng et al., 2009).	Inhibits the activity of Notch in*Xenopus* embryos (Lamar et al., 2001).
HEY1	*Hey1* knockout mice, develop normally. The combined loss of *Hey1* and *Hey2*, causes embryonic death(E9.5) lack of vascular remodeling and hemorrhageand fail to express arterial EC markers. (Fischer et al., 2004).	In human capillary ECs, blocked angiogenesis and vascular tube formation (Li and Harris, 2005).

Supplementary Table 11. Mutations that affect the *VEGF* signaling pathway

Gene	Reduced function	Augmented function
VEGFR2	Mice die(E8.5-9.5) without blood vessel organization (Sakurai et al., 2005)	Activating mutations cause vascular tumours (Carmeliet and Jain, 2011).
VEGFR1	Mice exhibit abnormal vascular channel formation, augmented EC proliferation, and die(E8.5) (Fong et al., 1995).	NA
PlGF	Mice exhibit normal development, reproduction, and postnatal life except reduced vascular dencity in adipose tissue. Significantly impaired recruitment of macrophages, angiogenesis and arteriogenesis in ischemia and tumors in mice (Ribatti, 2008).	Stimulates angiogenesis, arteriogenesis and blood vessel stabilization (Luttun et al., 2002).
VEGFR1s	Mice with only the cell-membrane bound isoform exhibit reduced sprouting and branch formation (Kappas et al., 2008).	In mice, it rescues both the EC proliferation and the blood vessel phenotypes caused by loss of VEGFR1 function(Kappas et al., 2008).
VEGFB	Inhibits coronary artery development in mouse embryos (Fischer et al., 2008)	In mice, cardiomyocyte hypertrophy, revascularization of ischaemic myocardium (Fischer et al., 2008)
IGF	Inhibits retinal angiogenesis (Delafontaine et al., 2004).	Induces the migration of HUVECs. In hypoglycemic conditions it induces vascular tube formation (SHIGEMATSU et al., 1999). Promotes rat aortic angiogenesis, promotes retinal angiogenesis, and inhibits EC apoptosis (Delafontaine et al., 2004). Promotes the expression of active VEGFA isoforms (Nowak et al., 2008).
PKC-α	Suppresses platelet VEGF secretion. Promotes PDGF-induced angiogenesis in HUVECs (Moriya and Ferrara, 2015).	Decreased proliferation and viability of HUVECs (Moriya and Ferrara, 2015).
ASF/SF2	Reduced retinal angiogenesis and neovascularization in mice (Nowak et al., 2010).	Promotes the expression of active VEGFA isoforms (Nowak et al., 2008).
VEGFAxxxP	Intravitreal bevacizumab (Avastin; An antibody that binds and inhibits VEGFA) inhibits retinal angiogenesis (Yang et al., 2013).	Induces angiogenesis in the cornea of mice, the new blood vessels are thin and highly permeable (Cao et al., 2004).
VEGFA	Mice and rats die(homozygous E8-9, hetherozygous E11-12) with impaired angiogenesis (Ferrara et al., 1996). Inhibits *VEGFR2, TIE2* and *VEcadherin* expression and *VEGFR2* function in HUVECs (Guangqi et al., 2012). EC specific deletion is also lethal in mice (Guangqi et al., 2012).	Mice die(E12.5-E14) with abnormal heart development (Guangqi et al., 2012).
p38-MAPK	Lethal in mice with defective placental and embryonic angiogenesis (Mudgett et al., 2000).	NA
CLK1	Inhibits TGFβ1-mediated anti-angiogenic splicing of VEGFA in human retinal ECs (Nowak et al., 2008).	?
CLK4	Inhibits TGFβ1-mediated anti-angiogenic splicing of VEGFA in human retinal ECs (Nowak et al., 2008).	
SRp55	The SRp55 2994 polymorphism is associated with proliferative diabetic retinopathy (Carter et al., 2011)	Augments anti-angiogenic splicing of VEGFA in human retinal ECs (Nowak et al., 2008)

Supplementary Table 12. Mutations that affect *VEGF* signaling pathway continued

Gene	Reduced function	Augmented function
VEGFAxxxd	NA	Inhibits VEGFA-mediated EC proliferation, migration, EC monolayer permeability and angiogenesis in humans and mice (Harper and Bates, 2008).
VEGFC	Mice die(E16.5) due to lymphatic vessel formation failure, and exhibit decreased vascular branching due to defective angiogenesis (Tammela et al., 2011).	Stimulates blood vessel enlargement, tortuosity, leakiness, and angiogenesis (Saaristo et al., 2002)
VEGFD	Mice are viable and normal (Tammela et al., 2011). Causes facial lymphangiogenesis defects in zebrafish (Bower et al., 2017).	Causes artery hyperbranching in zebrafish,(Bower et al., 2017)
VEGFR3	Mice die (E9.5) due to pericardial fluid accumulation and vascular remodelling defects (Simons et al., 2016). Postnatal EC specific deletion inhibits Notch signalling, increases angiogenic sprouting, and branching (Tammela et al., 2011).	()
STAT3	Downregulates the expression of *VEGFA* (Niu et al., 2002).	Binds the *VEGFA* promotor, activates its transcription and promotes tumor angiogenesis (Niu et al., 2002).
NRP1	Lethal in mice with defects in the cardiovascular system. Inhibits cell proliferation and angiogenesis in bovine and mice ECs (Oh et al., 2002).	NA.

Supplementary Table 13. The number of micro-environments that induce Tip, Stalk and Phalanx EC behaviors after simulating single gain and loss-of-function mutations

	Tip	Stalk	Phalanx		Tip	Stalk	Phalanx
AKT(gf)	28164	23784	5464	AKT(lf)	50572	13104	0
ALK1(gf)	50944	12288	0	ALK1(lf)	47920	10752	768
ALK5(gf)	50560	12096	96	ALK5(lf)	50572	12096	96
AMPATP(gf)	57152	6816	48	AMPATP(lf)	58956	5280	48
AMPK(gf)	48768	14976	192	AMPK(lf)	55992	7488	96
ANG1(gf)	58054	6048	48	ANG1(lf)	58054	6048	48
ANG2(gf)	50572	12096	96	ANG2(lf)	50572	12096	120
AP1(gf)	50572	12096	96	AP1(lf)	50572	12096	96
Bcatenin(gf)	50572	12812	0	Bcatenin(lf)	50572	8400	96
BMP10(gf)	58240	6144	0	BMP10(lf)	57868	5952	96
BMP9(gf)	58240	6144	0	BMP9(lf)	57868	5952	96
Calcium(gf)	50800	12096	96	Calcium(lf)	47616	14472	144
DLL4a(gf)	50908	12096	96	DLL4a(lf)	0	50496	96
DLL4p(gf)	58052	5824	32	DLL4p(lf)	58056	6272	64
ETS(gf)	50968	12096	96	ETS(lf)	0	50944	128
FAK(gf)	50572	12096	192	FAK(lf)	50560	12264	96
FGF(gf)	58252	6048	48	FGF(lf)	57856	6048	48
FOXO1(gf)	50572	12096	120	FOXO1(lf)	50572	12096	96
HEY1(gf)	50560	12180	96	HEY1(lf)	50576	12096	96
HIF1(gf)	50572	12096	144	HIF1(lf)	50572	12096	96
IGF(gf)	58956	4608	0	IGF(lf)	57152	7488	96
Integrin(gf)	50572	12096	192	Integrin(lf)	50572	12096	96
JAGa(gf)	50572	12812	0	JAGa(lf)	50572	0	768
JAGp(gf)	58056	6272	64	JAGp(lf)	58052	5824	32
KLF2(gf)	50950	12096	192	KLF2(lf)	50572	12096	96
LEF1(gf)	50572	12096	96	LEF1(lf)	50572	12096	96
MEK(gf)	50968	12096	96	MEK(lf)	0	40896	96
NFAT(gf)	50800	12096	96	NFAT(lf)	48768	14472	144
NICD(gf)	51040	10752	0	NICD(lf)	50576	12544	128
NO(gf)	50560	12180	96	NO(lf)	50800	12096	96
NOTCH(gf)	50912	10752	0	NOTCH(lf)	50576	12544	128
NRP1(gf)	65024	0	0	NRP1(lf)	0	59904	1068
NRARP(gf)	50576	12544	128	NRARP(lf)	50588	12096	96
Oxygen(gf)	58956	5280	48	Oxygen(lf)	57152	6816	48
p38MAPK(gf)	50560	12096	96	p38MAPK(lf)	50572	12096	96
PECAM1(gf)	50956	12096	168	PECAM1(lf)	50560	12180	108
PIP3(gf)	28164	23784	5464	PIP3(lf)	50572	13104	0
PLCg(gf)	51200	12096	96	PLCg(lf)	48640	12180	96
RAS(gf)	50956	12096	96	RAS(lf)	46220	12096	96
ShearStress(gf)	58368	6048	96	ShearStress(lf)	57740	6048	0
SIRT1(gf)	50560	12096	120	SIRT1(lf)	50572	12096	96
SMAD1(gf)	50560	12920	0	SMAD1(lf)	50572	10752	768
SMAD2(gf)	50560	12180	96	SMAD2(lf)	50572	12096	96
SMAD6(gf)	50572	10752	768	SMAD6(lf)	50572	12544	96
SRC(gf)	50572	12096	192	SRC(lf)	50572	12516	96
STAT3(gf)	50572	12096	96	STAT3(lf)	50572	12096	96
TGFB1(gf)	58240	6144	0	TGFB1(lf)	57868	5952	96
TIE2(gf)	50572	12096	192	TIE2(lf)	50572	12096	96
TSC(gf)	50572	12096	96	TSC(lf)	50572	12096	96
VEcadherin(gf)	50956	12096	192	VEcadherin(lf)	50560	12096	96
VegfA(gf)	50572	12096	96	VegfA(lf)	48768	14976	192
VEGFAxxx(gf)	65024	0	0	VEGFAxxx(lf)	32512	29952	384
VEGFAxxxA(gf)	65024	0	0	VEGFAxxxA(lf)	48768	14976	192
VEGFAxxxd(gf)	50560	12096	96	VEGFAxxxd(lf)	50572	12096	96
VEGFAxxxP(gf)	65280	0	0	VEGFAxxxP(lf)	50828	12096	96
VEGFC_D(gf)	58054	6048	48	VEGFC_D(lf)	58054	6048	48
VEGFC_Dp(gf)	65280	0	0	VEGFC_Dp(lf)	50828	12096	96
Vegfr2(gf)	50672	12096	96	Vegfr2(lf)	50560	12180	96
VEGFR22(gf)	51200	12096	192	VEGFR22(lf)	50560	12180	96
VEGFR23(gf)	50956	12096	192	VEGFR23(lf)	50572	12096	96
Vegfr3(gf)	51088	12096	96	Vegfr3(lf)	50572	12096	96
VEGFR33(gf)	51200	12096	96	VEGFR33(lf)	50572	12096	96
WNT5a(gf)	58368	7168	0	WNT5a(lf)	57740	4928	96
WNT7a(gf)	58368	7168	0	WNT7a(lf)	57740	4928	96

Supplementary Table 14. The mutations that decrease or do not affect the number of micro-environments that induce Tip, Stalk, and Phalanx EC behavior

Micro-environments	Tip	Stalk	Phalanx
None	DLL4a(lf), ETS(lf), MEK(lf), NRP1(lf)	JAGa(lf), NRP1(gf), VEGFAxxx(gf), VEGFAxxxA(gf), VEGFAxxxP(gf), VEGFC_Dp(gf)	AKT(lf), ALK1(gf), Bcatenin(gf), BMP10(gf), BMP9(gf), IGF(gf), JAGa(gf), NICD(gf), NOTCH(gf), NRP1(gf), PIP3(lf), ShearStress(lf), SMAD1(gf), TGFB1(gf), VEGFAxxx(gf), VEGFAxxxA(gf), VEGFAxxxP(gf), VEGFC_Dp(gf), WNT5a(gf), WNT7a(gf)
Less than the wild type	AKT(gf), ALK1(lf), ALK5(gf), AMPK(gf), Calcium(lf), FAK(lf), HEY1(gf), NFAT(lf), NO(gf), p38MAPK(gf), PECAM1(lf), PIP3(gf), PLCg(lf), RAS(lf), SIRT1(gf), SMAD1(gf), SMAD2(gf), Vecadherin(lf), VegfA(lf), VEGFAxxx(lf), VEGFAxxxA(lf), VEGFAxxxd(gf), Vegfr2(lf), VEGFR22(lf)	ALK1(lf), AMPATP(gf), AMPATP(lf), AMPK(lf), ANG1(gf), ANG1(lf), Bcatenin(lf), BMP10(gf), BMP10(lf), BMP9(gf), BMP9(lf), DLL4p(gf), DLL4p(lf), FGF(gf), FGF(lf), IGF(gf), IGF(lf), JAGp(gf), JAGp(lf), NICD(gf), NOTCH(gf), Oxygen(gf), Oxygen(lf), ShearStress(gf), ShearStress(lf), SMAD1(lf), SMAD6(gf), TGFB1(gf), TGFB1(lf), VEGFC_D(gf), VEGFC_D(lf), WNT5a(gf), WNT5a(lf), WNT7a(gf), WNT7a(lf)	AMPATP(gf), AMPATP(lf), ANG1(gf), ANG1(lf), DLL4p(gf), DLL4p(lf), FGF(gf), FGF(lf), JAGp(gf), JAGp(lf), Oxygen(gf), Oxygen(lf), VEGFC_D(gf), VEGFC_D(lf)
Same as the wild type	AKT(lf), ALK5(lf), ANG2(gf), ANG2(lf), AP1(gf), AP1(lf), Bcatenin(gf), Bcatenin(gf), FAK(gf), FOXO1(gf), FOXO1(lf), HIF1(gf), HIF1(lf), Integrin(gf), Integrin(lf), JAGa(gf), JAGa(lf), KLF2(lf), LEF1(gf), LEF1(lf), p38MAPK(lf), PIP3(lf), SIRT1(lf), SMAD1(lf), SMAD2(lf), SMAD6(gf), SMAD6(lf), SRC(gf), SRC(lf), STAT3(gf), STAT3(lf), TIE2(gf), TIE2(lf), TSC(gf), TSC(lf), VegfA(gf), VEGFAxxxd(lf), VEGFR23(lf), Vegfr3(lf), VEGFR33(lf)	ALK5(gf), ALK5(lf), ANG2(gf), ANG2(lf), AP1(gf), AP1(lf), Calcium(gf), DLL4a(gf), ETS(gf), FAK(gf), FOXO1(gf), FOXO1(lf), HEY1(lf), HIF1(gf), HIF1(lf), Integrin(gf), Integrin(lf), KLF2(gf), KLF2(lf), LEF1(gf), LEF1(lf), MEK(gf), NFAT(gf), NO(lf), NRARP(lf), p38MAPK(gf), p38MAPK(lf), PECAM1(gf), PLCg(gf), RAS(gf), RAS(lf), SIRT1(gf), SIRT1(lf), SMAD2(lf), SRC(gf), STAT3(gf), STAT3(lf), TIE2(gf), TIE2(lf), TSC(gf), TSC(lf), Vecadherin(gf), VEcadherin(lf), VegfA(gf), VEGFAxxxd(gf), VEGFAxxxd(lf), VEGFAxxxP(lf), VEGFC_Dp(lf), Vegfr2(gf), VEGFR22(gf), VEGFR23(gf), VEGFR23(lf), Vegfr3(gf), Vegfr3(lf), VEGFR33(gf), VEGFR33(lf)	ALK5(gf), ALK5(lf), AMPK(lf), ANG2(gf), AP1(gf), AP1(lf), Bcatenin(lf), BMP10(lf), BMP9(lf), Calcium(gf), DLL4a(gf), DLL4a(lf), ETS(gf), FAK(lf), FOXO1(lf), HEY1(gf), HEY1(lf), HIF1(lf), IGF(lf), Integrin(lf), KLF2(lf), LEF1(gf), LEF1(lf), MEK(gf), MEK(lf), NFAT(gf), NO(gf), NO(lf), NRARP(lf), p38MAPK(gf), p38MAPK(lf), PLCg(gf), PLCg(lf), RAS(gf), RAS(lf), ShearStress(gf), SIRT1(lf), SMAD2(gf), SMAD2(lf), SMAD6(lf), SRC(lf), STAT3(gf), STAT3(lf), TGFB1(lf), TIE2(lf), TSC(gf), TSC(lf), VEcadherin(lf), VegfA(gf), VEGFAxxxd(gf), VEGFAxxxd(lf), VEGFAxxxP(lf), VEGFC_Dp(lf), Vegfr2(gf), Vegfr2(lf), VEGFR22(lf), VEGFR23(lf), Vegfr3(gf), Vegfr3(lf), VEGFR33(gf), VEGFR33(lf), WNT5a(lf), WNT7a(lf)

Supplementary Table 15. The mutations that increase the number of micro-environments that induce Tip, Stalk, and Phalanx EC behavior

Micro-environments	Tip	Stalk	Phalanx
More than the wild type	ALK1(gf), AMPATP(gf), AMPATP(lf), AMPK(lf), ANG1(gf), ANG1(lf), BMP10(gf), BMP10(lf), BMP9(gf), BMP9(lf), Calcium(gf), DLL4a(gf), DLL4p(gf), DLL4p(lf), ETS(gf), FGF(gf), FGF(lf), HEY1(lf), IGF(gf), IGF(lf), JAGp(gf), JAGp(lf), KLF2(gf), MEK(gf), NFAT(gf), NICD(gf), NICD(lf), NO(lf), NOTCH(gf), NOTCH(lf), NRP1(gf), NRARP(gf), NRARP(lf), Oxygen(gf), Oxygen(lf), PECAM1(gf), PLCg(gf), RAS(gf), ShearStress(gf), ShearStress(lf), TGFB1(gf), TGFB1(lf), VEcadherin(gf), VEGFAxxx(gf), VEGFAxxxA(gf), VEGFAxxxP(gf), VEGFAxxxP(lf), VEGFC_D(gf), VEGFC_D(lf), VEGFC_Dp(gf), VEGFC_Dp(lf), Vegfr2(gf), VEGFR22(gf), VEGFR23(gf), Vegfr3(gf), VEGFR33(gf), WNT5a(gf), WNT5a(lf), WNT7a(gf), WNT7a(lf)	AKT(gf), AKT(lf), ALK1(gf), AMPK(gf), Bcatenin(gf), Calcium(lf), DLL4a(lf), ETS(lf), FAK(lf), HEY1(gf), JAGa(gf), MEK(lf), NFAT(lf), NICD(lf), NO(gf), NOTCH(lf), NRP1(lf), NRARP(gf), PECAM1(lf), PIP3(gf), PIP3(lf), PLCg(lf), SMAD1(gf), SMAD2(gf), SMAD6(lf), SRC(lf), VegfA(lf), VEGFAxxx(lf), VEGFAxxxA(lf), Vegfr2(lf), VEGFR22(lf)	AKT(gf), ALK1(lf), AMPK(gf), ANG2(lf), Calcium(lf), ETS(lf), FAK(gf), FOXO1(gf), HIF1(gf), Integrin(gf), JAGa(lf), KLF2(gf), NFAT(lf), NICD(lf), NOTCH(lf), NRP1(lf), NRARP(gf), PECAM1(gf), PECAM1(lf), PIP3(gf), SIRT1(gf), SMAD1(lf), SMAD6(gf), SRC(gf), TIE2(gf), VEcadherin(gf), VegfA(lf), VEGFAxxx(lf), VEGFAxxxA(lf), VEGFR22(gf), VEGFR23(gf)

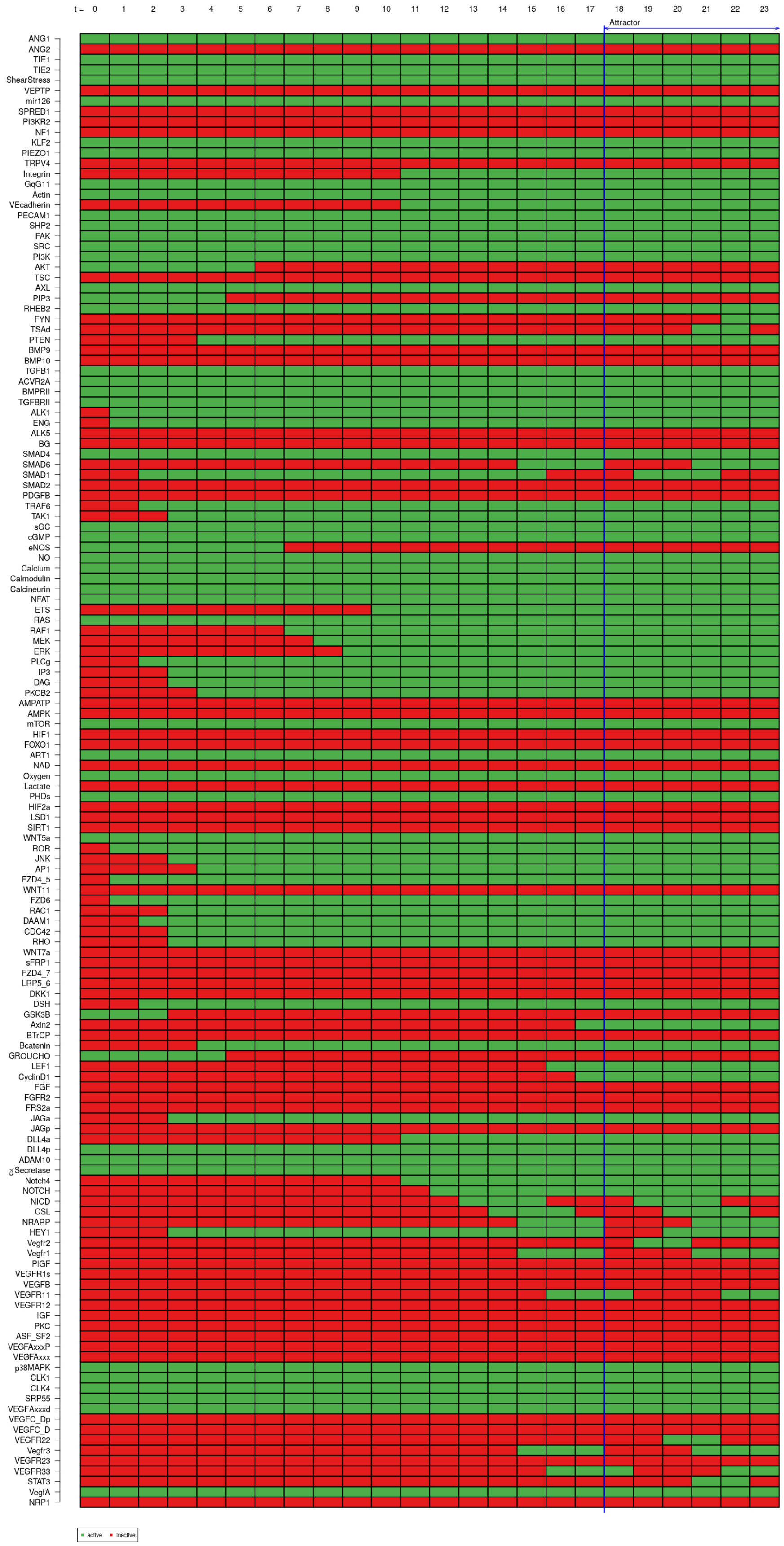

Supplementary Figure 8. Transition from Phalanx to Stalk EC behavior in our extended model.

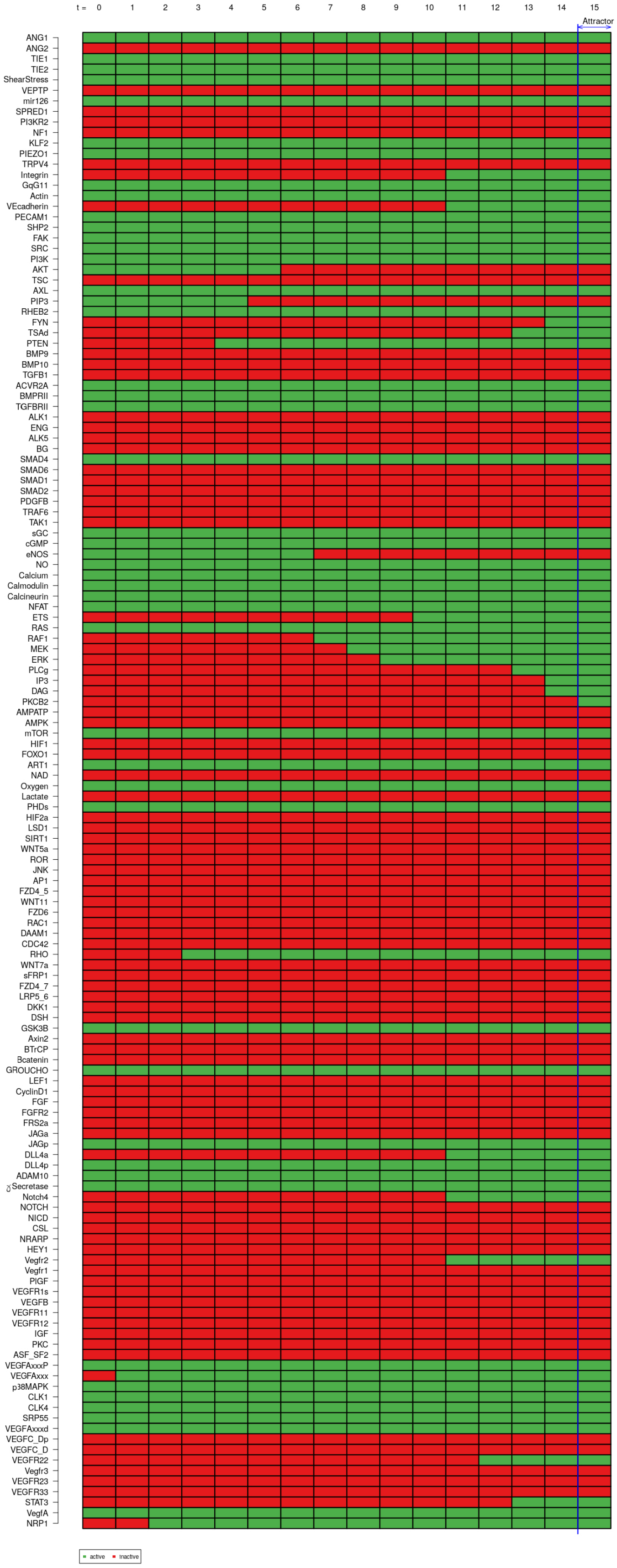

Supplementary Figure 9. Transition from Phalanx to Tip EC behavior in our extended model.

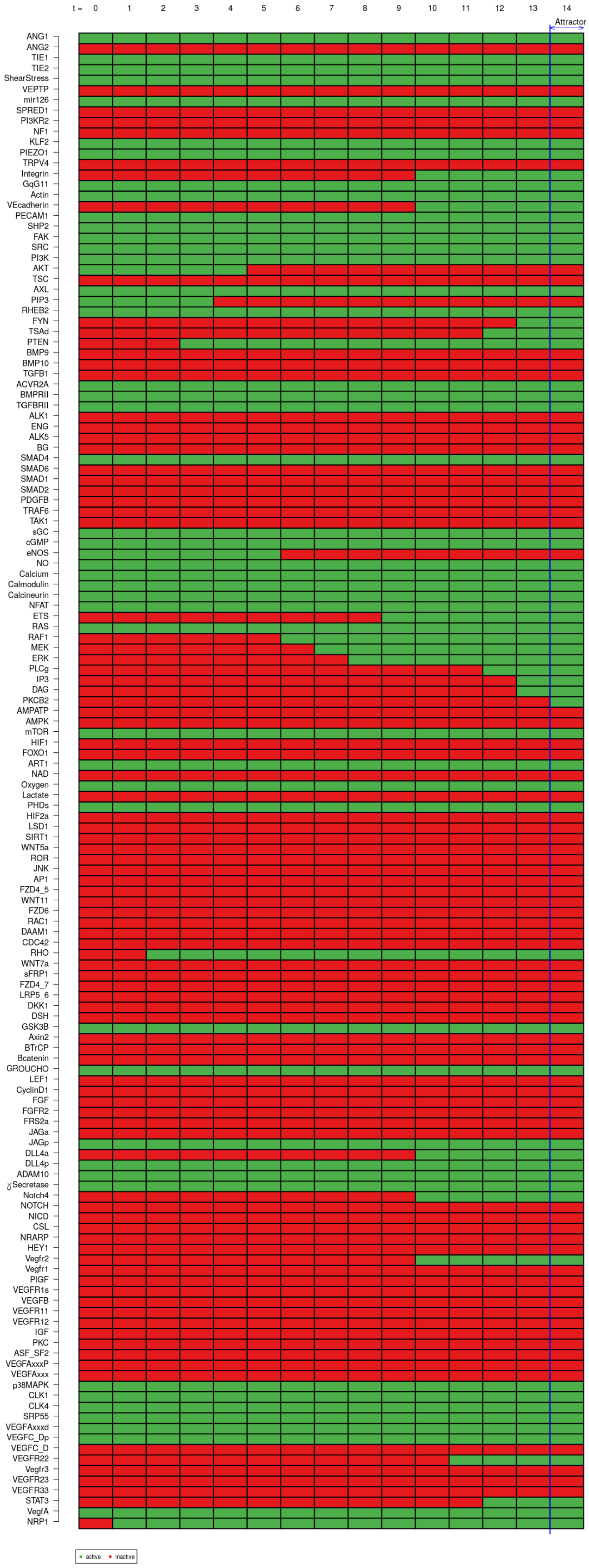

Supplementary Figure 10. Transition from Phalanx to Tip EC behavior with VEGFC or VEGFD in our extended model.

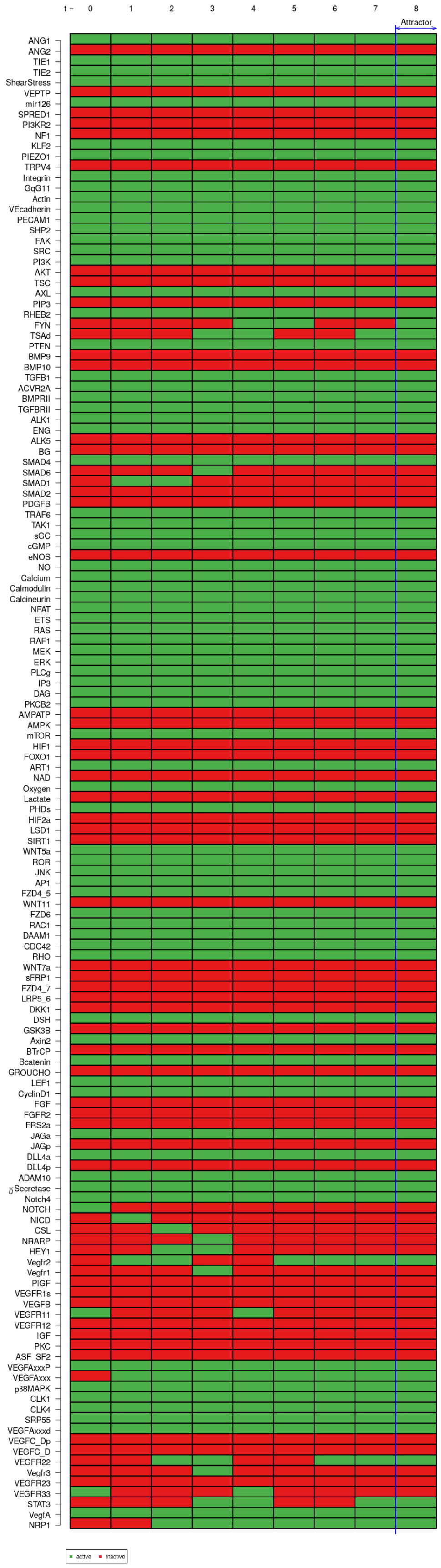

Supplementary Figure 11. Transition from Stalk to Phalanx EC behavior in our extended model.

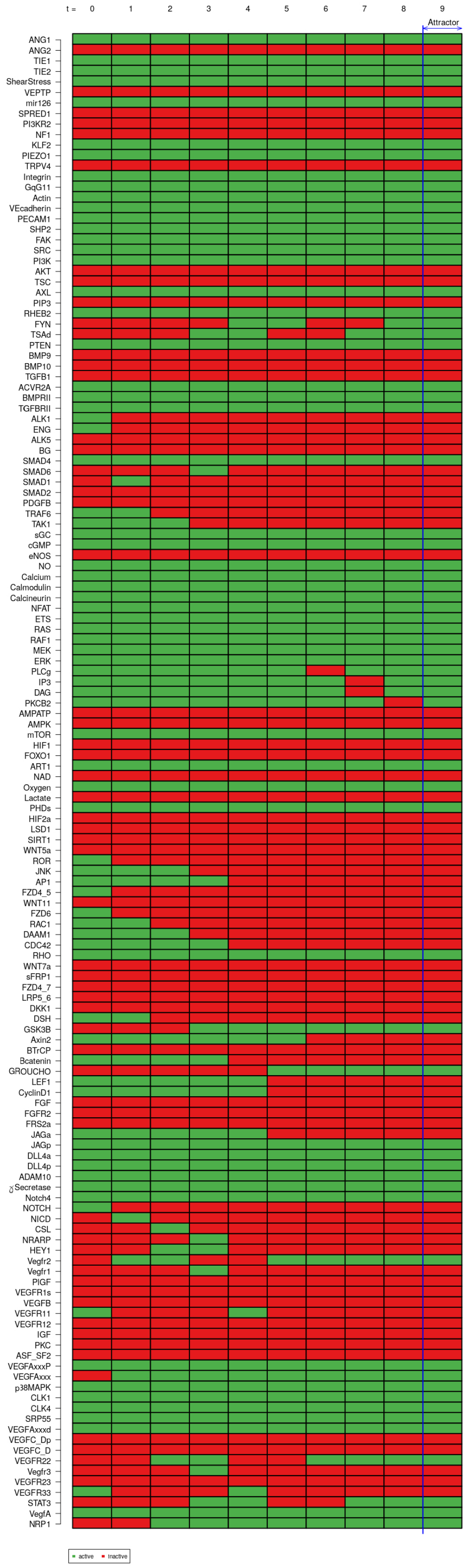

Supplementary Figure 12. Transition from Stalk to Tip EC behavior in our extended model.

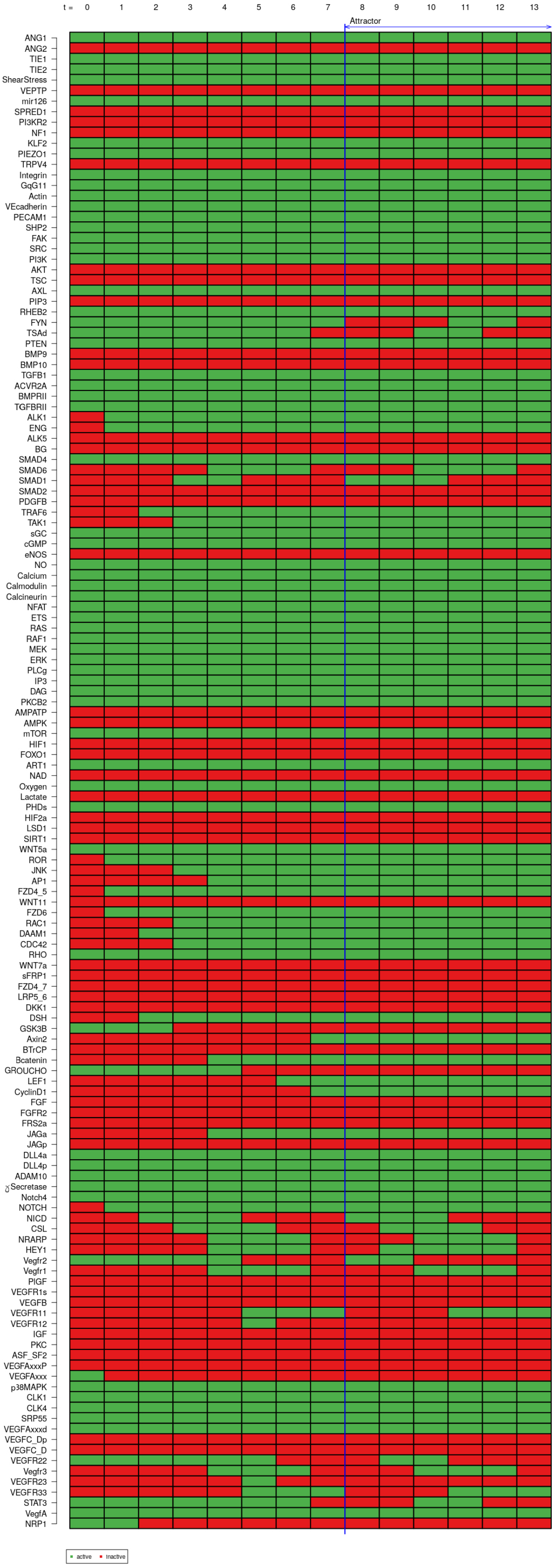

Supplementary Figure 13. Transition from Tip to Stalk EC behavior in our extended model.

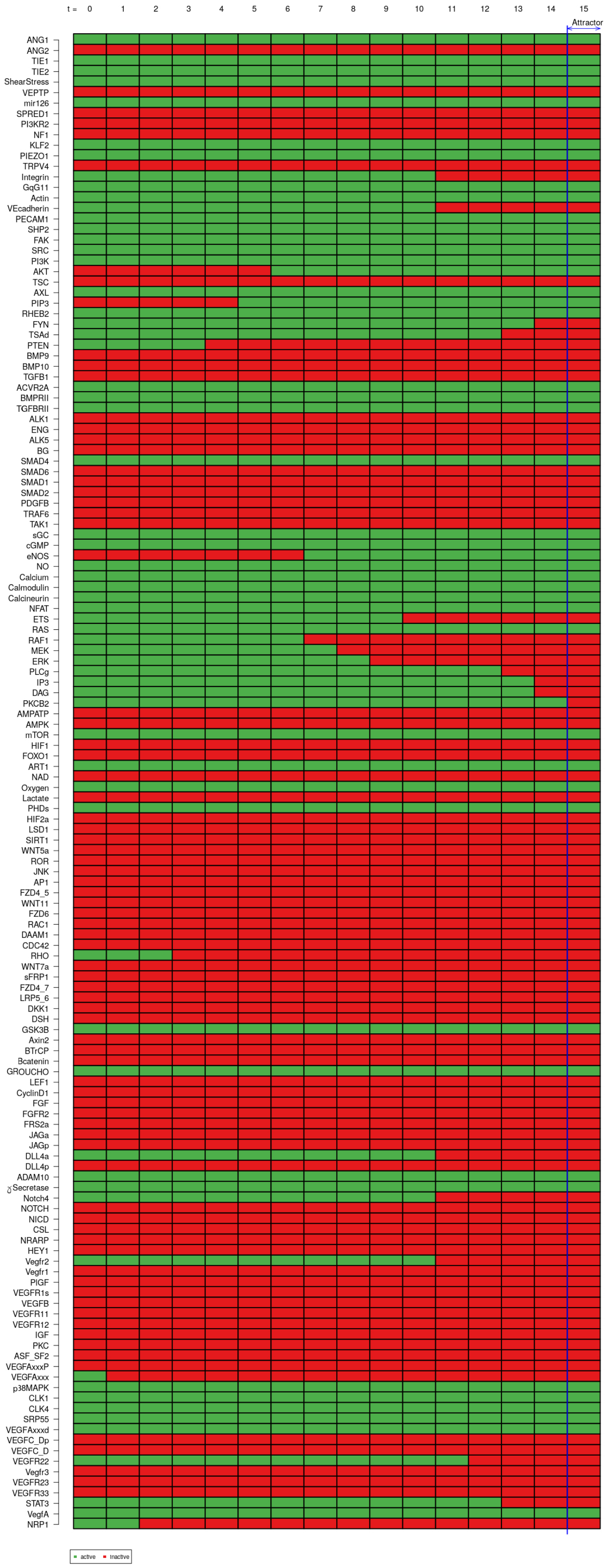

Supplementary Figure 14. Transition from Tip to Phalanx EC behavior in our extended model.

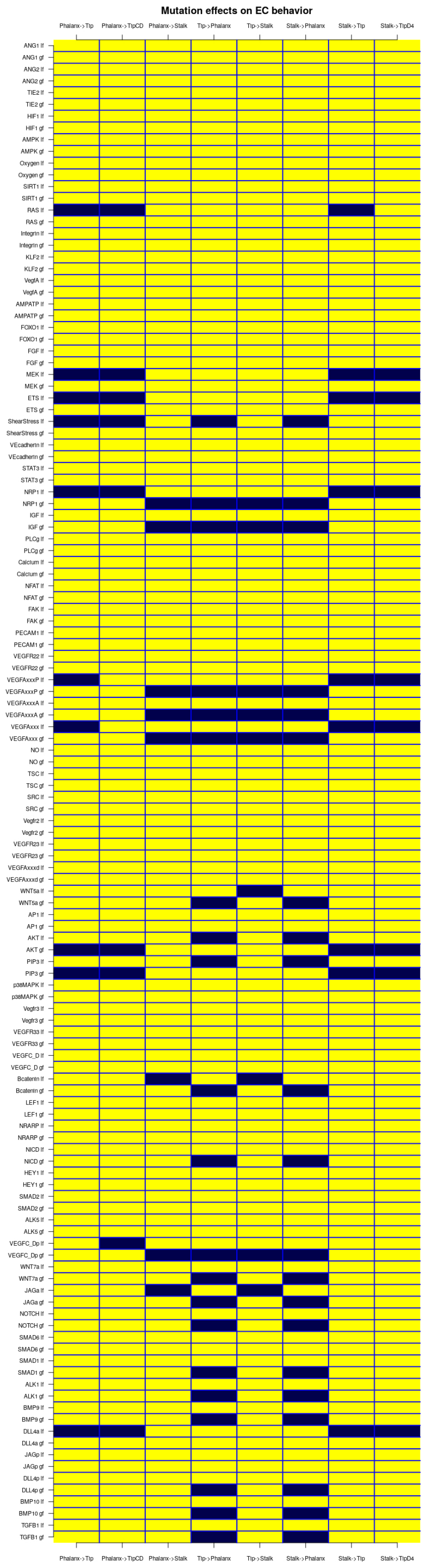

Supplementary Figure 15. Effect of single gain- and loss-of-function mutations on the most likely transitions in EC behavior (Figure 3A in the main text). Inhibited transitions are shown in blue, uninhibited transitions are shown in yellow.

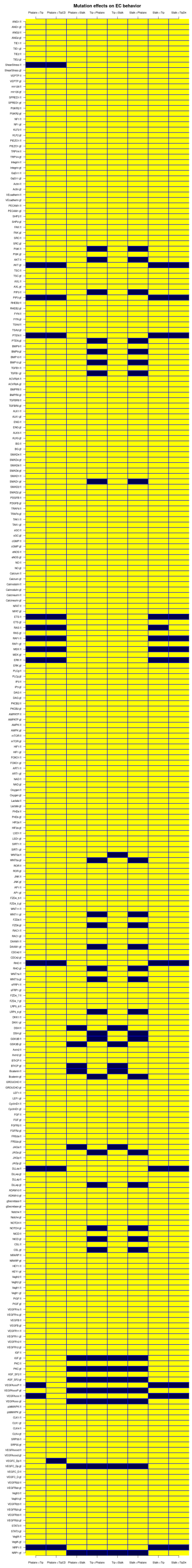

Supplementary Figure 16. Effect of single gain- and loss-of-function mutations on the most likely transitions in EC behavior (Figure 3A in the main text) in our extended model. Inhibited transitions are shown in blue, uninhibited transitions are shown in yellow.

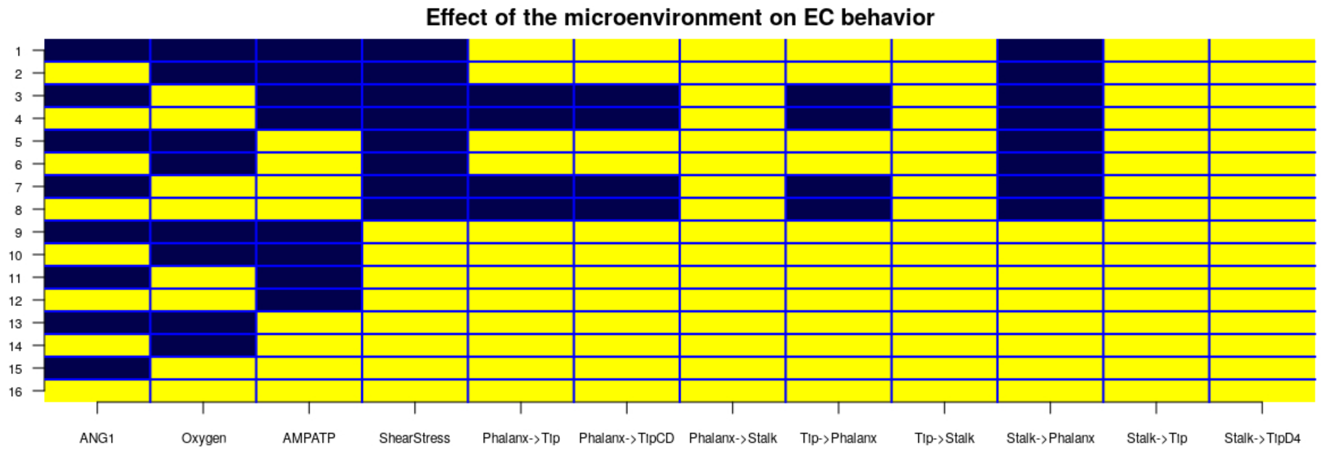

Supplementary Figure 17. Effect of Oxygen, the AMP to ATP ratio and Shear Stress on the most likely transitions in EC behavior (Figure 3A in the main text). Inactive variables and transitions are shown in blue, active variables and transitions are shown in yellow. The experiments where carried out using both our extended and our simplified model, the results where identical.
